# Design, Synthesis, and Biological Evaluation of Novel Acetylcholinesterase and β-Secretase 1 Inhibitors

**DOI:** 10.3390/ijms27021008

**Published:** 2026-01-20

**Authors:** Danuta Drozdowska, Damian Pawelski, Agnieszka Wróbel-Tałałaj, Marta Plonska-Brzezinska, Beata Kolesinska, Ryszard Lazny, Barbara Seroka, Cezary Parzych, Artur Ratkiewicz

**Affiliations:** 1 Department of Organic Chemistry, Medical University of Bialystok, 15-222 Bialystok, Polandagnieszkawrobel9@gmail.com (A.W.-T.); marta.plonska-brzezinska@umb.edu.pl (M.P.-B.); 2 Institute of Organic Chemistry, Lodz University of Technology, 90-924 Lodz, Poland; beata.kolesinska@p.lodz.pl; 3 Department of Polymers and Organic Synthesis, Faculty of Chemistry, University of Bialystok, 15-245 Bialystok, Poland; lazny@uwb.edu.pl (R.L.); b.seroka@uwb.edu.pl (B.S.); 4 Department of Physical Chemistry, Faculty of Chemistry, University of Bialystok, 15-245 Bialystok, Poland; c.parzych@uwb.edu.pl (C.P.); artrat@uwb.edu.pl (A.R.)

**Keywords:** multi-target drugs, enzyme inhibitors, 3-granatanone, pseudopelletierine, Alzheimer’s disease, BACE1 inhibitors, cholinesterase inhibitors

## Abstract

A series of novel granatane–triazole hybrid molecules was designed, synthesized, and evaluated as dual acetylcholinesterase (AChE) and β-secretase 1 (BACE1) inhibitors. The compounds were obtained through a convergent synthetic route involving azide formation, triazole construction via dipolar cycloaddition, and final coupling with a granatane scaffold to give a pseudopelletierine (3-granatanone) analogue. In vitro assays demonstrated that all target compounds inhibited both AChE and BACE1. Molecular docking and molecular dynamics simulations revealed stable interactions with key catalytic residues, suggesting distinct binding modes compared to reference ligands. QSAR-based pharmacokinetic predictions indicated favorable blood–brain barrier permeability and compliance with key drug-likeness filters. These findings identify granatane–triazole hybrids as promising multi-target directed ligand (MTDL) candidates with potential for further optimization in the search for new anti-Alzheimer therapeutics.

## 1. Introduction

The treatment of chronic and multifactorial diseases, including cancer and cardiovascular, neurodegenerative, autoimmune, and metabolic disorders, remains challenging. This difficulty arises from the dysregulation of multiple interconnected disease pathways and the involvement of numerous biological targets [1]. Conventional therapies usually focus on a single molecular target. Such approaches often show limited efficacy and may lead to drug resistance or disease recurrence. Consequently, single-target drugs are frequently insufficient for effective treatment. Multi-target directed ligands (MTDL) address this limitation by enabling the simultaneous modulation of multiple biological targets [2,3,4].

Alzheimer’s disease (AD) is a multifactorial disorder resulting from the complex interaction of many factors, genetic, environmental, psychological, and neurobiological, rather than a single cause. It manifests itself as a progressive neurodegenerative disease characterized by cognitive impairment and irreversible memory loss. It is estimated that by 2050, over 115 million people worldwide will develop AD [5]. Several hypotheses have been proposed to explain the onset and progression of AD. Briefly, AD pathogenesis is explained by the cholinergic hypothesis, involving acetylcholine system dysfunction [6], the amyloid hypothesis, which is based on β-amyloid accumulation [7], and the tau hypothesis, which is associated with abnormal tau phosphorylation and neurofibrillary tangle formation [8]. The development of AD may also be influenced by genetic factors, which contribute to up to 80% of cases [9], neuroinflammation mediated by microglia and astrocytes [10], and oxidative stress resulting from excessive free radical production that promotes neuronal death [11].

There is no causal treatment for Alzheimer’s disease. Its multifactorial nature provides many potential therapeutic targets. The main challenge in developing effective multi-target compounds is identifying the optimal combination of key biological targets. Currently, monotherapies focus mainly on regulating the activity of acetylcholinesterase (AChE), monoamine oxidase (MAO), N-methyl-D-aspartate (NMDA), and serotonin receptors (5-HT, especially 5-HT4R) [12].

So far, the anti-AD agents with the greatest activity in the treatment of AD are cholinesterase inhibitors. An important factor influencing the development of AD is a change in the level of acetylcholine in the cerebral cortex and hippocampus [13]. As the disease progresses, the number of cholinergic neurons in the frontal lobe decreases [14]. Therefore, AChE inhibitors are currently used as a therapeutic approach. Currently available AChE inhibitor drugs approved by the FDA include Donepezil, Galantamine, Tacrine, and Rivastigmine [15,16], and they work by temporarily alleviating the symptoms of the disease and are most effective in mild-to-moderate AD [17]. However, these drugs provide only temporary relief. Currently, however, no available AChE inhibitor prevents the progression of the disease [18,19,20,21]. In addition, the pharmacological effects of AChE inhibitors vary between individuals, and the reason for this phenomenon is unknown [22,23]. This is a serious problem because it makes it impossible to predict the side effects of these drugs in different individuals.

An important enzyme involved in the pathogenesis of AD is β-secretase 1 (Beta site amyloid precursor protein cleaving enzyme 1; BACE-1). It cleaves the transmembrane amyloid precursor protein (APP) in sequence with gamma secretase, producing beta amyloid [24], which constitutes a significant part of the plaques accumulated in AD [25]. It is currently believed that BACE-1 has a direct effect on the production of beta amyloid [26]. Research into effective BACE1 inhibitors has been ongoing for years. For example, BACE1 inhibitors have been developed using stereoselective peptidomimetics based on a 1,4-benzodiazepine core and their analogues. Mono- and bicyclic 6-substituted 2-oxopiperazines and an isophthalamide derivative also exhibited nanomolar activity against BACE1 [27], but to date, no BACE1 inhibitor has been approved by the FDA. All clinical trials of BACE1 inhibitors in AD have now been completed, but this enzyme remains one of the most promising therapeutic targets for slowing down Aβ production and slowing down the progression of AD [28].

A good example of multi-target directed ligands (MTDLs) for AD is dual-target inhibitors, in which AChE is combined with another target related to the disease [29]. In our research, we chose β-secretase (BACE-1) as the second target.

**Figure 1:**
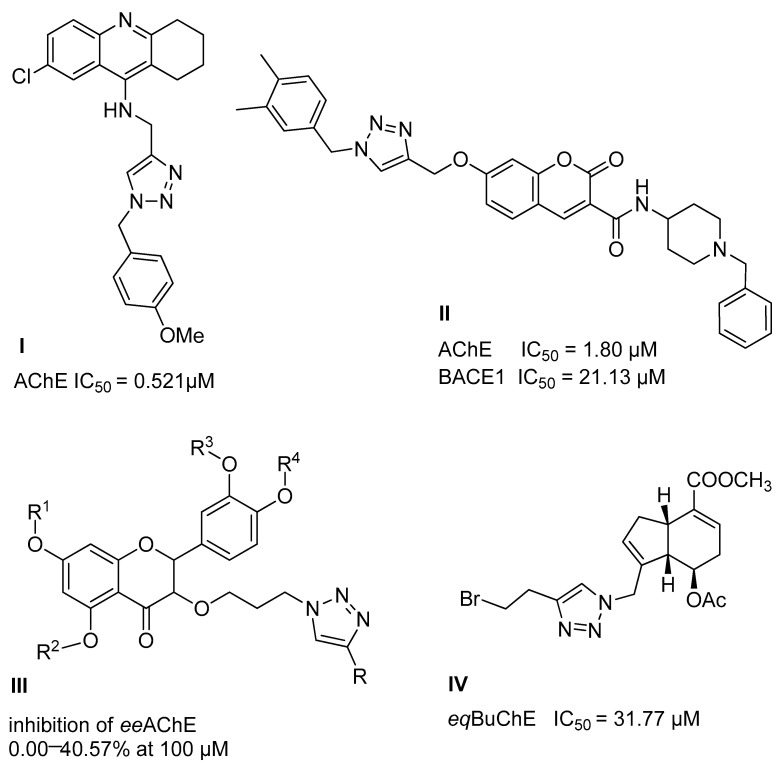
Structures of some hybrid compounds with 1,2,3-triazole.

We planned the synthesis and investigation of 1,2,3-triazole, a five-membered heterocyclic compound containing two carbon atoms and three nitrogen atoms, derivatives. The 1,2,3-triazole ring is found in many compounds with therapeutic properties [30,31]. The triazole ring exhibits bond-accepting properties that enable it to form significant interactions with biomolecular targets through hydrogen bonding, π−π interactions, and dipole interactions. Among the many different biological activities of 1,2,3 triazole derivatives, there was also a series that showed anti-epileptic activity and reduced neuronal hyperactivity. This property may confirm the potential effect of new compounds on enzymes active in AD. [32]. Several hybrid compounds with 1,2,3-triazole are known and have been studied as anti-Alzheimer’s agents. It has been combined with tacrine-1,2,3-triazole hybrid **I** [33], chromenone **II** [34], quercetin **III** [35], and genipin **IV** [36] (Figure 1).

**Figure 2:**
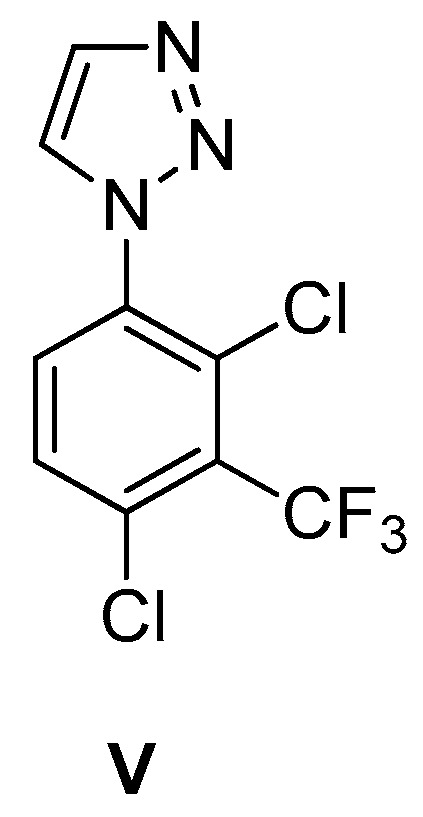
Structures of Notum inhibitor **V**.

Willis et al. described the discovery and profile of compound **V** (ARUK3001185; PDB 7PK3) (Figure 2) as a potent, selective, and brain-penetrant inhibitor of the carboxylesterase Notum [37]. This enzyme suppresses Wnt (Wingless and Int-1 gene family) signaling through deacylation of an essential palmitoleate group on Wnt proteins, and understanding of its role in AD is growing. The triazole derivative **V** strongly inhibited the enzymatic activity of Notum in both biochemical and cellular tests, and it also demonstrated excellent penetration of the brain in rodents.

**Figure 3:**
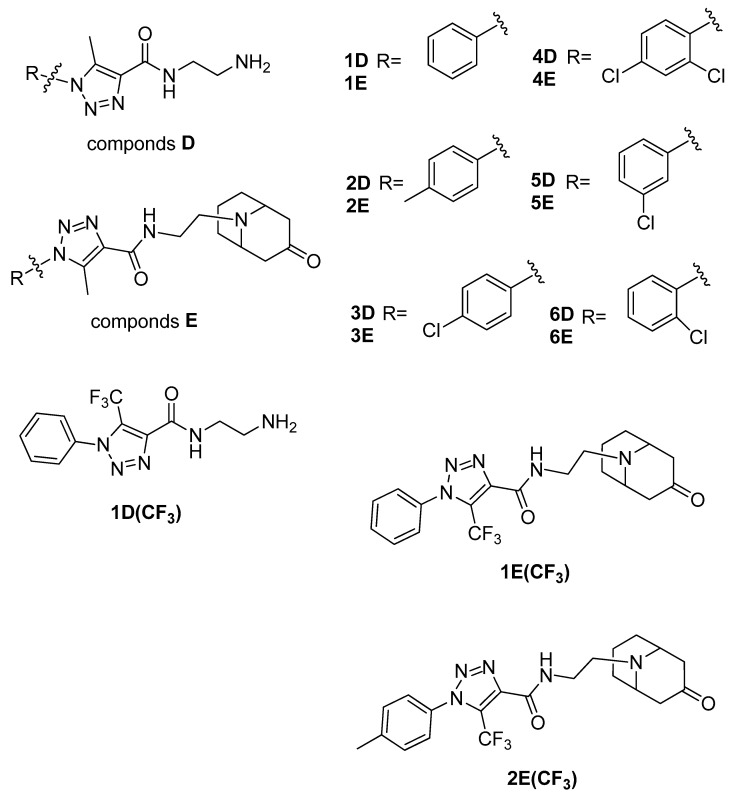
Structures of new compounds tested in this study.

We attempted to create a drug that acts on the Notum protein, using the polarization of the benzene ring connected to triazole by adding chlorine substituents, which affects lipophilicity. The structures of new compounds designed for this study are presented in Figure 3. In a series of compounds **1D–6D, 1–2D(CF_3_)**, combining triazole ring with the granatane-derived skeleton [38,39] could result in a multifunctional molecule capable of crossing the blood–brain barrier. Granatanes and closely related tropane derivatives are known to cross the BBB and exert activity in the CNS [40,41]. Granatane scaffold is also present in some biologically active compounds, including the drug Granisetron, which exhibited some anti-AD potential [42]. Such a combination of triazole ring, amide, tertiary amine, and ketone, and a fairly rigid bicycle, should result in a molecular structure with multiple hydrogen-bond acceptor sites, few rotatable bonds, and other feature advantages for good interaction with receptors. Estimated logP of the proposed molecules are in the range 2.2–3.2, with pKa close to 6.8–7.1. Molecular mass and Topological Polar Surface Area (TPSA) are estimated to be 360–410 and ca. 77, respectively. As such, the new compounds meet the basic (early) criteria for investigational drug candidates. In our opinion, molecules constructed from two parts characterized by limited flexibility (few or no freely rotatable bonds), connected by a flexible tether, may have potential for dual-target ligands.

## 2. Results

### 2.1. Chemistry

**Scheme 1:**
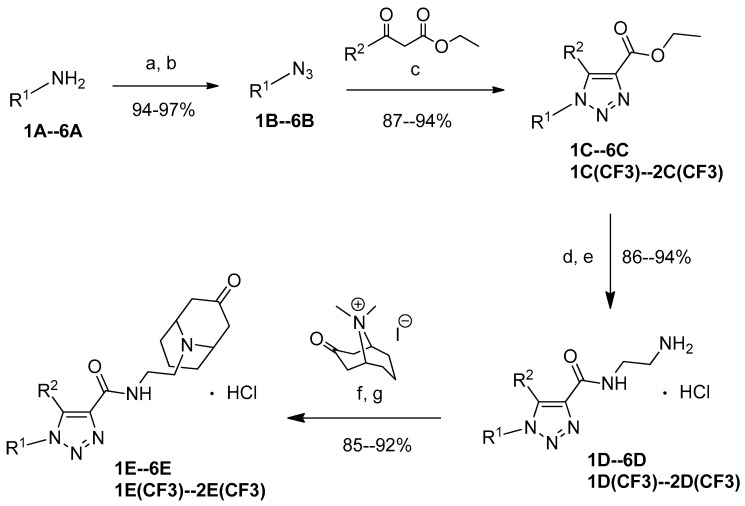
The synthesis of the compounds **1E–6E**, **1E(CF_3_),** and **2E(CF_3_)**. Reagents and conditions: (a) NaNO_2_ (1.1 equiv.), HCl (5 equiv.), H_2_O, 0 °C, 30 min; (b) NaN_3_ (1.1 equiv.), H_2_O, 0 °C, 1 h; (c) DBU (1–2 mol%), K_2_CO_3_ (10 mol%), DMSO, rt, 1–12 h; (d) Ethylenediamine (excess), reflux, 4 h; (e) HCl, MeOH; (f) EtOH/H_2_O (1/1; *v*/*v*), K_2_CO_3_ (3.0 equiv.), MW, 100 °C, 2 h; and (g) HCl, MeOH.

The compounds for biological testing were obtained using a route shown in Scheme 1 and Table 1. Starting from available aromatic amines **1A–6A**, and using the Sandmeyer reaction [43] in an aqueous medium and environmentally benign organic solvents, we obtained aromatic azides **1B–6B** (**GP1**). The reactions were highly efficient (94–97%) and yielded products **1B–6B** of satisfactory purity, which allowed using the obtained compounds directly in the next stage of the synthesis.

The next stage called for the formation of the 1,2,3-triazole system that most often relies on the 1,3-dipolar cycloaddition reaction of azides with terminal alkynes in the presence of a copper(I) salt as a catalyst (the Huisgen reaction) [44]. The copper catalyst used can lead to problems with obtaining desirable purity for biological tests and pharmaceutical applications (formation of coordination complexes with the reaction products, and complicated isolation when these complexes cannot be removed during the extraction stage) [45].

However, the dipolar cycloaddition of azides proceeds surprisingly well with 1,3-dicarbonyl reagents owing to the substantial enolization and increased regent polarization [44]. Therefore, using cheap, non-toxic, and readily available ethyl acetoacetate as the dipolarophile [46], we were able to obtain 1,2,3-triazoles **1C–6C** by a procedure modified from the literature (**GP2**) [47].

Optimization of the procedure revealed that the addition of 1 mol% of an organic base, 1,8-diazabicyclo [5.4.0]undec-7-en (DBU), in addition to the originally used K_2_CO_3_ (10 mol%), shortened the reaction time from 2 h to 30 min. The advantage of this procedure is the use of a non-toxic reaction medium (DMSO) and isolation of the products by precipitation with water, followed by filtering. The reaction is not very sensitive to contamination or contact with the atmosphere.

To test the modification of lipophilicity and electronic properties of compounds **1C** and **2C**, corresponding trifluoromethyl-substituted 1,2,3-triazole **1C(CF_3_)** and **2C(CF_3_)** were prepared by a reaction of aromatic azides **1B** and **2B** with ethyl 4,4,4-trifluoroacetoacetate (**GP3**). Unfortunately, satisfactory reaction progress was achieved only with external heating of the reaction mixture (120 °C for 3 h). Isolation of the CF_3_-substituted product required both extraction and chromatographic purification. Because the involved procedure (**GP3)** was more demanding and replacing the methyl group with its trifluoromethyl analogue resulted in comparable inhibitory activity (*vide infra*), only two representative compounds bearing the lipophilic –CF_3_ group, **1E(CF_3_)** and **2E(CF_3_)**, were prepared for this study.

In the next stage, aromatic esters of 1,2,3-triazole **1C-6C**, **1C(CF_3_)** and **2C(CF_3_)** were subjected to aminolysis with 1,2-ethylenediamine (1,2-EA). The use of up to a 15-fold excess of the amine and high temperature led to complete conversion in 2 h. It was found that the addition of 1 mol% DBU as a catalyst was advantageous [48]. The isolated crude amides with free amine groups were converted into hydrochlorides and purified by crystallization to give **1D–6D**, **1D(CF_3_)**, and **2D(CF_3_)** HCl in good overall yields (86–94%).

In the final step, the granatanone moiety was incorporated via a sequence of elimination and addition reactions starting from a quaternary ammonium salt of granatanone (specifically, pseudopelletierin methiodide was used as the reagent). The reaction was driven by microwave heating in an aqueous ethanol medium. The whole process involved two sequences of base-induced and heat-driven eliminations of a quaternary ammonium group from the β-position of the ketone, leading to the transient formation of an enol group, followed by aza-Michael-type additions (first intermolecular, then intramolecular) to the resulting enone, thereby restoring the N-substituted granatane skeleton. The overall process was very efficient, and the reaction and extraction media used were environmentally benign; nonetheless, the purification before conversion to amines hydrochlorides involved chromatography.

All structures and identities of the new compounds obtained were confirmed using ^1^H and ^13^C NMR spectroscopy and mass spectrometry (see Supplementary Files (Figures S1–S76)). The NMR spectra (^1^H and even more so ^13^C) of the salts of the final granatane derivatives showed splitting of most or all of their signals. Contrary to the static situation and the observed axial position of the N-methyl substituent in the piperidinone ring of crystalline granatanone [49] in solution, the free inversion at nitrogen leads to the presence of both N-invertomers. As it has been previously described for the granatane and tropane series [50,51], the splitting is indicative of the presence of configurationally stable (in the NMR time scale) forms of protonated tertiary amines. The signals are often of comparable intensity and indicate a similar order of magnitude to the energies of the two amine invertomers attainable by the granatane bicycle. This can be advantageous for a multi-target ligand, as it should result in tolerance of steric demands and characteristics of the relevant receptor.

### 2.2. In Vitro AChE and BACE1 Inhibitory Activity

**Figure 4:**
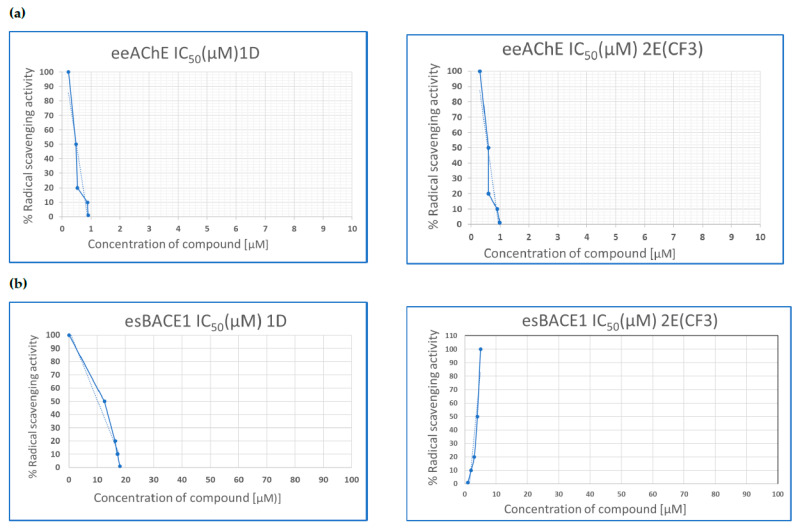
Concentration-dependent effects of compounds **1D** and **2E(CF_3_)** on AChE (**a**) and BACE1 (**b**) enzyme activity.

The inhibitory activity of the compounds obtained against AChE and BACE1 enzymes was assessed in vitro using the methods described previously [52,53] and compared to the activity of standards (Donepezil, Tacrine, Quercetin). The experiments were repeated three times, and the calculated concentration inhibiting half of the enzymatic activity, i.e., IC_50_ (µM), is given in Table 2. All compounds were active against the studied enzymes, and the calculated IC_50_ values ranged from 0.568 to 1.326 μM for AChE and from 9.25 to 28.44 μM for BACE1. None of the newly synthesized substances exhibited higher activity against AChE than the reference compound donepezil (IC_50_ = 0.046 μM); the most active among them was compound **4D**, with an IC_50_ value of 0.555 μM. The strongest inhibition of BACE1 was observed for compound **2E(CF_3_)** (IC_50_ = 9.25 μM); however, its inhibitory activity was approximately half that of the reference compound quercetin (IC_50_ = 4.89 μM). Given the considerable variation in AChE inhibitory activity among the compounds, compounds **1D** and **2E(CF_3_)** were selected for further analysis. Figure 4 presents the concentration-dependent effects of compounds **1D** and **2E(CF_3_)** on AChE and BACE1 enzyme activity.

Detailed inspection of the inhibition data in Table 2 reveals several structure–activity relationship (SAR) trends across the E- and D-series compounds. Within the E-series (**1E**–**6E**), substitution patterns exert a measurable but moderate influence on AChE inhibition, with IC_50_ values ranging from 0.699 µM (**5E**) to 1.326 µM (**4E**). This relatively narrow activity window suggests that the core scaffold dominates AChE binding, while peripheral substituent effects fine-tune potency. Introduction of a trifluoromethyl group leads to a consistent improvement in activity. Both **1E(CF_3_)** and **2E(CF_3_)** display lower IC_50_ values toward AChE compared to their non-CF_3_ analogues **1E** and **2E**, with **2E(CF_3_)** (0.599 µM) being the most active compound in the E-series. A similar trend is observed for BACE1 inhibition, where **2E(CF_3_)** exhibits the lowest IC_50_ value among all tested E-series derivatives (9.25 µM). This enhancement may be attributed to the strong electron-withdrawing nature of the CF_3_ group combined with its ability to engage in additional non-classical interactions, thereby improving binding complementarity.

In the D-series (**1D**–**6D**), the inhibitory activity toward AChE is generally stronger than in the corresponding E-series, with IC_50_ values clustered between 0.555 µM (**4D**) and 0.655 µM (**6D**). Among these, **5D** (0.568 µM) represents the most potent D-series inhibitor of AChE. The CF_3_-substituted derivative **1D(CF_3_)** shows slightly reduced AChE activity compared to **1D**, indicating that CF_3_ substitution is not universally beneficial within this scaffold and that steric or conformational effects may counterbalance favorable electronic contributions.

Overall, the SAR analysis indicates that CF_3_ substitution is particularly advantageous for BACE1 inhibition, while the D-series scaffold (**1D**–**6D**) is intrinsically better suited for AChE inhibition. These trends highlight the delicate balance between electronic effects, steric demand, and scaffold rigidity in achieving dual AChE/BACE1 activity.

### 2.3. Molecular Docking

To better understand the binding preferences and interaction profiles of the synthesized ligands, molecular docking studies were performed against AChE and BACE1. This computational analysis enabled evaluation of binding energy, interaction patterns, and conformational flexibility in comparison with reference inhibitors. The obtained docking results were then correlated with the experimental inhibitory activities to support structure–activity interpretation; however, it should be emphasized that these docking analyses are not supported by direct experimental validation, such as site-directed mutagenesis or detailed kinetic inhibition studies, and, therefore, should be interpreted as qualitative models of plausible binding modes rather than definitive mechanistic evidence. Compounds **1D**–**6D**, **1E**–**6E**, **1D(CF_3_)**, **1E(CF_3_)**, and **2E(CF_3_)**, along with the reference ligands Donepezil, Tacrine, and Quercetin, were subjected to molecular docking. The results are summarized in Table 2. For both AChE and BACE1, the tested ligands have higher experimental IC_50_ values compared to the reference ligands, although the docking energies are more favorable for triazoles in some cases. In addition, the docking energies of compounds with a bicyclic ring are approximately 3 kcal/mol lower (less negative calculated values) than their analogues without this ring. A lower IC_50_ value is observed for compounds containing a trifluoromethyl group. The presence of this group enables interactions between fluorine atoms and the protein, e.g., hydrogen bonds, which are absent in other complexes studied. Due to their relatively low IC_50_ and low docking energies for both enzymes, **1D** and **2E_CF3_** were selected for further study.

Comparison of docking scores and experimental IC_50_ values for compounds **1E**–**6E**, **1E(CF_3_), 2E(CF_3_)**, and **1D**–**6D** indicates that docking energies alone do not reliably predict inhibitory potency. For example, **2E(CF_3_)** exhibits one of the most favorable docking scores for both AChE and BACE1, which is consistent with its strong BACE1 inhibition (IC_50_ = 9.25 µM) but only moderately better AChE activity relative to other D-series compounds such as **4D** or **5D**. Conversely, compounds such as **5D** and **4D** show strong AChE inhibition despite docking energies that are less favorable than those calculated for Donepezil. This discrepancy underscores the limitation of docking scores as approximations of static binding affinity that do not account for dynamic effects, entropic contributions, or solvent interactions present under experimental conditions.

Furthermore, several E-series compounds (e.g., **1E**–**3E**) display very similar docking energies and yet differ noticeably in their experimental IC_50_ values, reinforcing the conclusion that docking primarily identifies feasible binding modes rather than providing reliable quantitative activity rankings. Consequently, docking results in the present study should be interpreted as qualitative support for binding plausibility and interaction patterns and must be contextualized using complementary metrics that better account for size and efficiency effects. In this regard, ligand efficiency offers a more informative framework by normalizing binding energy with respect to molecular size. Among the E-series, compounds **1E**–**6E** exhibit tightly clustered AChE ligand efficiencies (approximately −0.39 to −0.41 kcal/mol·atom), with **1E** and **2E** performing near the upper end of this range, whereas the CF_3_-substituted analogues **1E(CF_3_)** and **2E(CF_3_)** show slightly reduced efficiencies due to their increased molecular size, despite improved docking scores. In contrast, the D-series compounds display more negative ligand efficiencies, reaching values as low as −0.458 kcal/mol·atom for **1D**, which indicates more effective per-atom binding to AChE relative to their E-series counterparts. For BACE1, ligand efficiencies are uniformly weaker (approximately −0.26 to −0.38 kcal/mol·atom), with **1D**, Tacrine, and Quercetin showing the most favorable per-atom efficiencies, while **1E**–**6E** and their CF_3_-substituted derivatives contribute only modestly to binding on a per-atom basis.

**Figure 5:**
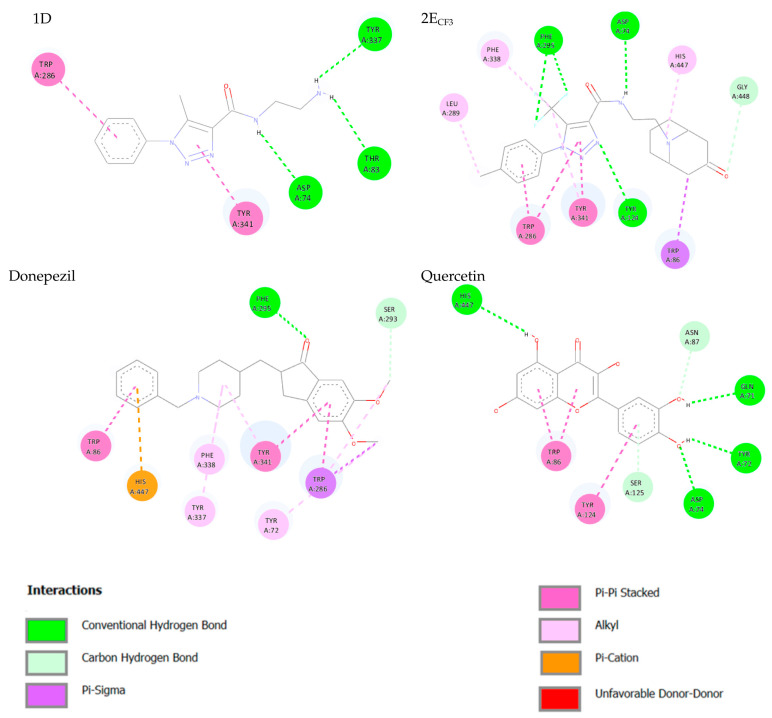
2D diagrams of ligand interactions with AChE.

Figure 5 shows diagrams of the interactions between AChE and the tested compounds.

**1D** forms three hydrogen bonds: one via the hydrogen atom of the amide group with Asp-74 and two via the amino group with Thr-83 and Tyr-337. Additionally, it engages in hydrophobic interactions involving aromatic rings. **2E(CF_3_)**, in turn, forms four hydrogen bonds: analogous to **1D**, a bond with Asp-74, a bond involving the nitrogen atom in the aromatic ring and Tyr-124, as well as two bonds between Phe-295 and fluorine atoms. In addition, there are hydrophobic and π-σ interactions. Donepezil forms only one hydrogen bond between Phe-295 and the oxygen atom of the carbonyl group. The rest of its interactions with the protein are hydrophobic, π-σ, and π-cation. Quercetin, on the other hand, in addition to hydrophobic interactions, forms four hydrogen bonds with Gln-71, Tyr-72, Asp-74, and His-447—all via hydroxyl groups. Based on the docking results, it can be concluded that the key residues are Asp-74 and Phe-295, as bonds with them are observed most frequently.

**Figure 6:**
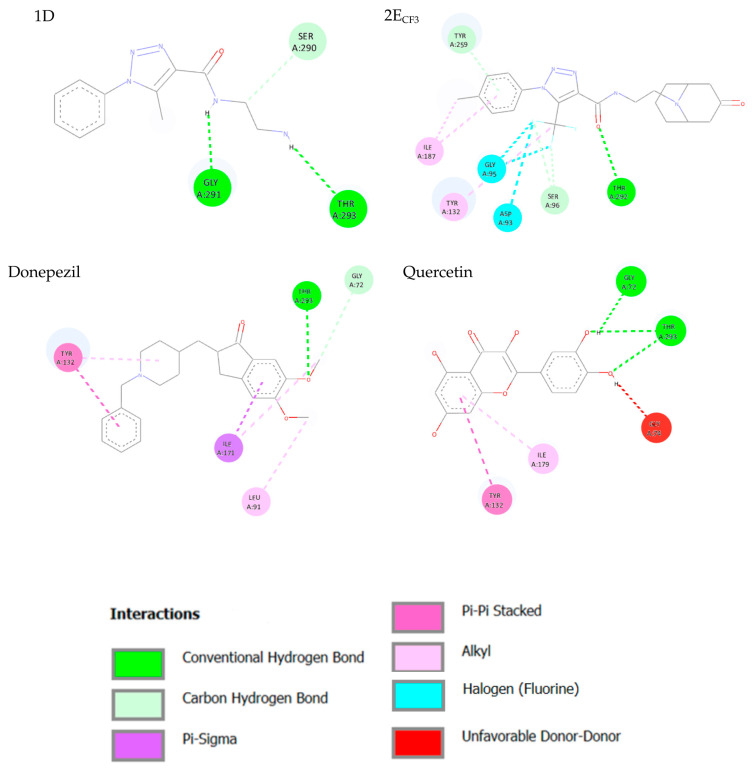
2D diagrams of ligand interactions with BACE1.

Similar interaction diagrams were generated for the complexes with BACE1, as shown in Figure 6.

**1D** is involved in the formation of two hydrogen bonds: between the hydrogen atom of the amide group and Gly-271, and between the amino group and Thr-293. **2E(CF_3_)**, on the other hand, forms only one hydrogen bond with Thr-292 via the oxygen atom of the carbonyl group, but there are also interactions with fluorine atoms via Asp-93 and Gly-95. In addition to hydrophobic and π-σ interactions, Donepezil is involved in the formation of a hydrogen bond between the oxygen atom of the methoxy group and Thr-293. In the case of Quercetin, there are three hydrogen bonds between the hydroxyl groups and Gly-72 and Thr-293; in addition, hydrophobic interactions and unfavorable donor–donor interactions are also observed. In the case of BACE1, Thr-293 appears to be the key residue.

**Figure 7:**
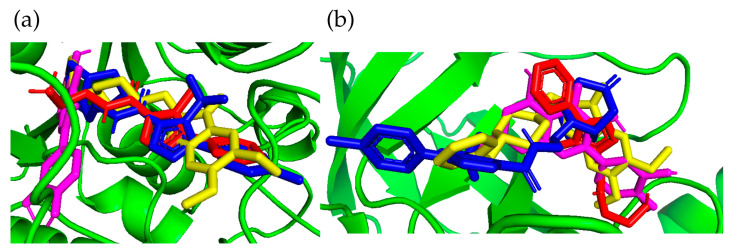
Conformations of ligands 1D (red), 2ECF3 (blue), Donepezil (yellow), and Quercetin (magenta) docked to (**a**) AChE and (**b**) BACE1.

Figure 7 shows the conformations of docked ligands.

Another indication of differences in the mechanisms of action between triazole ligands and reference compounds is the fact that they adopt different conformations in the active site.

Statistical analysis of molecular docking was conducted. From the docking results for AChE, the following conformations of compounds were selected: **1D** with an energy lower than −8 kcal/mol, **2**E**(CF_3_)**, Donepezil with an energy lower than −11 kcal/mol, and Quercetin with an energy lower than −9 kcal/mol. These conformations were divided into positions, which are listed in Table 3:

All ligands except Donepezil interact with the protein in three main ways. Triazole ligands are more flexible than Donepezil. Data on the number of conformations are summarized in Table 4:

In all cases except Quercetin, Position 1 with the lowest median energy occurs most frequently. Statistical parameters for energy values are summarized in Table 5.

The Kruskal–Wallis test was performed to determine differences in the energy distributions of the most common positions, which shows that the energy distributions of triazole ligands differ statistically significantly not only from the reference ligands, but also from each other (with the exception of **2E(CF_3_)** and Donepezil, whose energies do not differ from each other). This fact, together with differences in interactions with the protein, suggests that the mechanism of action of triazoles varies from that of Donepezil and Quercetin.

A similar analysis was performed for complexes with BACE1—**1D** conformations with energy less than −6.5 kcal/mol were taken into account, as well as those with energy less than −8 kcal/mol for other ligands. The positions are presented in Table 6:

As with AChE, Donepezil is the least flexible ligand—it binds to the protein in only one way, while the rest of the compounds form three main couplings. Data on the number of conformations are summarized in Table 7.

In the case of **1D**, the position with the lowest median energy occurs most frequently, while for **2E(CF_3_)**, Position 2 occurs most often. For Quercetin, positions 1 and 2 occur with equal regularity. Statistical parameters for energy values are summarized in Table 8.

As in the case of AChE, the Kruskal–Wallis test was performed. It shows that in most cases, there are statistically significant differences. Distributions that do not differ from each other occur in the comparison of the energy of Position 1 **2E(CF_3_)**, with Position 2 **2E(CF_3_)**, Donepezil, and Position 1 Quercetin. This, together with information on interactions with the enzyme, suggests that **1D** has a different mechanism of action compared to the reference ligands. In turn, the mechanism of action of 2E**(CF_3_)** may be similar to Donepezil and Quercetin.

**Figure 8:**
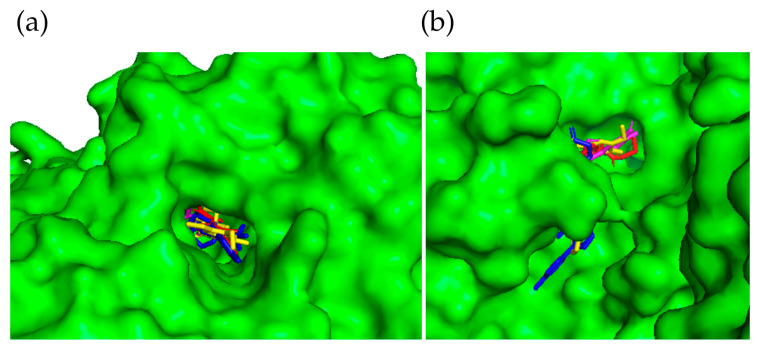
Conformations of ligands 1D (red), **2E(CF_3_)** (blue), Donepezil (yellow), and Quercetin (magenta) docked to (**a**) AChE and (**b**) BACE1.

In order to determine the type of inhibition, the binding site of ligands to the protein was analyzed—Figure 8.

In the case of both AChE and BACE1, all analyzed ligands are located in the active site of the enzyme, suggesting a competitive inhibition mechanism.

### 2.4. Molecular Dynamics

**Figure 9:**
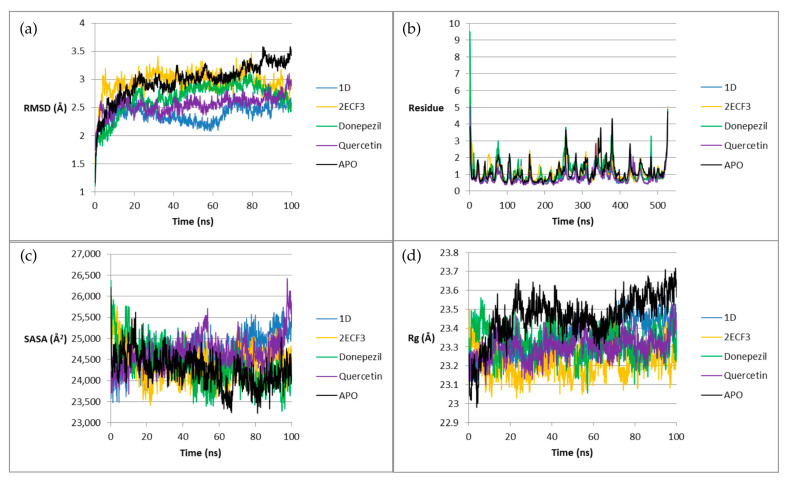
Relationship graphs: (**a**) RMSD versus time; (**b**) RMSF versus residue number; (**c**) SASA versus time; (**d**) Rg versus time for compounds **1D**, **2E(CF_3_)**, Donepezil, and Quercetin docked to AChE, and for the apoenzyme.

Molecular dynamics (MD) simulations lasting 100 ns were performed and examined in terms of the following parameters: root mean square deviation (RMSD), root mean square fluctuation (RMSF), solvent accessible surface area (SASA), and radius of gyration (Rg). Low values of the tested coefficients induce stability of a given system. Comparison of the values of triazole ligands with the apoenzyme and reference ligands allows us to determine whether a given compound has a stabilizing or destabilizing effect on the enzyme. Enzyme stabilization, in turn, directly translates into inhibition efficacy—in practice, this means that compounds whose complexes exhibit low RMSD, RMSF, SASA, and Rg values will potentially be more therapeutically effective. The results for AChE are presented in Figure 9.

The RMSD values for **1D** are the lowest among the studied systems for most of the simulation, with the exception of the beginning and end of the simulation. In contrast, **2E_CF3_** shows a relatively high RMSD throughout the simulation, but it is comparable to the apoenzyme, and in the last 20 ns, it is lower.

The RMSF versus residuum number graph is characterized by peaks. In this case, they appear mainly for Donepezil (e.g., Leu-76, Gly-256, Phe-483) and apoenzyme (e.g., Gly-256, Asp-349, Val-379, Gln-527). The values for **1D** in the case of peaks are low, and for **2E_CF3_**, they are higher, but still lower than for apoenzyme and Donepezil.

The SASA values for **1D** at the beginning of the simulation are the lowest, but then they increase and become comparable to the rest of the tested systems until approx. 70 ns, when they become the highest. In contrast, SASA for **2E(CF_3_)** is high at the very beginning, but quickly decreases and reaches the lowest values compared to the rest of the systems. This is the case until approx. 45 ns of simulation; after this time, the values become comparable to the rest of the complexes.

The Rg parameter values for **1D** are similar to those of the reference ligands for most of the simulation. Only after approx. 70 ns does Rg increase, but it still remains lower than the value for the apoenzyme. In turn, the values for **2E(CF_3_)** are the lowest compared to the rest of the systems for most of the simulation.

The relatively low values of the analyzed parameters indicate the stabilizing effect of triazole ligands on AChE, which demonstrates their potential for use in the treatment of AD.

**Figure 10:**
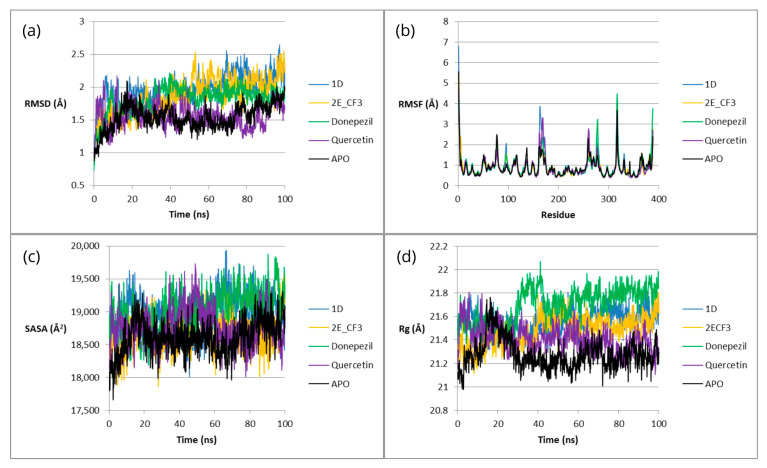
Relationship graphs: (**a**) RMSD versus time; (**b**) RMSF versus residue number; (**c**) SASA versus time; and (**d**) Rg versus time for compounds **1D**, **2E_CF3_**, Donepezil, Quercetin docked to BACE1, and for the apoenzyme.

A similar analysis was performed for complexes with BACE1—Figure 10.

The RMSD values for **1D** from the beginning to approximately halfway through the simulation are similar to those of the reference ligands, after which they increase and become the highest compared to the other systems at the end of the simulation. A similar trend is observed for **2E(CF_3_)**—in the first half of the simulation, the values are similar to those of the other complexes, and in the second half, they become higher than those of the other complexes.

As in the case of AChE, the graph of RMSF versus residuum number contains peaks. For **1D**, a small-peak Ser-96 and a large-peak Ile-163 are observed. For **2E(CF_3_)**, no clear peaks are observed. In contrast, the RMSF of Donepezil peaks in three cases: Cys-278, Lys-317, and Ser-388. In the case of Quercetin, there are two peaks: Lys-168 and Glu-261. However, for residuum Glu-78, the peak occurs for the apoenzyme.

The SASA values for **1D** throughout the simulation are relatively high, but comparable to Donepezil. In contrast, the values for **2E(CF_3_)** are among the lowest and are similar to those for the apoenzyme, becoming the lowest at the end of the simulation.

Rg for **1D** at the beginning of the simulation is relatively high and comparable to Donepezil, then it decreases and is similar to Quercetin, and from about halfway through the simulation, it remains between Donepezil and Quercetin. In turn, the values for **2E(CF_3_)** are low at the beginning, and then they gradually increase and stabilize around the middle of the simulation—at that point, they are comparable to **1D**.

In the case of BACE1, the stabilizing effect of compounds **1D** and **2E(CF_3_)** is also observed, but it is less pronounced than for AChE. These compounds may serve as multi-target agents in the treatment of AD, although they will be more effective against AChE.

**Figure 11:**
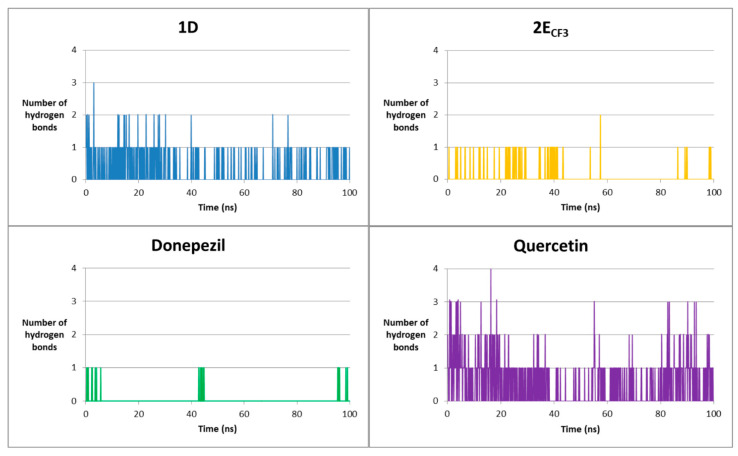
Graphs showing the dependence of the number of hydrogen bonds on the duration of the simulation for AChE complexes.

In addition to the simulation parameters, the time evolution of the hydrogen bond system was also tracked. The results for the AChE complexes are presented in Table 9 and Figure 11.

Throughout most of the simulations, ligand **1D** typically forms a single hydrogen bond. Four residues are involved in this bond, most commonly Tyr-337, which was already observed during the docking stage. In contrast, **2E(CF_3_)** forms hydrogen bonds less frequently and only with one residue, Tyr-124, which was also evident in the docking analysis. This residue is also key in the interactions of other compounds with AChE [54]. Donepezil is practically not involved in the formation of hydrogen bonds. Quercetin, on the other hand, which showed the highest number of hydrogen bonds at the docking stage, also maintains this trend during the simulation—there was at least one hydrogen bond at virtually every stage. However, the only bond that coincides with the docking results is that with Hsd-447. Triazole ligands engage in interactions with residues other than reference ligands, suggesting a distinct mechanism of action.

**Figure 12:**
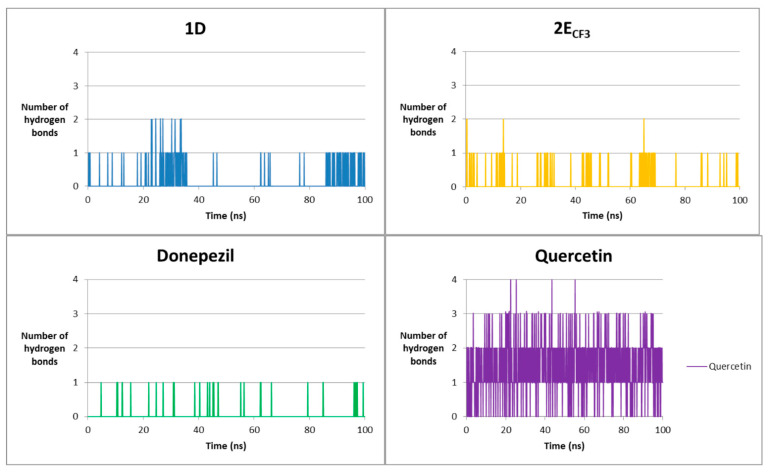
Graphs showing the dependence of the number of hydrogen bonds on the duration of the simulation for BACE1 complexes.

A similar analysis was performed for complexes with BACE1—Table 10 and Figure 12.

The **1D** and **2E(CF_3_)** compounds form hydrogen bonds with similar frequency—approximately half of the simulation time, a single bond occurs most frequently. **1D** most often interacts with Thr-293, which was already evident at the docking stage. In the case of **2E_CF3_**, the main residue is Thr-133. During the simulation, no bond with Thr-292 present at the docking stage was observed. Donepezil forms the fewest hydrogen bonds and does so the least frequently. In this case, no bond with the residue with which a bond was formed at the docking stage was observed either. Quercetin is very often involved in the formation of hydrogen bonds. It does so mainly with Asp-93, Val,-92, and Gln-134, but in this case, the bond arrangement also does not coincide with that obtained by docking. As in the case of AChE, a different mechanism of action of triazoles is possible due to differences in the hydrogen bonds formed. These results are also confirmed by other computational studies, indicating residues Asp-93 and Thr-293 as key [55].

**Figure 13:**
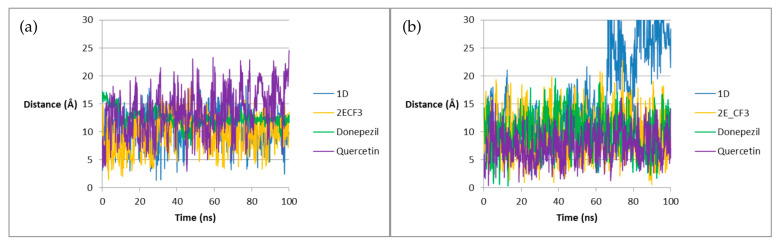
Graphs showing the relationship of the distance between ligands and the residues with which they most often form hydrogen bonds for complexes with (**a**) AChE and (**b**) BACE1.

To gain additional insight into the stability of the complexes, the distance between the ligand and the residue with which hydrogen bonds are most commonly formed was examined—Figure 13.

In the case of AChE, the distance values for **1D** are comparable to Donepezil, and they are sometimes even the lowest among the tested systems. In turn, the values for **2E(CF_3_)** are the lowest for most of the simulations. In the case of BACE1, the values for **1D** are similar to the other systems, except for the last 30 ns of the simulation, when they become the highest. The distance for **2E(CF_3_)** changes dynamically—sometimes the values are the lowest, and sometimes the highest, but the overall trend is similar to Donepezil. This analysis confirms the conclusions about the stabilizing effect of triazole ligands on the enzyme, which is more visible in the case of AChE.

**Figure 14:**
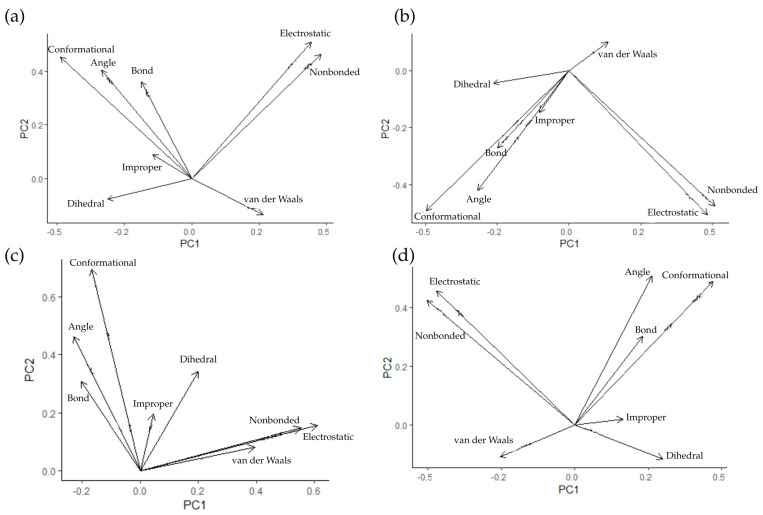
Biplots illustrating the relationships between energy types throughout the simulation for (**a**) **1D**, (**b**) **2E_CF3_**, (**c**) Donepezil, and (**d**) Quercetin docked to AChE.

The relationships between the types of energy in the systems during simulation were also examined. Emphasis was placed on the energy resulting from bonds, angles, dihedrals, and impropers, as well as electrostatic and van der Waals interactions, and conformational and nonbonded energies. For this purpose, principal component analysis (PCA) was performed, and the results for AChE systems are presented in Figure 14.

**Figure 15:**
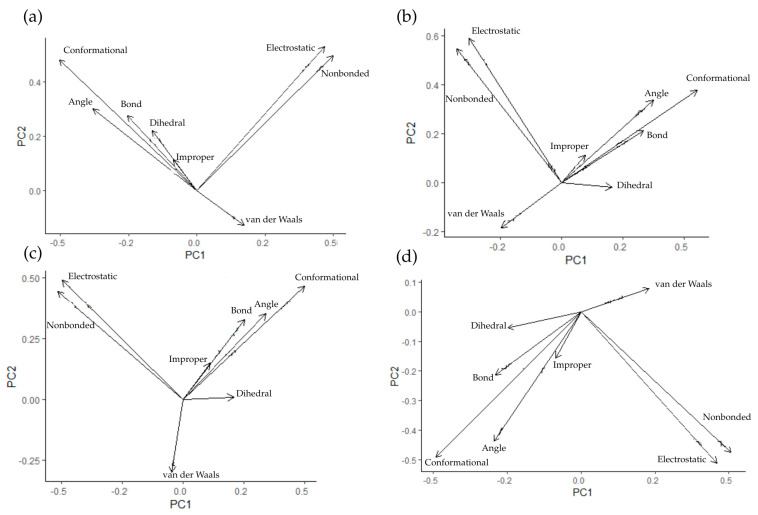
Biplots illustrating the relationships between energy types throughout the simulation for (**a**) **1D**, (**b**) **2E_CF3_**, (**c**) Donepezil, and (**d**) Quercetin docked to BACE1.

For all bond, angle, improper, and conformational systems, the energies are positively correlated with each other. A similar correlation is observed for electrostatic and nonbonded energies. The key difference in this case is the relationship between dihedral energies and van der Waals interactions. They correlate negatively with other energies in all cases except Donepezil, which is another indication of differences in the effect of this ligand on the protein. A similar analysis was performed for systems with BACE1—Figure 15.

As in the case of AChE systems, there are strong positive correlations between bond, angle, improper, and conformational energies, as well as between electrostatic and nonbonded energies. Once again, the key difference lies in the relationship between dihedral and van der Waals energies and other vectors. These two types of interactions are negatively correlated for all compounds except Donepezil, where a negligible relationship between them was observed. As in the case of AChE, a different mechanism of action can be inferred for Donepezil.

### 2.5. QSAR Modeling

In order to estimate the pharmacokinetic parameters of the tested compounds, an ADEMT (absorption, distribution, metabolism, excretion, and toxicity) analysis based on the QSAR (quantitative structure–activity relationship) model was performed. The results are summarized in Table 11:

Unlike reference ligands, triazole ligands exhibit high toxicity to rats.

**1D**, like Donepezil and Quercetin, has low carcinogenicity, unlike **2E_CF3_**. **1D** is less toxic to the liver than Donepezil, while **2E(CF_3_)** is more toxic. Triazoles, on the other hand, are less toxic to the skin than Quercetin. Inhalation toxicity for **1D** is high and comparable to Quercetin, while for **2E(CF_3_)**, it is lower, but Donepezil has the lowest toxicity. AMES is highest for triazoles. In turn, all tested compounds show low toxicity to the eyes.

**1D** and Quercetin satisfy all four principles. **2E(CF_3_)** does not satisfy the GSK principle. Donepezil, on the other hand, satisfies only the Lipinski and Golden Triangle principles.

All analyzed ligands are characterized by low HIA. In turn, the F_20%_ index is high only for Quercetin. Triazole ligands, unlike reference ligands, are characterized by a low degree of binding to plasma proteins. In addition, all compounds except Quercetin have a high ability to cross the blood–brain barrier. **1D** and Quercetin have a low probability of acting as PGH inhibitors, while the probability of acting as substrates is high only for Donepezil.

The high BBB index is an advantage of triazoles, which is particularly important in the case of drugs used in AD therapy. Additional advantages include a low PPB index, compliance with all or most principles, and low toxicity of compound **1D**. **2E(CF_3_)** exhibits higher toxicity. The disadvantages of triazoles include, for example, their high toxicity to rats or low F_20%_.

## 3. Materials and Methods

### 3.1. Chemistry—General Information

Melting points were determined using a Mettler Toledo MP70 Melting Point System (Mettler-Toledo International Inc., Greifensee, Switzerland). ^1^H NMR and ^13^C NMR spectra were recorded on a Bruker Avance II 400 (400 MHz) or Bruker Ascend (500 MHz) spectrometer (Bruker BioSpin GmbH & Co. KG, Ettlingen, Germany) using 5 mm probes. Chemical shifts δ are reported in parts per million downfield from tetramethylsilane, referenced to residual nondeuterated solvent signals of the NMR solvent used (in deuterated dimethyl sulfoxide, DMSO-d_6_ (2.50 ppm, ^1^H NMR; 39.52 ppm, ^13^C NMR) and in deuterated chloroform, CDCl_3_ (7.26 ppm, ^1^H NMR; 77.0 ppm, ^13^C NMR). Data processing, including Fourier transformation, baseline correction, phasing, peak peaking, and integrations, was performed using MestReNova software v.6.0.2. NMR spectra of amines **1E-6E**, **1E(CF3)**, and **2E(CF3)** hydrochlorides in DMSO-d_6_ often showed separate signals for both N-invertomers. The signal separation in the NMR spectra results from a decreased rate of inversion on the nitrogen atom [51]. TLCs were performed on precoated normal-phase, silica gel-covered, aluminum sheets (Silica gel 60 F254, Merck, Darmstadt, Germany). High Resolution Mass Spectra (HRMS) were recorded on a Bruker microTOF-QIII (Bruker, Daltonics GmbH & Co. KG, Bremen, Germany) equipped with electrospray ionization mode and a time-of-flight detector (TOF).

### 3.2. Synthesis and Spectroscopic Analysis of Compounds

#### 3.2.1. Synthesis of Aromatic Azides (**1B**–**6B**)

General Procedure 1 (**GP1**):

An aqueous solution of sodium nitrite (22.77 g, 0.33 mol, in 75 mL of H_2_O, 1.1 equiv.) was added dropwise to a 0 °C-cooled solution of amine (**1A–6A**, 0.30 mol, 1.0 equiv.) in hydrochloric acid (3 M, 500 mL, 1.5 mol, 5.0 equiv.) over 30 min under vigorous stirring. The mixture was stirred for an additional 30 min at 0–5 °C. Then, an aqueous solution of sodium azide (21.45 g, 0.33 mol, in 75 mL of H_2_O, 1.1 equiv.) was added dropwise over 30 min under vigorous stirring. Intense stirring continued for an additional 30 min. Then, the mixture was extracted with AcOEt (3 × 100 mL). The organic extracts were washed with brine (100 mL), treated with anhydrous Na_2_SO_4_, and filtered. The solvent was removed under reduced pressure to afford the product as an oil. In all cases, the crude, unpurified products (**1B**–**6B**) were used directly in the next synthetic step.

**Azidobenzene** [56] (**1B**) was synthesized from phenylamine (**1A**) according to the General Procedure 1 and was obtained as a light yellow oil (34.70 g, 97%), R_f_ = 0.65 (heptane). **^1^H NMR** (500 MHz, CDCl_3_), δ [ppm]: 7.44—7.39 (m, 2H), 7.28–7.19 (m, 1H), and 7.13–7.07 (m, 2H).

**1-Azido-4-methylbenzene** [56] (**2B**) was synthesized from 4-methylaniline (**2A**) according to the General Procedure 1 and was obtained as a light yellow oil (38.42 g, 96%), R_f_ = 0.62 (heptane). **^1^H NMR** (500 MHz, CDCl_3_), δ [ppm]: 7.21 (d, *J* = 8.2 Hz, 2H), 6.99 (d, *J* = 8.4 Hz, 2H), and 2.40 (s, 3H).

**1-Azido-4-chlorobenzene** [57,58] (**3B**) was synthesized from 4-chloroaniline (**3A**) according to the General Procedure 1 and was obtained as a light yellow-brown oil (43.86 g, 95%), R_f_ = 0.68 (AcOEt/hexane: 3/7, *v*/*v*). **^1^H NMR** (500 MHz, CDCl_3_), δ [ppm]: 7.29 (d, *J* = 6.0 Hz, 2H), 6.92 (d, *J* = 8.8 Hz, 2H).

**1-Azido-2,4-dichlorobenzene** [57] (**4B**) was synthesized from 2,4-dichloroaniline (**4A**) according to the General Procedure 1, but with a modified isolation step. The post-reaction suspension was vacuum-filtered through a sintered glass funnel, and the solid residue was thoroughly washed with distilled water. The crude product was then dried under reduced pressure (50 mbar, RT, 12 h) to afford a brown solid (53.10 g, 94%), R_f_ = 0.63 (AcOEt/hexane: 3/7, *v*/*v*). **^1^H NMR** (500 MHz, CDCl_3_), δ [ppm]: 7.34 (d, *J* = 2.3 Hz, 1H), 7.23 (dd, *J* = 8.6, 2.3 Hz, 1H), and 7.05 (d, *J* = 8.6 Hz, 1H).

**1-Azido-3-chlorobenzene** [58] (**5B**) was synthesized from 3-chloroaniline (**5A**) according to the General Procedure 1 and was obtained as a light yellow-brown oil (43.35 g, 94%), R_f_ = 0.67 (AcOEt/hexane: 3/7, *v*/*v*). **^1^H NMR** (500 MHz, CDCl_3_), δ [ppm]: 7.03 (t, *J* = 8.1 Hz, 1H), 6.89 (ddd, *J* = 8.0, 1.9, 0.9 Hz, 1H), 6.78 (t, *J* = 2.1 Hz, 1H), and 6.68 (ddd, *J* = 8.1, 2.2, 0.8 Hz, 1H).

**1-Azido-2-chlorobenzene** [56,58] (**6B**) was synthesized from 2-chloroaniline (**6A**) according to the General Procedure 1 and was obtained as a light yellow-brown oil (44.27 g, 96%), R_f_ = 0.75 (AcOEt/hexane: 3/7, *v*/*v*). **^1^H NMR** (500 MHz, CDCl_3_), δ [ppm]: 7.53 (dd, *J* = 8.0, 1.4 Hz, 1H), 7.46–7.42 (m, 1H), 7.29 (dd, *J* = 8.0, 1.4 Hz, 1H), and 7.26–7.21 (m, 1H).

#### 3.2.2. Synthesis of 1,2,3-Triazole Esters (**1C**–**6C**)

General procedure 2 (**GP2**):

To a vigorously stirred mixture of aromatic azide (**1B–6B**, 0.20 mol, 1.0 equiv.), ethyl acetoacetate (26.03 g, 25.5 mL, 0.20 mol, 1.0 equiv.) and anhydrous powdered potassium carbonate (2.76 g, 0.02 mol, 10 mol%) in DMSO (100 mL) was added DBU (0.30 g, 0.3 mL, 1 mol%), and stirring continued for 30 min. Then, the reaction mixture was transferred to a beaker with ice-cooled distilled water. The formed precipitate was filtered, washed with water, and dried. The dried crude product was purified by crystallization from a suitable solvent (if needed, the EtOH or IPA solution of the crude product could be decolorized using activated carbon).

**Ethyl 5-methyl-1-phenyl-1*H*-1,2,3-triazole-4-carboxylate** [59] (**1C**) was synthesized from azidobenzene (**1B**), according to the General Procedure 2, purified by crystallization from AcOEt/hexane (7/3, *v*/*v*) mixture, and obtained as a white solid (43.48 g, 94%), R_f_ = 0.64 (AcOEt/hexane: 1/2, *v*/*v*). **^1^H NMR** (500 MHz, CDCl_3_), δ [ppm]: 7.57–7.49 (m, 3H), 7.41 (dd, *J* = 7.8, 1.8 Hz, 2H), 4.43 (q, *J* = 7.1 Hz, 2H), 2.55 (s, 3H), and 1.41 (t, *J* = 7.1 Hz, 3H). **^13^C NMR** (126 MHz, CDCl_3_), δ [ppm]: 161.8, 138.9, 136.8, 135.5, 130.1, 129.7, 125.4, 61.1, 14.4, and 10.0.

**Ethyl 5-methyl-1-(*p*-tolyl)-1*H*-1,2,3-triazole-4-carboxylate** [47] (**2C**) was synthesized from 1-azido-4-methylbenzene (**2B**), according to the General Procedure 2, purified by crystallization from AcOEt/hexane (7/3, *v*/*v*) mixture, and obtained as a white solid (45.13 g, 92%), R_f_ = 0.69 (AcOEt/hexane: 1/2, *v*/*v*). **^1^H NMR** (500 MHz, CDCl_3_), δ [ppm]: 7.33 (d, *J* = 8.2 Hz, 2H), 7.29 (d, *J* = 8.5 Hz, 2H), 4.43 (q, *J* = 7.1 Hz, 2H), 2.54 (s, 3H), 2.42 (s, 3H), and 1.41 (t, *J* = 7.1 Hz, 3H). **^13^C NMR** (126 MHz, CDCl_3_), δ [ppm]: 161.9, 140.4, 138.9, 136.7, 133.0, 130.3, 125.2, 61.1, 21.3, 14.5, and 10.0.

**Ethyl 1-(4-chlorophenyl)-5-methyl-1*H*-1,2,3-triazole-4-carboxylate** [47] (**3C**) was synthesized from 1-azido-4-chlorobenzene (**3B**), according to the General Procedure 2, purified by crystallization from AcOEt/hexane (7/3, *v*/*v*) mixture, and obtained as a white solid (48.94 g, 92%), R_f_ = 0.67 (AcOEt/hexane: 1/2, *v*/*v*). **^1^H NMR** (500 MHz, DMSO-d_6_), δ [ppm]: 7.75–7.71 (m, 2H), 7.70–7.67 (m, 2H), 4.35 (q, *J* = 7.1 Hz, 2H), 2.51 (s, 3H), and 1.33 (t, *J* = 7.1 Hz, 3H). **^13^C NMR** (126 MHz, DMSO-d_6_), δ [ppm]: 161.0, 139.5, 135.9, 134.8, 134.0, 129.8, 127.3, 60.5, 14.2, and 9.7.

**Ethyl 1-(2,4-dichlorophenyl)-5-methyl-1*H*-1,2,3-triazole-4-carboxylate** [60] (**4C**) was synthesized from 1-azido-2,4-dichlorobenzene (**4B**), according to the General Procedure 2, purified by crystallization from AcOEt/hexane (7/3, *v*/*v*) mixture, and obtained as a white solid (54.63 g, 91%), R_f_ = 0.76 (AcOEt/hexane: 1/2, *v*/*v*). **^1^H NMR** (500 MHz, CDCl_3_), δ [pm]: 7.59 (d, *J* = 2.2 Hz, 1H), 7.44 (dd, *J* = 8.5, 2.2 Hz, 1H), 7.36 (d, *J* = 8.5 Hz, 1H), 4.40 (q, *J* = 7.1 Hz, 2H), 2.40 (s, 3H), and 1.39 (t, *J* = 7.1 Hz, 3H). **^13^C NMR** (126 MHz, CDCl_3_), δ [ppm]: 161.5, 140.6, 137.8, 136.3, 132.7, 131.7, 130.6, 130.1, 128.5, 61.2, 14.4, and 9.4.

**Ethyl 1-(3-chlorophenyl)-5-methyl-1*H*-1,2,3-triazole-4-carboxylate** [61] (**5C**) was synthesized from 1-azido-3-chlorobenzene (**5B**), according to the General Procedure 2, purified by crystallization from AcOEt/hexane (7/3, *v*/*v*) mixture, and obtained as a white solid (50.06 g, 94%), R_f_ = 0.67 (AcOEt/hexane: 1/2, *v*/*v*). **^1^H NMR** (500 MHz, CDCl_3_), δ [ppm]: 7.47 (dd, *J* = 3.9, 1.3 Hz, 2H), 7.45–7.42 (m, 1H), 7.33–7.29 (m, 1H), 4.38 (q, *J* = 7.1 Hz, 2H), 2.54 (s, 3H), and 1.37 (t, *J* = 7.1 Hz, 3H). **^13^C NMR** (126 MHz, CDCl_3_), δ [ppm]: 161.5, 138.8, 136.8, 136.3, 135.4, 130.7, 130.2, 125.6, 123.4, 61.1, 14.3, and 10.0.

**Ethyl 1-(2-chlorophenyl)-5-methyl-1*H*-1,2,3-triazole-4-carboxylate** [62] (**6C**) was synthesized from 1-azido-2-chlorobenzene (**6B**), according to the General Procedure 2, purified by crystallization from AcOEt/hexane (7/3, *v*/*v*) mixture, and obtained as a white solid (49.53 g, 93%), R_f_ = 0.60 (AcOEt/hexane: 1/2, *v*/*v*). **^1^H NMR** (500 MHz, CDCl_3_), δ [ppm]: 7.43 (d, *J* = 7.9 Hz, 1H), 7.39 (td, *J* = 7.7, 1.4 Hz, 1H), 7.33 (dd, *J* = 11.1, 4.1 Hz, 1H), 7.28 (dd, *J* = 7.7, 1.2 Hz, 1H), 4.27 (q, *J* = 7.1 Hz, 2H), 2.27 (s, 3H), and 1.25 (t, *J* = 7.1 Hz, 3H). **^13^C NMR** (126 MHz, CDCl_3_), δ [ppm]: 161.2, 140.2, 135.8, 132.6, 132.0, 131.2, 130.3, 128.9, 127.9, 60.7, 14.0, and 9.1.

#### 3.2.3. Synthesis of 1,2,3-Triazole Esters [**1C(CF_3_)**–**2C(CF_3_)**]

General procedure 3 (**GP3**):

To a vigorously stirred mixture of aromatic azide (**1B–2B**, 50.0 mmol, 1.0 equiv.), ethyl 4,4,4-trifloroacetoacetate (9.21 g, 7.3 mL, 50.0 mmol, 1.0 equiv.) and anhydrous powdered potassium carbonate (0.70 g, 5.0 mmol, 10 mol%) in DMSO (30 mL) was added DBU (0.10 g, 0.1 mL, 0.7 mmol, 1 mol%). Stirring continued for 2 h at 120 °C under argon. After cooling, the reaction mixture was poured into a separatory funnel, diluted with water (200 mL), and extracted with AcOEt (3 × 100 mL). The organic extracts were washed with brine (100 mL), treated with anhydrous Na_2_SO_4_, filtered, and concentrated under reduced pressure. The crude product was purified by flash chromatography on silica gel using AcOEt/hexane (1/9, *v*/*v*) as the eluent.

**Ethyl 1-phenyl-5-(trifluoromethyl)-1*H*-1,2,3-triazole-4-carboxylate** [63] [**1C(CF_3_)**] was synthesized from azidobenzene (**1B**), according to the General Procedure 3, and obtained as a yellow oil (12.42 g, 87%), R_f_ = 0.79 (AcOEt/hexane: 1/2, *v*/*v*). **^1^H NMR** (500 MHz, DMSO-d_6_), δ [ppm]: 7.71–7.63 (m, 5H), 4.42 (q, *J* = 7.1 Hz, 2H), and 1.33 (t, *J* = 7.1 Hz, 3H). **^13^C NMR** (126 MHz, DMSO-d_6_), δ [ppm]: 158.7, 138.7, 135.4, 131.3, 129.5 129.2 (dd, *J* = 48.7, 34.4 Hz), 126.2, 122.0 115.3 (m), 61.8, and 13.6. **^19^F NMR** (471 MHz, DMSO-d_6_), δ [ppm]: −55.5.

**Ethyl 1-(*p*-tolyl)-5-(trifluoromethyl)-1*H*-1,2,3-triazole-4-carboxylate** [63] [**2C(CF_3_)**] was synthesized from 1-azido-4-methylbenzene (**2B**), according to the General Procedure 3, and obtained as a yellow oil (13.51 g, 90%), R_f_ = 0.82 (AcOEt/hexane: 1/2, *v*/*v*). **^1^H NMR** (500 MHz, DMSO-d_6_), δ [ppm]: 7.55 (d, *J* = 8.3 Hz, 2H), 7.44 (d, *J* = 8.2 Hz, 2H), 4.41 (q, *J* = 7.1 Hz, 2H), 2.42 (s, 3H), and 1.33 (t, *J* = 7.1 Hz, 3H). **^13^C NMR** (126 MHz, DMSO-d_6_), δ [ppm]: 158.7, 141.4, 138.5, 132.8, 129.9, 129.1 (q, *J* = 41.4 Hz), 126.0, 118.7 (q, *J* = 270.7 Hz), 61.9, 20.7, and 13.8. **^19^F NMR** (471 MHz, DMSO-d_6_), δ [ppm]: −55.3.

#### 3.2.4. Synthesis of 1,2,3-Triazole Amides Hydrochlorides [**1D**–**6D**, **1D(CF_3_)** and **2D(CF_3_)**]

General procedure 4 (**GP4**):

A stirred solution of ester [**1C–6C**, **1C(CF_3_)** or **2C(CF_3_)**, 0.050 mol, 1.0 equiv.] in 1,2-ethylenediamine (15.03 g, 16.73 mL, 5 equiv.) was heated at reflux for 4 h under argon, then allowed to cool to room temperature. The excess 1,2-ethylenediamine was removed under vacuum (50 mbar, 95 °C), and the residue was further dried under high vacuum at 80 °C for 2 h to remove residual 1,2-ethylenediamine. The resulting free amines were purified either by crystallization from methanol or by column chromatography on silica gel using Et_3_N/MeOH/DCM (0.5/10/89.5, *v*/*v*/*v*) as the eluent. The amine hydrochloride was obtained by treating the free amine with anhydrous methanol HCl solution, prepared from cooled 100 mL of methanol premixed with acetyl chloride (7.85 g, 7.1 mL, 2 equiv.), until dissolution, followed by removal of volatiles under reduced pressure (150 mbar, 60 °C). Drying under high vacuum and crystallization from ethyl acetate (100 mL)/ethanol mixed solvent at 2 °C, followed by washing with ethyl acetate and high vacuum drying, provided the pure hydrochloride salt.

***N*-(2-aminoethyl)-5-methyl-1-phenyl-1*H*-1,2,3-triazole-4-carboxamide hydrochloride** (**1D**) was synthesized from ethyl 5-methyl-1-phenyl-1*H*-1,2,3-triazole-4-carboxylate (**1C**), according to the General Procedure 4, and obtained as a white solid (12.54 g, 89%); mp: 244–245 °C, R_f_ = 0.16 (methanol) determined for the free amine. **^1^H NMR** (500 MHz, DMSO-d_6_), δ [ppm]: 8.79 (t, *J* = 5.8 Hz, 1H), 8.27 (br s, 3H), 7.65–7.60 (m, 5H), 3.57 (q, *J* = 6.3 Hz, 2H), 2.98 (t, *J* = 6.4 Hz, 2H), and 2.53 (s, 3H). **^13^C NMR** (126 MHz, DMSO-d_6_), δ [ppm]: 161.3, 138.0, 136.8, 135.3, 130.0, 129.7, 125.3, 38.5, 36.2, and 9.3. **HRMS** (ESI): calcd. for C_12_H_16_N_5_O^+^ [M+H]^+^ 246.1349; found: 246.1362.

***N*-(2-aminoethyl)-5-methyl-1-(*p*-tolyl)-1*H*-1,2,3-triazole-4-carboxamide hydrochloride** (**2D**) was synthesized from ethyl 5-methyl-1-(*p*-tolyl)-1*H*-1,2,3-triazole-4-carboxylate (**2C**) according to the General Procedure 4 and obtained as a white solid (12.72 g, 86%); mp: 254–255 °C and R_f_ = 0.14 (methanol), determined for the free amine. **^1^H NMR** (500 MHz, DMSO-d_6_), δ [ppm]: 8.77 (t, *J* = 5.8 Hz, 1H), 8.12 (br s, 3H), 7.49 (d, *J* = 8.3 Hz, 2H), 7.44 (d, *J* = 8.3 Hz, 2H), 3.56 (q, *J* = 6.3 Hz, 2H), 2.98 (t, *J* = 6.4 Hz, 2H), 2.51 (s, 3H), and 2.42 (s, 3H). **^13^C NMR** (126 MHz, DMSO-d_6_), δ [ppm]: 161.4, 139.8, 137.9, 136.7, 132.8, 130.1, 125.1, 38.6, 36.2, 20.7, and 9.3. **HRMS** (ESI): calcd. for C_13_H_18_N_5_O^+^ [M+H]^+^ 260.1506; found: 260.1506.

***N*-(2-aminoethyl)-1-(4-chlorophenyl)-5-methyl-1*H*-1,2,3-triazole-4-carboxamide hydrochloride** (**3D**) was synthesized from ethyl 1-(4-chlorophenyl)-5-methyl-1*H*-1,2,3-triazole-4-carboxylate (**3C**), according to the General Procedure 4, and obtained as a white solid (14.7 g, 93%); mp: 245–246 °C and R_f_ = 0.14 (methanol), determined for the free amine. **^1^H NMR** (500 MHz, DMSO-d_6_), δ [ppm]: 8.80 (t, *J* = 5.9 Hz, 1H), 8.26 (br s, 3H), 7.71 (dd, *J* = 6.7, 4.3 Hz, 2H), 7.70–7.66 (m, 2H), 3.57 (q, *J* = 6.3 Hz, 2H), 2.98 (t, *J* = 6.4 Hz, 2H), and 2.54 (s, 3H). **^13^C NMR** (126 MHz, DMSO-d_6_), δ [ppm]: 161.2, 138.1, 137.0, 134.7, 134.1, 129.8, 127.2, 38.5, 36.2, and 9.3. **HRMS** (ESI): calcd. for C_12_H_15_ClN_5_O^+^ [M+H]^+^ 280.0960; found: 280.0956.

***N*-(2-aminoethyl)-1-(2,4-dichlorophenyl)-5-methyl-1*H*-1,2,3-triazole-4-carboxamide hydrochloride** (**4D**) was synthesized from ethyl 1-(2,4-dichlorophenyl)-5-methyl-1*H*-1,2,3-triazole-4-carboxylate (**4C**), according to the General Procedure 4, and obtained as a white solid (15.95 g, 91%); mp: 256–257 °C and R_f_ = 0.47 (methanol), determined for the free amine. **^1^H NMR** (500 MHz, DMSO-d_6_), δ [ppm]: 8.84 (t, *J* = 5.9 Hz, 1H), 8.21 (br s, 3H), 8.06 (d, *J* = 2.1 Hz, 1H), 7.80–7.73 (m, 2H), 3.57 (q, *J* = 6.2 Hz, 2H), 2.99 (t, *J* = 6.0 Hz, 2H), and 2.37 (s, 3H). **^13^C NMR** (126 MHz, DMSO-d_6_), δ [ppm]: 161.0, 138.4, 137.6, 136.6, 131.8, 131.5, 131.0, 130.2, 129.0, 38.5, 36.3, and 8.6. **HRMS** (ESI): calcd. for C_12_H_14_Cl_2_N_5_O^+^ [M+H]^+^ 314.0570; found: 314.0574.

***N*-(2-aminoethyl)-1-(3-chlorophenyl)-5-methyl-1*H*-1,2,3-triazole-4-carboxamide hydrochloride** (**5D**) was synthesized from ethyl 1-(3-chlorophenyl)-5-methyl-1*H*-1,2,3-triazole-4-carboxylate (**5C**), according to the General Procedure 4, and obtained as a white solid (14.54 g, 92%); mp: 233–234 °C and R_f_ = 0.21 (methanol), determined for the free amine. **^1^H NMR** (500 MHz, DMSO-d_6_), δ [ppm]: 8.84–7.78 (m, 1H), 8.23 (br s, 3H), 7.81 (s, 1H), 7.73–7.61 (m, 3H), 3.60–3.53 (m, 2H), 2.98 (t, *J* = 5.4 Hz, 2H), and 2.55 (s, 3H). **^13^C NMR** (126 MHz, DMSO-d_6_), δ [ppm]: 161.2, 138.0, 137.1, 136.4, 133.9, 131.4, 130.1, 125.3, 124.3, 38.5, 36.3, and 9.3. **HRMS** (ESI): calcd. for C_12_H_15_ClN_5_O^+^ [M+H]^+^ 280.0960; found: 280.0957.

***N*-(2-aminoethyl)-1-(2-chlorophenyl)-5-methyl-1*H*-1,2,3-triazole-4-carboxamide hydrochloride** (**6D**) was synthesized from ethyl 1-(2-chlorophenyl)-5-methyl-1*H*-1,2,3-triazole-4-carboxylate (**6C**), according to the General Procedure 4, and obtained as a white solid (14.86 g, 94%); mp: 218–219 °C and R_f_ = 0.23 (methanol), determined for the free amine. **^1^H NMR** (500 MHz, DMSO-d_6_), δ [ppm]: 8.83 (t, *J* = 5.9 Hz, 1H), 8.34 (br s, 3H), 7.82–7.79 (m, 1H), 7.73–7.69 (m, 2H), 7.65–7.61 (m, 1H), 3.59 (q, *J* = 6.2 Hz, 2H), 3.04–2.96 (m, 2H), and 2.35 (s, 3H). **^13^C NMR** (126 MHz, DMSO-d_6_), δ [ppm]: 161.1, 138.1, 137.5, 132.8, 132.5, 130.6, 130.4, 129.8, 128.8, 38.5, 36.3, and 8.7. **HRMS** (ESI): calcd. for C_12_H_15_ClN_5_O^+^ [M+H]^+^ 280.0960; found: 280.0961.

***N*-(2-aminoethyl)-1-phenyl-5-(trifluoromethyl)-1*H*-1,2,3-triazole-4-carboxamide hydrochloride** [**1D(CF_3_)**] was synthesized from ethyl 1-phenyl-5-(trifluoromethyl)-1*H*-1,2,3-triazole-4-carboxylate [**1C(CF_3_)**], according to the General Procedure 4, and obtained as a white solid (15.61 g, 93%); mp: 214–215 °C and R_f_ = 0.30 (methanol), determined for the free amine. **^1^H NMR** (500 MHz, DMSO-d_6_), δ [ppm]: 9.20 (t, *J* = 5.7 Hz, 1H), 8.31 (br s, 3H), 7.71–7.65 (m, 5H), 3.61 (q, *J* = 6.4 Hz, 2H), and 3.00 (t, *J* = 6.6 Hz, 2H). **^13^C NMR** (126 MHz, DMSO-d_6_), δ [ppm]: 158.3, 141.9, 135.4, 131.4, 129.6, 127.6 (q), 126.2, 118.9 (q), 38.1, and 36.7. **^19^F NMR** (376 MHz, DMSO-d_6_), δ [ppm]: −54.6. **HRMS** (ESI): calcd. for C_12_H_13_F_3_N_5_O^+^ [M+H]^+^ 300.1067; found: 300.1069.

***N*-(2-aminoethyl)-1-phenyl-5-(trifluoromethyl)-1*H*-1,2,3-triazole-4-carboxamide** [**2D(CF_3_)**] was synthesized from ethyl 1-(*p*-tolyl)-5-(trifluoromethyl)-1*H*-1,2,3-triazole-4-carboxylate [**2C(CF_3_)**], according to the General Procedure 4, and obtained as a white solid (16.09 g, 92%); mp: 105–106 °C and R_f_ = 0.21 (methanol), determined for the free amine. **^1^H NMR** (500 MHz, DMSO-d_6_), δ [ppm]: 9.08 (s, 1H), 7.53 (d, *J* = 8.3 Hz, 2H), 7.45 (d, *J* = 8.2 Hz, 2H), 4.31 (brs, 2H), 3.49 (d, *J* = 4.9 Hz, 2H), 2.91 (t, *J* = 6.4 Hz, 2H), and 2.43 (s, 3H). **^13^C NMR** (126 MHz, DMSO-d_6_), δ [ppm]: 158.3, 142.0, 141.4, 132.9, 130.0, 127.6 (q), 126.0, 119.0 (q), 38.6, 30.7, and 20.8. **^19^F NMR** (376 MHz, DMSO-d_6_), δ [ppm]: −54.7. **HRMS** (ESI): calcd. for C_13_H_15_F_3_N_5_O^+^ [M+H]^+^ 314.1223; found 314.1212.

#### 3.2.5. Synthesis of Aromatic 1,2,3-Triazole Amines Hydrochloride [**1E**–**6E**, **1E(CF3)** and **2E(CF3)**]

General procedure 5 (**GP5**):

A microwave reactor vessel was charged with amide hydrochloride **1D–6D**, **1D(CF_3_)** or **2D(CF_3_)**, 2.0 mmol, 1.0 equiv.], pseudopelletierine methiodide (0.59 g, 2.0 mmol, 1.0 equiv.), potassium carbonate (0.83 g, 6 mmol, 3 equiv.), and an EtOH/H_2_O mixture (20 mL, 1:1, *v*/*v*). The mixture in the open vessel was stirred for 15 min at rt, and then the vessel was sealed and heated in a microwave reactor for 2 h at 100 °C. After cooling to room temperature, the reaction mixture was extracted with AcOEt (3 × 50 mL), the extracts were dried over anhydrous Na_2_SO_4_, filtered, and concentrated under reduced pressure. The crude product was purified by flash column chromatography on silica gel using AcOEt/hexane (10:80, *v*/*v*) as the eluent. The free amine was converted to hydrochloride by dissolution in anhydrous methanol HCl solution, made from cooled methanol (20 mL) and acetyl chloride (0.55 g, 0.5 mL, 3.5 equiv.), followed by removal of volatiles (150-50 mbar, 60 °C) and further drying under high vacuum. Crystallization from mixed solvent, ethyl acetate (100 mL)/anhydrous ethanol at 2 °C, washing with ethyl acetate, and drying under high vacuum gave the pure hydrochloride salt.

**5-Methyl-*N*-(2-(3-oxo-9-azabicyclo [3.3.1]nonan-9-yl)ethyl)-1-phenyl-1*H*-1,2,3-triazole-4-carboxamide hydrochloride** (**1E**) was synthesized from *N*-(2-aminoethyl)-5-methyl-1-phenyl-1*H*-1,2,3-triazole-4-carboxamide hydrochloride (**1D**), according to the General Procedure 5, and obtained as a white solid (0.72 g, 89%); mp: 236–237 °C and R_f_ = 0.9 (methanol/DCM: 1/10, *v*/*v*), determined for the free amine. **^1^H NMR** (400 MHz, DMSO-d_6_), δ [ppm]: 11.94, 11.47 (s, 1H), 8.98–8.90 (m, 1H), 7.65–7.60 (m, 1H), 7.49 (d, *J* = 8.3 Hz, 2H), 7.43 (d, *J* = 8.3 Hz, 2H), 4.13–4.02 (m, 2H), 3.89–3.80 (m, 2H), 3.63–3.54 (m, 2H), 3.48 (dd, *J* = 17.1, 5.8 Hz, 1H), 3.16 (dd, *J* = 18.1, 6.0 Hz, 1H), 2.57–2.52 (m, 1H), 2.49–2.44 (m, 1H), 2.41 (s, 3H), 2.39–2.30 (m, 1H), 2.24–2.06 (m, 1H), 1.83 (d, *J* = 13.7 Hz, 1H), 1.73–1.55 (m, 2H), and 1.40–1.23 (m, 1H). Some signals were split due to the presence of N-invertomers, **^13^C NMR** (101 MHz, DMSO-d_6_), δ [ppm]: 204.9, 204.4, 161.8, 140.3, 138.3, 137.3, 135.7, 133.3, 130.6, 130.2, 125.8, 125.6, 55.3, 55.4, 51.0, 50.1, 43.9, 34.4, 33.9, 28.9, 23.1, 21.2, 14.6, 14.3, 9.8, and 9.77. **HRMS** (ESI): calcd. for C_20_H_26_N_5_O_2_^+^ [M+H]^+^ 368.2081; found: 368.2075.

**5-Methyl-*N*-(2-(3-oxo-9-azabicyclo [3.3.1]nonan-9-yl)ethyl)-1-(*p*-tolyl)-1*H*-1,2,3-triazole-4-carboxamide hydrochloride** (**2E**) was synthesized from *N*-(2-aminoethyl)-5-methyl-1-(*p*-tolyl)-1*H*-1,2,3-triazole-4-carboxamide hydrochloride (**2D**), according to the General Procedure 5, and obtained as a white solid (0.77 g, 92%); mp: 239–240 °C and R_f_ = 0.87 (methanol/DCM: 1/10, *v*/*v*), determined for the free amine. Some signals a split due to invertomer. **^1^H NMR** (400 MHz, DMSO-d_6_), δ [ppm]: 12.04, 11.59 (s, 1H), 9.01–8.83 (m, 1H), 7.49 (d, *J* = 8.3 Hz, 2H), 7.43 (d, *J* = 8.4 Hz, 2H), 4.15–4.97 (m, 2H), 3.94–3.75 (m, 2H), 3.67–3.54 (m, 2H), 3.53–3.44 (m, 1H), 3.21–3.07 (m, 1H), 2.60–2.51 (m, 1H), 2.51 (s, 3H), 2.49–2.44 (m, 1H), 2.41 (s, 3H), 2.40–2.33 (m, 1H), 2.27–2.05 (m, 1H), 1.82 (d, *J* = 6.1 Hz, 1H), 1.63 (dd, *J* = 32.6, 13.1 Hz, 2H), and 1.38–1.25 (m, 1H). Some signals were split due to the presence of N-invertomers, **^13^C NMR** (101 MHz, DMSO-d_6_), δ [ppm]: 204.9, 204.4, 161.7, 140.3, 138.3, 137.2, 133.3, 130.6, 125.6, 55.2, 54.4, 51.1, 50.1, 43.8, 34.4, 33.9, 28.9, 23.2, 21.2, 14.6, 14.3, and 9.8. **HRMS** (ESI): calcd. for C_21_H_28_N_5_O_2_^+^ [M+H]^+^ 382.2238; found: 382.2236.

**1-(4-Chlorophenyl)-5-methyl-*N*-(2-(3-oxo-9-azabicyclo [3.3.1]nonan-9-yl)ethyl)-1*H*-1,2,3-triazole-4-carboxamide hydrochloride** (**3E**) was synthesized from *N*-(2-aminoethyl)-1-(4-chlorophenyl)-5-methyl-1*H*-1,2,3-triazole-4-carboxamide hydrochloride (**3D**), according to the General Procedure 5, and obtained as a white solid (0.77 g, 88%); mp: 238–239 °C and R_f_ = 0.79 (methanol/DCM: 1/10, *v*/*v*), determined for the free amine. **^1^H NMR** (400 MHz, DMSO-d_6_), δ [ppm]: 11.93, 11.45 (s, 1H), 9.00–8.92 (m, 1H), 7.74–7.67 (m, 4H), 4.15–4.02 (m, 2H), 3.90–3.79 (m, 2H), 3.63–3.53 (m, 2H), 3.48 (dd, *J* = 16.5, 4.8 Hz, 1H), 3.16 (dd, *J* = 17.6, 4.9 Hz, 1H), 2.60–2.55 (m, 1H), 2.54 (s, 3H), 2.48–2.41 (m, 1H), 2.40–2.34 (m, 1H), 2.20–2.05 (m, 1H), 1.83 (d, *J* = 13.4 Hz, 1H), 1.73–1.55 (m, 2H), and 1.40–1.23 (m, 1H). Some signals were split due to the presence of N-invertomers, **^13^C NMR** (101 MHz, DMSO-d_6_), δ [ppm]: 204.9, 204.3, 161.6, 138.4, 137.6, 135.1, 134.6, 130.3, 127.7, 55.3, 54.4, 51.0, 50.1, 43.9, 34.4, 33.9, 28.9, 23.1, 14.6, 14.3, and 9.8. **HRMS** (ESI): calcd. for C_20_H_25_ClN_5_O_2_^+^ [M+H]^+^ 402.1691; found: 402.1690.

**1-(2,4-Dichlorophenyl)-5-methyl-*N*-(2-(3-oxo-9-azabicyclo [3.3.1]nonan-9-yl)ethyl)-1*H*-1,2,3-triazole-4-carboxamide hydrochloride** (**4E**) was synthesized from *N*-(2-aminoethyl)-1-(2,4-dichlorophenyl)-5-methyl-1*H*-1,2,3-triazole-4-carboxamide hydrochloride (**4D**), according to the General Procedure 5, and obtained as a white solid (0.80 g, 85%); mp: 248–249 °C and R_f_ = 0.56 (methanol/DCM: 1/10, *v*/*v*), determined for the free amine. **^1^H NMR** (400 MHz, DMSO-d_6_), δ [ppm]: 11.71, 11.17 (s, 1H), 9.00 (t, *J* = 6.3 Hz, 1H), 8.08 (s, 1H), 7.78 (dd, *J* = 11.3, 5.0 Hz, 2H), 4.10 (d, *J* = 22.1 Hz, 2H), 3.90–3.78 (m, 2H), 3.63–3.53 (m, 2H), 3.43 (dd, *J* = 14.1, 7.0 Hz, 1H), 3.19–3.13 (m, 1H), 2.56 (d, *J* = 17.0 Hz, 1H), 2.45 (d, *J* = 18.3 Hz, 1H), 2.38 (s, 3H), 2.36–2.30 (m, 1H), 2.22–2.12 (m, 1H), 1.85 (d, *J* = 13.9 Hz, 1H), 1.64 (dd, *J* = 27.6, 14.6 Hz, 2H), and 1.38–1.26 (m, 1H). Some signals were split due to the presence of N-invertomers, **^13^C NMR** (101 MHz, DMSO-d_6_), δ [ppm]: 204.4, 203.8, 161.1, 161.0, 138.5, 137.5, 136.7, 131.8, 131.5, 131.0, 130.3, 129.0, 54.8, 54.0, 50.5, 49.6, 43.4, 33.9, 33.4, 28.6, 22.6, 14.1, 13.8, and 8.7. **HRMS** (ESI): calcd. for C_20_H_24_Cl_2_N_5_O_2_^+^ [M+H]^+^ 436.1302; found: 436.1302.

**1-(3-Chlorophenyl)-5-methyl-*N*-(2-(3-oxo-9-azabicyclo [3.3.1]nonan-9-yl)ethyl)-1*H*-1,2,3-triazole-4-carboxamide hydrochloride** (**5E**) was synthesized from *N*-(2-aminoethyl)-1-(3-chlorophenyl)-5-methyl-1*H*-1,2,3-triazole-4-carboxamide hydrochloride (**5D**), according to the General Procedure 5, and obtained as a white solid (0.76 g, 87%); mp: 245–246 °C and R_f_ = 0.82 (methanol/DCM: 1/10, *v*/*v*), determined for the free amine. **^1^H NMR** (400 MHz, DMSO-d_6_), δ [ppm]: 11.90, 11.40 (s, 1H), 9.10–8.80 (m, 1H), 7.82 (s, 1H), 7.76–7.58 (m, 3H), 4.21–4.97 (m, 2H), 3.93–3.70 (m, 2H), 3.67–3.53 (m, 2H), 3.51–3.40 (m, 1H), 3.22–3.07 (m, 1H), 2.56 (s, 3H), 2.56–2.54 (m, 1H), 2.45–2.31 (m, 2H), 2.22–2.08 (m, 1H), 1.92–1.75 (m, 1H), 1.63 (dd, *J* = 31.0, 11.6 Hz, 2H), and 1.40–1.23 (m, 1H). Some signals were split due to the presence of N-invertomers, **^13^C NMR** (101 MHz, DMSO-d_6_), δ [ppm]: 204.9, 204.3, 161.6, 138.4, 137.6, 136.9, 134.4, 131.9, 130.6, 125.8, 124.7, 55.3, 54.4, 51.0, 50.0, 43.8, 34.4, 34.0, 29.0, 23.1, 14.6, and 9.7. **HRMS** (ESI): calcd. for C_20_H_25_ClN_5_O_2_^+^ [M+H]^+^ 402.1691; found: 402.1688.

**1-(2-Chlorophenyl)-5-methyl-*N*-(2-(3-oxo-9-azabicyclo [3.3.1]nonan-9-yl)ethyl)-1*H*-1,2,3-triazole-4-carboxamide hydrochloride** (**6E**) was synthesized from *N*-(2-aminoethyl)-1-(2-chlorophenyl)-5-methyl-1*H*-1,2,3-triazole-4-carboxamide hydrochloride (**6D**), according to the General Procedure 5, and obtained as a white solid (0.78 g, 89%); mp: 242–243 °C and R_f_ = 0.82 (methanol/DCM: 1/10, *v*/*v*), determined for the free amine. **^1^H NMR** (500 MHz, DMSO-d_6_), δ [ppm]: 11.69, 11.13 (s, 1H), 9.03–8.96 (m, 1H), 7.87–7.81 (m, 1H), 7.77–7.69 (m, 2H), 7.69–7.61 (m, 1H), 4.17–4.04 (m, 2H), 3.88–3.78 (m, 2H), 3.63–3.54 (m, 2H), 3.49–3.40 (m, 1H), 3.16 (dd, *J* = 18.2, 6.3 Hz, 1H), 2.57 (d, *J* = 17.2 Hz, 1H), 2.46 (d, *J* = 18.3 Hz, 1H), 2.37 (s, 3H), 2.36–2.28 (m, 1H), 2.22–2.11 (m, 1H), 1.85 (d, *J* = 13.9 Hz, 1H), 1.76–1.53 (m, 2H), and 1.39–1.26 (m, 1H). Some signals were split due to the presence of N-invertomers, **^13^C NMR** (126 MHz, DMSO-d_6_), δ [ppm]: 204.3, 203.8, 161.1, 138.2, 137.4, 132.8, 132.4, 130.5, 130.4, 129.7, 128.8, 54.8, 54.0, 50.6, 49.6, 43.4, 33.9, 33.4, 28.5, 22.6, 14.1, 13.8, and 8.7. **HRMS** (ESI): calcd. for C_20_H_25_ClN_5_O_2_^+^ [M+H]^+^ 402.1691; found: 402.1689.

***N*-(2-(3-Oxo-9-azabicyclo [3.3.1]nonan-9-yl)ethyl)-1-phenyl-5-(trifluoromethyl)-1*H*-1,2,3-triazole-4-carboxamide hydrochloride** [**1E(CF3)**] was synthesized from *N*-(2-aminoethyl)-1-phenyl-5-(trifluoromethyl)-1*H*-1,2,3-triazole-4-carboxamide hydrochloride [**1D(CF_3_)**], according to the General Procedure 5, and obtained as a white solid (0.83 g, 91%); mp: 236–237 °C and R_f_ = 0.84 (methanol/DCM: 1/10, *v*/*v*), determined for the free amine. **^1^H NMR** (500 MHz, DMSO-d_6_), δ [ppm]: 11.94, 11.43 (s, 1H), 9.39–9.32 (m, 1H), 7.72–7.66 (m, 5H), 4.10 (d, *J* = 22.2 Hz, 2H), 3.93–3.86 (m, 2H), 3.65–3.59 (m, 2H), 3.51–3.43 (m, 1H), 3.16 (dd, *J* = 18.2, 6.2 Hz, 1H), 2.56 (d, *J* = 17.2 Hz, 1H), 2.46 (d, *J* = 18.3 Hz, 1H), 2.42–2.33 (m, 1H), 2.15 (t, *J* = 14.1 Hz, 1H), 1.84 (d, *J* = 13.7 Hz, 1H), 1.65 (dd, *J* = 38.7, 14.3 Hz, 2H), and 1.39–1.27 (m, 1H). Some signals were split due to the presence of N-invertomers, **^13^C NMR** (126 MHz, DMSO-d_6_), δ [ppm]: 204.3, 203.7, 158.3, 141.7, 135.3, 131.4, 129.6, 127.4 (m), 126.2, 118.9 (q), 54.9, 54.0, 50.2, 43.4, 34.4, 33.9, 28.5, 22.6, 14.1, and 13.8. ^19^F NMR (471 MHz, DMSO-d_6_), δ [ppm]: −54.6. **HRMS** (ESI): calcd. for C_20_H_23_F_3_N_5_O_2_^+^ [M+H]^+^ 422.1798; found: 422.1799.

***N*-(2-(3-Oxo-9-azabicyclo [3.3.1]nonan-9-yl)ethyl)-1-(*p*-tolyl)-5-(trifluoromethyl)-1*H*-1,2,3-triazole-4-carboxamide hydrochloride** [**2E(CF3)**] was synthesized from *N*-(2-aminoethyl)-1-phenyl-5-(trifluoromethyl)-1*H*-1,2,3-triazole-4-carboxamide hydrochloride [2D(CF_3_)], according to the General Procedure 5, and obtained as a white solid (0.85 g, 90%); mp: 212–213 °C and R_f_ = 0.87 (methanol/DCM: 1/10, *v*/*v*), determined for the free amine. **^1^H NMR** (500 MHz, DMSO-d_6_), δ [ppm]: 11.91, 11.39 (s, 1H), 9.38–9.27 (m, 1H), 7.54 (d, *J* = 8.1 Hz, 2H), 7.46 (d, *J* = 8.2 Hz, 2H), 4.09 (d, *J* = 22.0 Hz, 2H), 3.94–3.82 (m, 2H), 3.66–3.55 (m, 2H), 3.47 (dd, *J* = 17.0, 5.9 Hz, 1H), 3.15 (dd, *J* = 18.1, 6.1 Hz, 1H), 2.56 (d, *J* = 17.1 Hz, 1H), 4.49–2.44 9 (m, 1H), 2.43 (s, 3H), 2.40–2.33 (m, 1H), 2.15 (t, *J* = 14.2 Hz, 1H), 1.84 (d, *J* = 13.7 Hz, 1H), 1.65 (dd, *J* = 38.6, 14.3 Hz, 2H), and 1.40–1.26 (m, 1H). Some signals were split due to the presence of N-invertomers, **^13^C NMR** (126 MHz, DMSO-d_6_), δ [ppm]: 204.3, 203.7, 158.3, 141.7, 141.3, 132.8, 130.0, 127.3 (q), 125.9, 118.9 (q), 54.8, 54.0, 50.2, 49.3, 43.3, 34.3, 33.9, 28.4, 22.6, 20.8, 14.1, and 13.8. **^19^F NMR** (471 MHz, DMSO-d_6_), δ [ppm]: −54.7. **HRMS** (ESI): calcd. for C_21_H_25_F_3_N_5_O_2_^+^ [M+H]^+^ 436.1955; found: 436.1956.

### 3.3. Biological Activity

#### 3.3.1. In Vitro Inhibition Studies on AChE

The selected spectroscopic method was described in detail previously [53]. We used a kit for testing AChE inhibitors (catalogue number MAK324), together with the enzyme (purified AChE—catalogue number C3389) and Donepezil purchased from Sigma-Aldrich (St. Louis, MO, USA). Absorbance readings were taken using an Infinite M200 fluorescence spectrophotometer (TECAN, Männedorf, Switzerland). The tests were performed on a transparent 96-well flat-bottom plate. The final concentrations of Donepezil and the tested compounds were 1, 10, 20, 50, and 100 mM. The absorbance of each sample was measured at 412 nm after 0 min and after 10 min. Based on the data obtained, the concentration of the compound that caused a 50% decrease in activity, i.e., the IC_50_ value (µM), was calculated. The experiment was repeated three times.

#### 3.3.2. In Vitro Inhibition Studies on β-Secretase (BACE1)

To test the activity of new compounds against BACE1, a kit from Sigma-Aldrich (catalogue number CS0010) was used and applied as described [53], using solutions with the same concentrations of compounds as in the AChE test. The test was performed using a black 96-well microplate. The increase in fluorescence signal after cleavage of the substrate by BACE1 [52] using an Infinite M200 fluorescence spectrophotometer (TECAN, Männedorf, Switzerland) (ex. 320 nm; em. 405 nm) in three replicate experiments with a negative control (no enzyme) and a positive control (enzymatic activity added). A 50% decrease in BACE1 activity was calculated as IC_50_ (µM).

### 3.4. Molecular Docking

Molecular docking with a variable window was carried out, i.e., the search area changed its coordinates. The area was a cube with the following dimensions: 20 × 20 × 20 Å. The initial coordinates of its center were x = −45, y = 40, and z = −24. The cube was then moved using an in-house script:

- From x − 6 to x + 6 every two units.
- From y − 6 to y + 6 every two units.
- From z − 6 to z + 6 every two units.

This process was repeated for each tested ligand in order to find the most favorable binding energy. To increase accuracy, the EXHAUSTIVENESS parameter was set to 100 (default 8).

**Figure 16:**
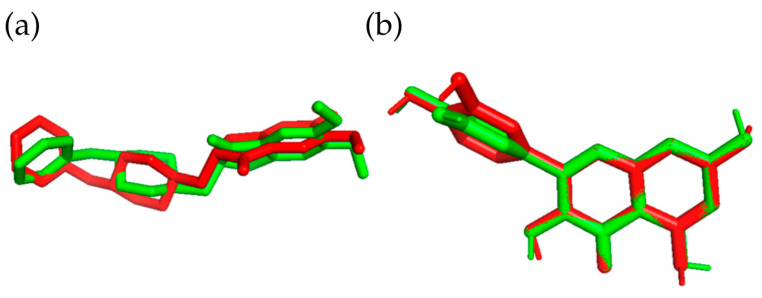
Comparison of crystal structures (red) and those obtained after docking (green) for (**a**) Donepezil docked to AChE and (**b**) Quercetin docked to BACE1. The RMSD for (**a**) is 1.55 Å and for (**b**) is 0.10 Å. Both values were considered satisfactory.

Docking was performed to the AChE protein, whose structure was downloaded from the PDB database (PDB: 7E3H, Resolution 2.45 Å [64]). The protein was prepared for docking by performing a redocking procedure, removing docked ligands and water molecules. Polar hydrogen atoms and Kollman charges were added to it. Docking was carried out using AutoDock Vina 1.2.5 [65,66]. The BIOVIA Discovery Studio Visualizer [67] was used to analyze and visualize ligand–protein interactions. On the other hand, the program Pymol 2.5.5 [68] was used to visualize the positions of the ligands analyzed in the statistical analysis of docking and to graphically represent the position of the reference ligand in the validation process, which consisted of comparing the Donepezil and the Quercetin structure obtained after docking and the crystal structure (Figure 16). Statistica 13.0 (StatSoft; Tulsa, OK, USA) was used for statistical analysis. An analogous docking procedure was carried out for the BACE1 protein downloaded from the PDB database (PDB: 5HU1, Resolution: 1.5 Å [69]). The coordinates of the center of the cube were x = 25, y = 10, and z = 19, and they changed according to the same scheme as for docking to AChE.

### 3.5. Molecular Dynamics

Molecular dynamics simulations were performed with NAMD 2.14 [70] and VMD 1.9.3 [71] using CHARM22 force fields. Ligands after the docking process were prepared for analysis using CHARMM—GUI (version 3.8) [72,73]. The process of preparing the system for simulation included the following:

- Surrounding the system with a solvation shell.
- Ionizing the system with 0.15 M NaCl to make the conditions similar to the physiological environment.
- Running a simulation in which the system was gradually heated from 0 K to 310 K (MINIMIZE—50,000 and Equalibration—1 ns)

Next, the actual simulations were carried out, with each simulation lasting 100 ns and the duration of one step being 2 fs. During the simulations, a constant temperature of 310 K and a constant pressure of one atmosphere were maintained. This was possible thanks to the use of Langevin dynamics—damping factor equal to 5 (1/ps) and Langevin piston decay period equal to 100 fs. Both the docking methodology and MD were verified in studies of other compounds in the context of AChE and BACE1 inhibition [52], as well as in the context of antibacterial and antifungal activity [74].

### 3.6. QSAR Modeling

ADMETlab 2.0 [75] was used to perform the ADMET analysis.

## 4. Conclusions

A series of triazole derivatives was synthesized and evaluated as potential dual inhibitors of AChE and BACE1. The compounds exhibited balanced inhibitory activity against both enzymes, indicating their potential relevance for the treatment of Alzheimer’s disease. Among them, compounds **1D** and **2E(CF_3_)** were identified as the most promising candidates and selected for detailed computational studies. Although their IC_50_ values were higher than those of the reference ligands, their docking energies were comparable or, in some cases, more favorable.

Docking and molecular dynamics analyses indicate that the triazole derivatives interact with the target enzymes via mechanisms distinct from those of Donepezil and Quercetin. For AChE, interactions with Tyr-124 were most frequently observed, whereas for BACE1, Thr-133 and Thr-293 emerged as key residues involved in triazole binding. Compounds **1D** and **2E(CF_3_)** exhibited greater conformational flexibility than Donepezil, enabling multiple binding modes, which was reflected in broader energy distributions of individual binding poses.

Principal component analysis (PCA), integrating docking scores, ligand efficiency, and molecular dynamics descriptors, enabled clear differentiation of the triazole derivatives from the reference ligands, despite similar docking energies. Molecular dynamics simulations further suggest that the investigated ligands exert a stabilizing effect on enzyme structures, potentially contributing to enhanced inhibitory activity, particularly in the case of AChE. The triazole derivatives formed hydrogen bonds more frequently than Donepezil but less frequently than Quercetin, which is consistent with the greater number of hydrogen-bond-donating groups present in the quercetin molecule.

In addition, compound **1D** demonstrated lower predicted toxicity than **2E(CF_3_)**, while both compounds showed high predicted blood–brain barrier permeability, a crucial property given the localization of the target enzymes. Overall, compounds **1D** and **2E(CF_3_)** emerge as promising investigational candidates for Alzheimer’s disease therapy, although further optimization and experimental validation are required.

## Figures and Tables

**Table 1:** Yields of intermediate and final products in the synthesis (Scheme 1).

			Steps a, b			Step c		Steps d, e		Steps f, g	
Entry	S1	R^1^	P1	Y1 (%)	R^2^	P2	Y2 (%)	P3	Y3 (%)	P4	Y4 (%)
1	**1A**	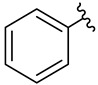	**1B**	97	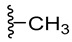	**1C**	94	**1D**	89	**1E**	89
2	**2A**	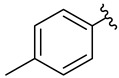	**2B**	96	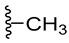	**2C**	92	**2D**	86	**2E**	92
3	**3A**	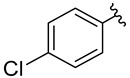	**3B**	95	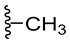	**3C**	92	**3D**	93	**3E**	88
4	**4A**	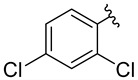	**4B**	94	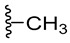	**4C**	91	**4D**	91	**4E**	85
5	**5A**	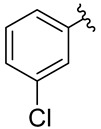	**5B**	94	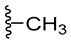	**5C**	94	**5D**	92	**5E**	87
6	**6A**	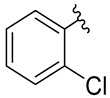	**6B**	96	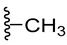	**6C**	93	**6D**	94	**6E**	89
7	**1A**	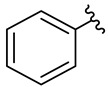	**1B**	97	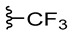	**1C(CF_3_)**	87	**1D(CF_3_)**	93	**1E(CF_3_)**	91
8	**2A**	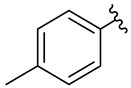	**2B**	96	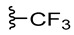	**2C(CF_3_)**	90	**2D(CF_3_)**	92	**2E(CF_3_)**	90

**Table 2:** In vitro inhibition of AChE and BACE1 of the new and reference compounds.

No.	Compound	eeAChE IC_50_ (µM) ^a^			esBACE1 IC_50_ (µM) ^a^		
		IC_50_ (µM)	Docking Score (kcal/mol)	Ligand Efficiency (kcal/mol*atom)	IC_50_ (µM)	Docking Score (kcal/mol)	Ligand Efficiency (kcal/mol*atom)
1	**1E**	1.055 ± 0.03	−11.09	−0.411	11.11 ± 3.02	−8.014	−0.297
2	**2E**	0.853 ± 0.01	−11.37	−0.406	13.41 ± 2.44	−8.197	−0.293
3	**3E**	0.789 ± 0.02	−11.21	−0.400	12.11 ± 2.25	−8.077	−0.288
4	**4E**	1.326 ± 0.03	−11.33	−0.391	28.44 ± 2.58	−8.018	−0.276
5	**5E**	0.699 ± 0.01	−11.26	−0.402	10.08 ± 2.21	−8.199	−0.293
6	**6E**	0.703 ± 0.02	−11.24	−0.401	11.58 ± 1.24	−8.031	−0.287
7	**1E(CF#)**	0.732 ± 0.02	−11.68	−0.377	11.10 ± 1.12	−8.426	−0.272
8	**2E(CF3)**	0.599 ± 0.01	−11.95	−0.373	9.25 ± 2.63	−8.596	−0.267
9	**1D**	0.590 ± 0.02	−8.236	−0.458	9.85 ± 1.93	−6.914	−0.384
10	**2D**	0.603 ± 0.04	−8.671	−0.456	14.56 ± 2.54	−6.911	−0.364
11	**3D**	0.583 ± 0.03	−8.336	−0.439	10.50 ± 2.47	−6.756	−0.356
12	**4D**	0.555 ± 0.02	−8.401	−0.420	10.25 ± 2.45	−6.545	−0.327
13	**5D**	0.568 ± 0.01	−8.372	−0.441	13.25 ± 2.58	−6.917	−0.364
14	**6D**	0.655 ± 0.02	−8.041	−0.423	12.11 ± 1.85	−7.069	−0.372
17	**1D(CF3)**	0.745 ± 0.02	−8.951	−0.426	9.47 ± 2.31	−6.968	−0.332
18	Donepezil ^b^	0.046 ± 0.013 [53]	−11.69	−0.418	- ^c^	−8.684	−0.310
19	Tacrine ^b^	0.274 ± 0.08 [53]	−8.845	−0.590	- ^c^	−6.845	−0.456
20	Quercetin ^b^	- ^c^	−9.567	−0.435	4.89 ± 2.31 [53]	−8.323	−0.378

^a^ AChE from Electrophorus electricus (electric eel); IC_50_, inhibitor concentration (mean ± SD of three independent experiments) resulting in 50% inhibition of AChE. ^b^ BACE1 from equine serum; IC_50_, inhibitor concentration (mean ± SD of three independent experiments) resulting in 50% inhibition of BACE1. ^c^ n.d., not determined.

**Table 3:** Positions of ligands obtained by molecular docking with a variable window to AChE.

	1D	2E_CF3_	Donepezil	Quercetin
Position 1	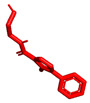	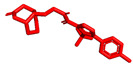	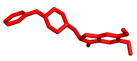	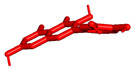
Position 2	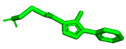	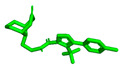	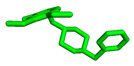	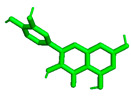
Position 3	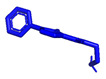	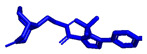	-	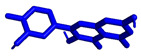

**Table 4:** Abundance and percentage of each position of ligands docked to AChE.

	1D	2E_CF3_	Donepezil	Quercetin
Position 1	58 (58%)	81 (60%)	124 (76.07%)	92 (41.26%)
Position 2	40 (40%)	47 (34.81%)	39 (29.93%)	11 (4.93%)
Position 3	2 (2%)	7 (5.19%)		120 (53.81%)

**Table 5:** Median values of ligand–protein binding energy for most occurring positions of ligands docked to AChE. The minimum and maximum (min/max) energy values for each position are also given. In order to check the normality of the energy distribution, the Shapiro–Wilk test was performed—*p* < 0.05 means that the distribution is not normal; *p* > 0.05 means that the distribution is normal.

	1D	2E_CF3_	Donepezil	Quercetin
Position 1	−8.0995 −8.236/−8.01 *p* > 0.05	−11.8 −11.95/−11.02 *p* < 0.05	−11.385 −11.69/−11.09 *p* < 0.05	−9.52 −9.567/−9.417 *p* < 0.05
Position 2 (for Quercetin Position 3)	−8.0315 −8.062/−8 *p* > 0.05	−11.67 −11.81/−11.24 *p* < 0.05	−11.25 −11.33/−11.00 *p* < 0.05	−9.302 −9.348/−9.252 *p* > 0.05

**Table 6:** Positions of ligands obtained by molecular docking with a variable window to BACE1.

	1D	2E_CF3_	Donepezil	Quercetin
Position 1	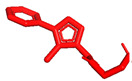	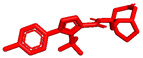	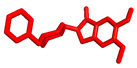	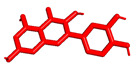
Position 2	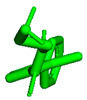	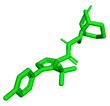	-	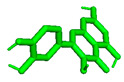
Position 3	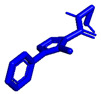	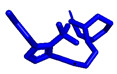	-	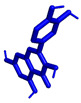

**Table 7:** Abundance and percentage of each position of ligands docked to BACE1.

	1D	2E_CF3_	Donepezil	Quercetin
Position 1	289 (97.64%)	11 (9.09%)	215 (100%)	119 (44.07%)
Position 2	2 (0.68%)	91 (81.82%)	-	119 (44.07%)
Position 3	5 (1.68%)	11 (9.09%)	-	32 (11.86%)

**Table 8:** Median values of ligand–protein binding energy for most occurring positions of ligands docked to BACE1. The minimum and maximum (min/max) energy values for each position are also given. In order to check the normality of the energy distribution, the Shapiro–Wilk test was performed—*p* < 0.05 means that the distribution is not normal; *p* > 0.05 means that the distribution is normal.

	1D	2E_CF3_	Donepezil	Quercetin
Position 1	−6.829 −6.914/−6.542 *p* < 0.05	−8.503 −8.596/−8.131 *p* < 0.05	−8.539 −8.684/−8.002 *p* < 0.05	−8.292 −8.323/−8.002 *p* < 0.05
Position 2	-	−8.299 −8.364/−8.001 *p* < 0.05	-	−8.134 −8.237/−8.039 *p* < 0.05

**Table 9:** Description of hydrogen bonds formed during the simulation of complexes with AChE, taking into account the bond donor and acceptor, the type of chain (main and side) participating in the bond, and the percentage of frames in which the bond occurs.

Ligand	Donor	Acceptor	Occupancy (%)
1D	Tyr-337-Side	1D-Side	12.20
	1D-Side	Tyr-124-Side	8.10
	Tyr-124-Side	1D-Side	5.40
	Trp-286-Side	1D-Side	1.40
2E_CF3_	Tyr-124-Side	2E_CF3_-Side	5.00
	2E_CF3_-Side	Tyr-124-Side	1.40
Donepezil	Gly-345-Main	Donepezil-Side	1.10
Quercetin	Quercetin-Side	Glu-202-Side	15.90
	Quercetin-Side	Val-73-Main	7.40
	Quercetin-Side	Hsd-447-Main	7.10
	Quercetin-Side	Tyr-133-Side	6.80
	Quercetin-Side	Ser-125-Side	4.20
	Thr-75-Side	Quercetin-Side	2.10
	Ser-125-Side	Quercetin-Side	2.00
	Gly-121-Main	Quercetin-Side	1.80
	Asn-87-Side	Quercetin-Side	1.60

**Table 10:** Description of hydrogen bonds formed during the simulation of complexes with BACE1, taking into account the bond donor and acceptor, the type of chain (main and side) participating in the bond, and the percentage of frames in which the bond occurs.

Ligand	Donor	Acceptor	Occupancy (%)
1D	Lys-310-Side	1D-Side	4.60
	Thr-293-Main	1D-Side	2.90
	Thr-293-Side	1D-Side	1.90
	Asn-294-Side	1D-Main	1.30
2E_CF3_	Thr-133-Side	2E_CF3_-Side	3.80
	Thr-133-Main	2E_CF3_-Side	2.00
	Gly-135-Main	2E_CF3_-Side	1.90
	Gln-134-Main	2E_CF3_-Side	1.10
Donepezil	Lys-168-Side	Donepezil-Side	1.70
	Gln-134-Side	Donepezil-Side	1.10
Quercetin	Quercetin-Side	Asp-93-Side	69.90
	Quercetin-Side	Val- -92-Main	49.30
	Quercetin-Side	Gln-134-Main	19.70
	Quercetin-Side	Ser-290-Main	4.30

**Table 11:** Pharmacokinetic parameter values of the ligands studied (H—high value, M—medium value, L—low value, Y—yes, and N—no).

Ligand	Oral Toxicity for Rats	Carcinogenicity/Hepatotoxicity/Dermal Toxicity/Inhalation Toxicity/AMES/Eye Toxicity	Principle: Lipinski/Pfizer/GSK/Golden Triangle	HIA/F_20%_/PPB/BBB/Pgp_inh_/Pgp_sub_
1D	H	L/L/L/H/H/L	Y/Y/Y/Y	L/L/L/H/L/L
2E_CF3_	H	H/H/M/H/H/L	Y/Y/N/Y	L/L/L/H/H/L
Donepezil	L	L/M/L/H/L/L	Y/N/N/Y	L/L/H/H/H/H
Quercetin	L	L/L/H/L/M/L	Y/Y/Y/Y	L/H/H/L/L/L

## Data Availability

The original contributions presented in this study are included in the article/Supplementary Material. Further inquiries can be directed to the corresponding author.

## Supplementary Materials

The following supporting information can be downloaded at https://www.mdpi.com/article/10.3390/ijms27021008/s1.

## Abbreviations

The following abbreviations are used in this manuscript:

AD	Alzheimer’s disease
ACh	Acetylcholine
AChE	Acetylcholinesterase
BACE1	β site amyloid precursor protein cleaving enzyme 1; β secretase
MAO	Monoamine oxidase
Wnt	Wingless and Int-1 gene family
NMDA	N-methyl-D-aspartate
5-HT	5-hydroxytryptamine; serotonin
MTDLs	Multi-target directed ligands
CNS	Central nervous system
MD	Molecular dynamics
RMSD	Root mean square deviation
RMSF	Root mean square fluctuation
SASA	Solvent accessible surface area
Rg	Radius of gyration
PCA	Principal component analysis
ADMET	Absorption, distribution, metabolism, excretion, and toxicity
PPB	Plasma protein binding
BBB	Blood–brain barrier
Pgp	P-glycoprotein
TPSA	Topological polar surface area
