# Supplementary material for: Design, Synthesis, and Biological Evaluation of Novel Acetylcholinesterase and β-Secretase 1 Inhibitors
Source: Int J Mol Sci. 2026 Jan 20;27(2):1008. doi: 10.3390/ijms27021008 (PMC12842540; doi:10.3390/ijms27021008)
Supplement: Supplementary file 1 [file ijms-27-01008-s001.zip › ijms-4062851-supplementary.pdf]

Supporting Information

Design, synthesis and biological evaluation of novel acetylcholinesterase and β-secretase inhibitors

Danuta Drozdowska [1,*], Damian Pawelski [1], Agnieszka Wróbel-Tałałaj [1], Marta Płońska-Brzezińska [1], Beata Kolesińska [2], Ryszard Łaźny [3], Barbara Seroka [3], Cezary Parzych [4], Artur Ratkiewicz [4]

[1] Department of Organic Chemistry, Medical University of Bialystok, 15-222 Bialystok, Poland
[2] Institute of Organic Chemistry, Lodz University of Technology, 90-924 Lodz, Poland
[3] Department of Polymers and Organic Synthesis, Faculty of Chemistry, University of Bialystok, 15-245 Bialystok, Poland
[4] Department of Physical Chemistry, Faculty of Chemistry, University of Bialystok, 15-245 Bialystok, Poland
* Correspondence: danuta.drozdowska@umb.edu.pl (D.D.)

List of Contents

I. Copies of NMR and HRMS spectra **3**

^{1}H NMR spectra of compounds **1B**-**6B** 3

^{1}H NMR and ^{13}C NMR spectra of compounds **1C**-**6C** 6

^{1}H NMR, ^{13}C NMR and ^{19}F NMR spectra of compounds **1C(CF$_3$)** and **2C(CF$_3$)** 12

^{1}H NMR, ^{13}C NMR and HRMS spectra of compounds **1D-6D** 15

^{1}H NMR, ^{13}C NMR, ^{19}F NMR and HRMS spectra of compounds **1D(CF$_3$)** and **2D(CF$_3$)** 24

^{1}H NMR, ^{13}C NMR and HRMS spectra of compounds **1E-6E** 28

^{1}H NMR, ^{13}C NMR, ^{19}F NMR and HRMS spectra of compounds **1E(CF$_3$)** and **2E(CF$_3$)** 37

Copies of NMR and HRMS spectra

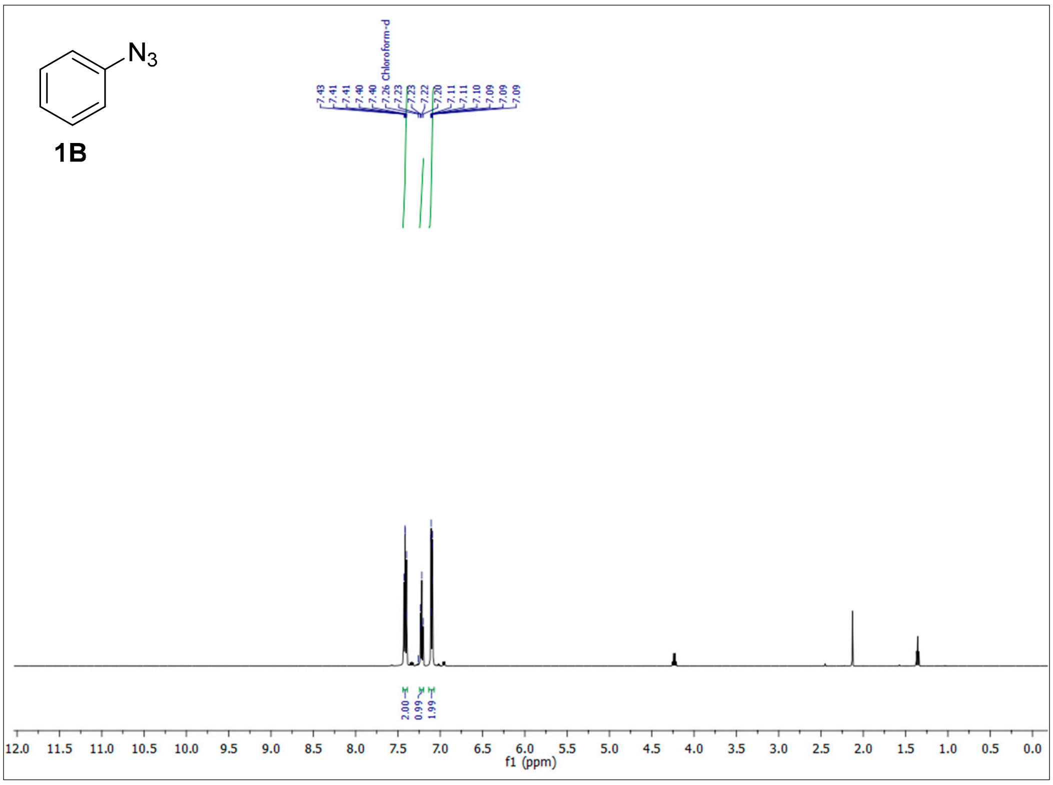

Figure S1. ^{1}H NMR (500 MHz, $CDCl_3$) spectrum of azidobenzene (**1B**).

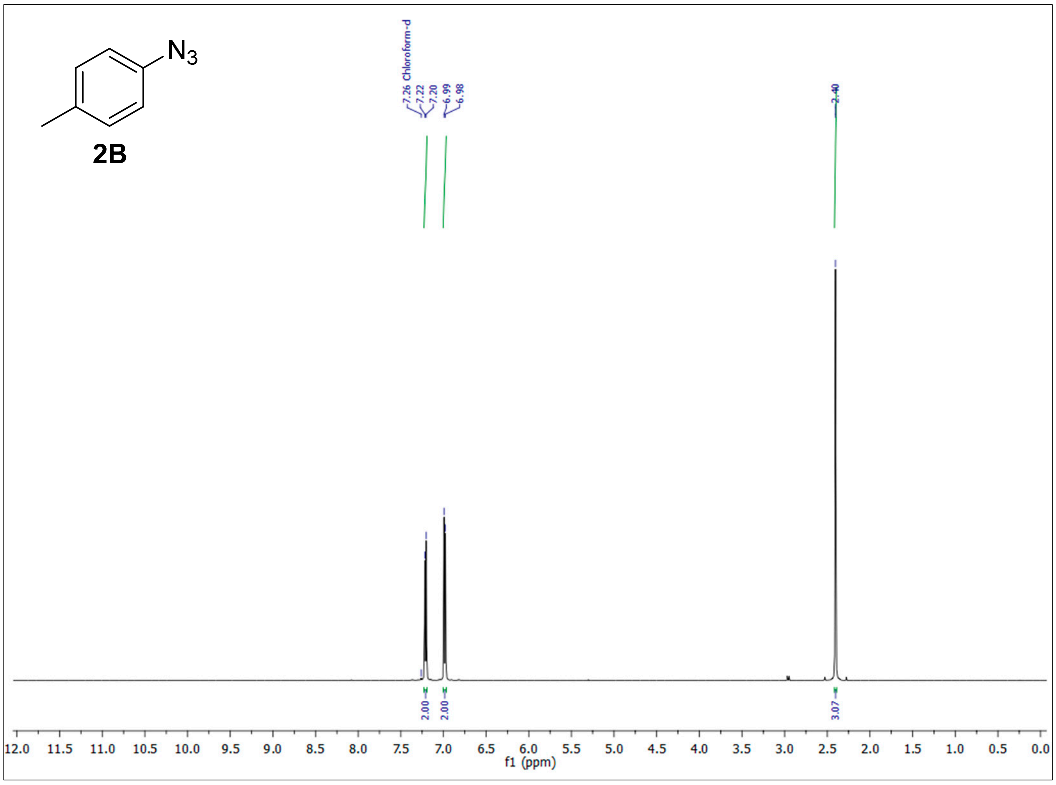

Figure S2. ^{1}H NMR (500 MHz, $CDCl_3$) spectrum of 1-azido-4-methylbenzene (**2B**).

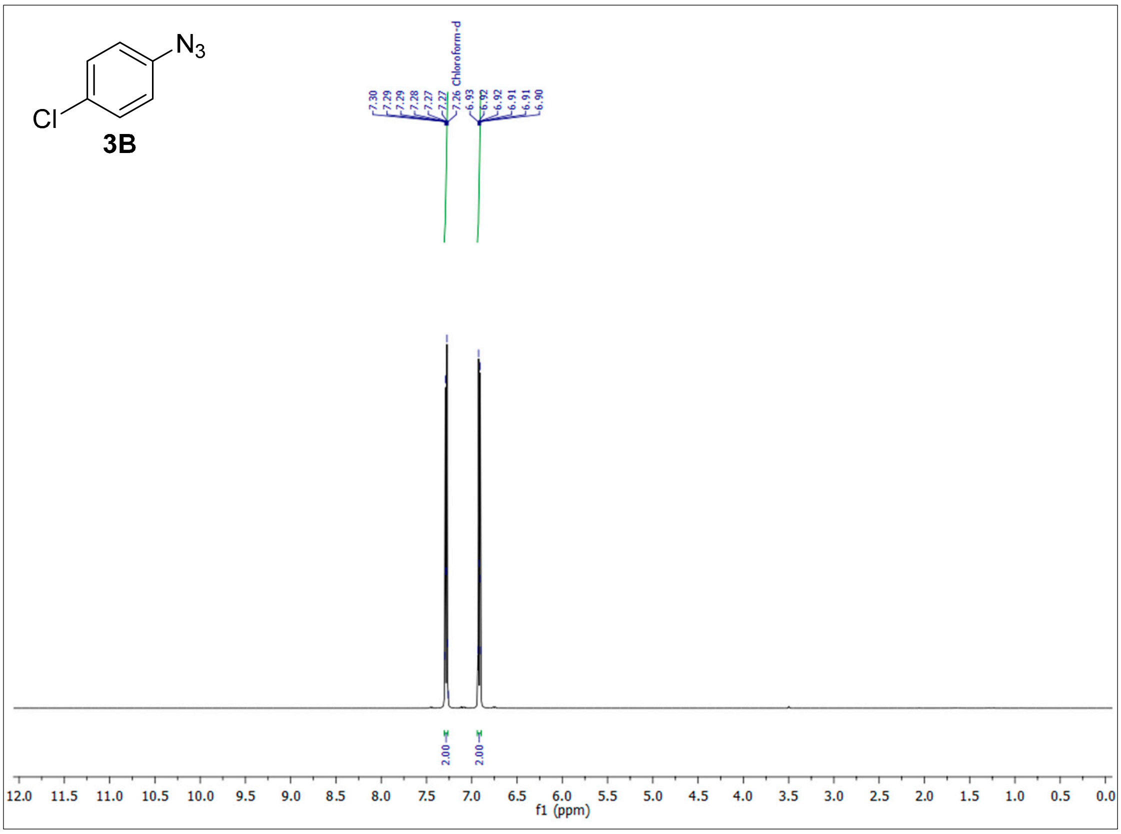

Figure S3. ^{1}H NMR (500 MHz, $CDCl_3$) spectrum of 1-azido-4-chlorobenzene (**3B**).

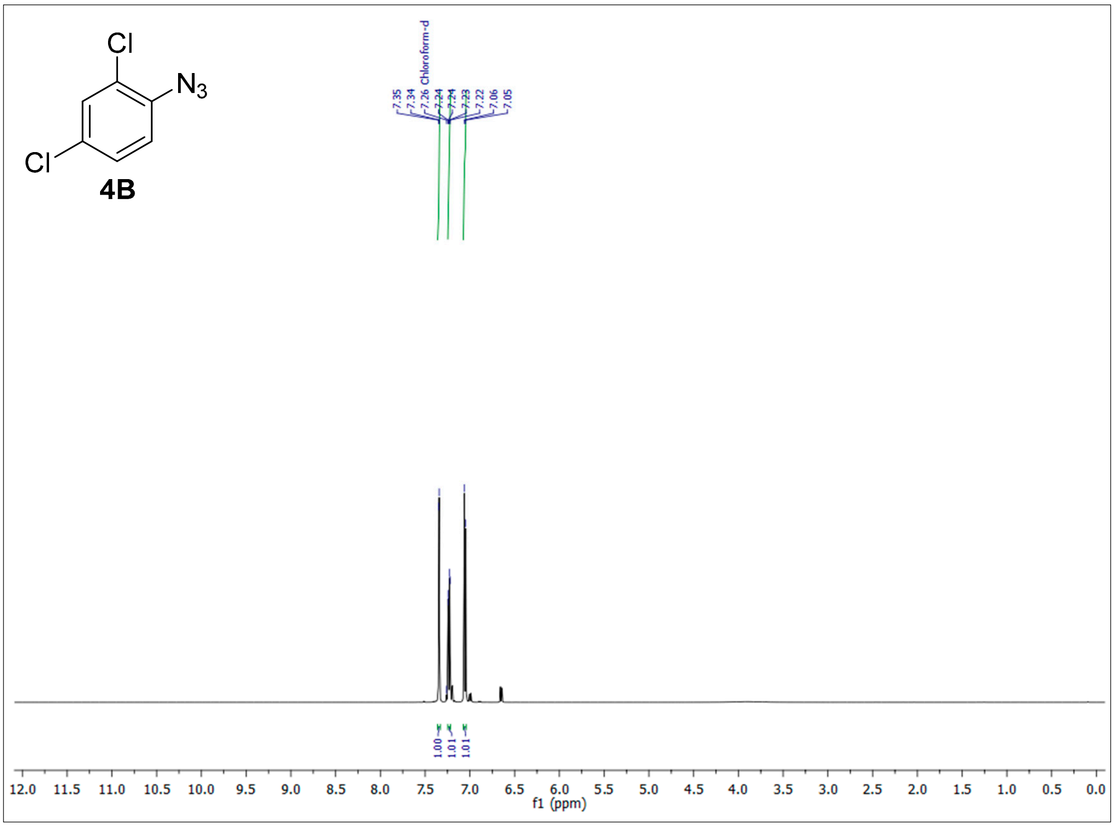

Figure S4. ^{1}H NMR (500 MHz, $CDCl_3$) spectrum of 1-azido-2,4-dichlorobenzene (**4B**).

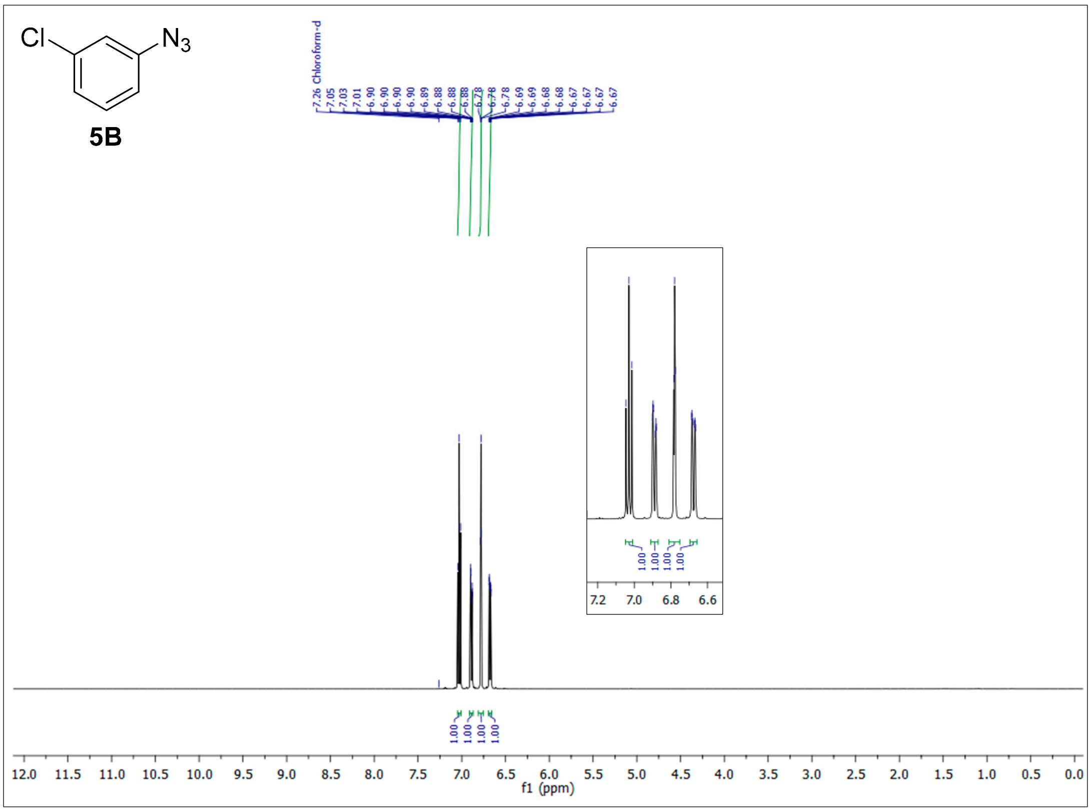

Figure S5. ^{1}H NMR (500 MHz, $CDCl_3$) spectrum of 1-azido-3-chlorobenzene (**5B**).

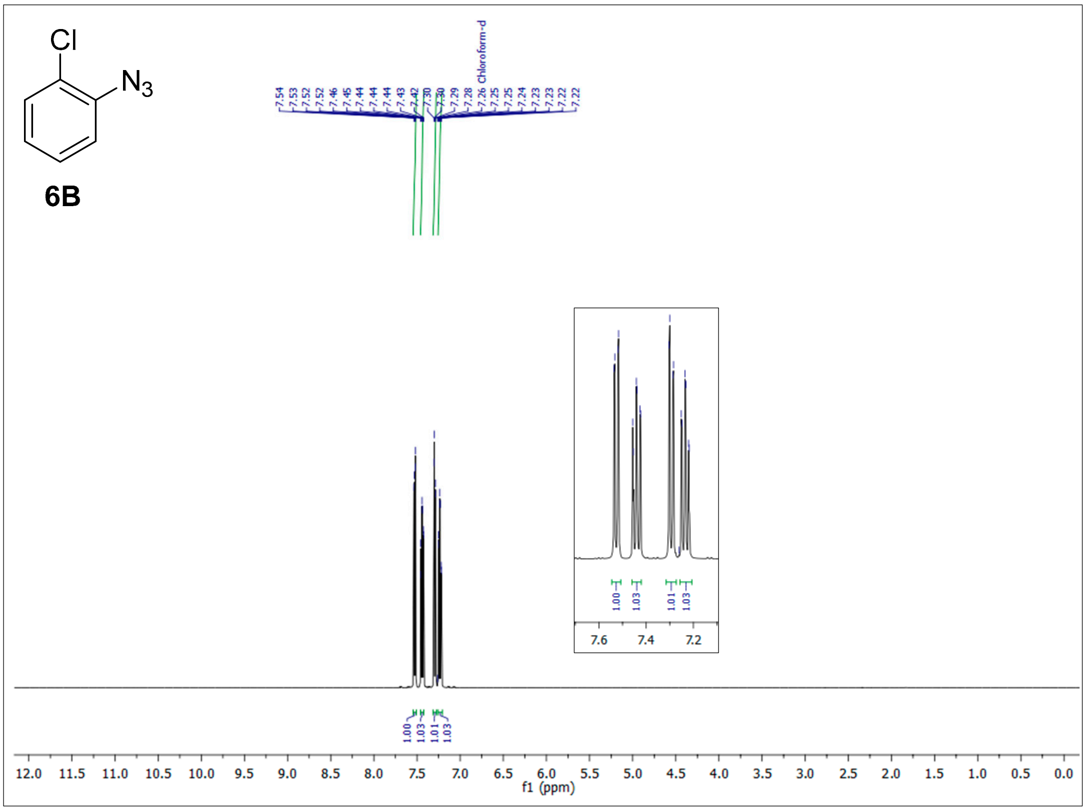

Figure S6. ^{1}H NMR (500 MHz, $CDCl_3$) spectrum of 1-azido-2-chlorobenzene (**6B**).

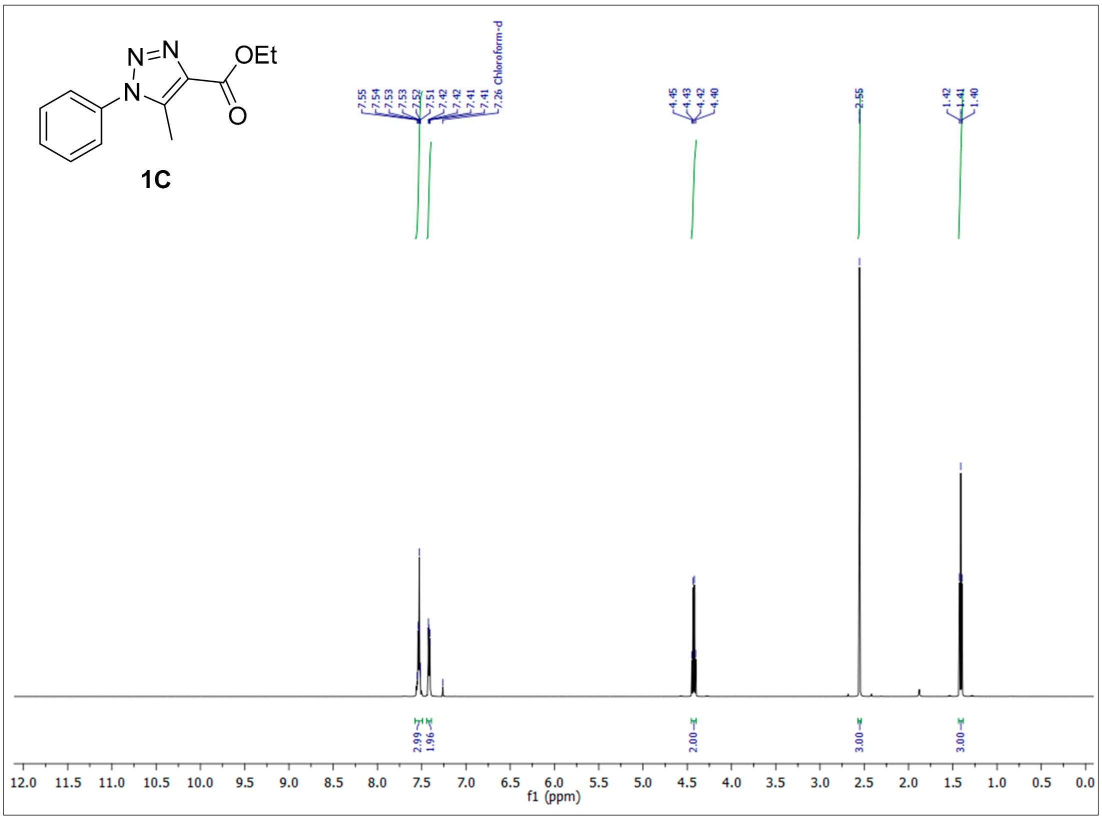

Figure S7. ^{1}H NMR (500 MHz, $CDCl_3$) spectrum of ethyl 5-methyl-1-phenyl-1*H*-1,2,3-triazole-4-carboxylate (**1C**).

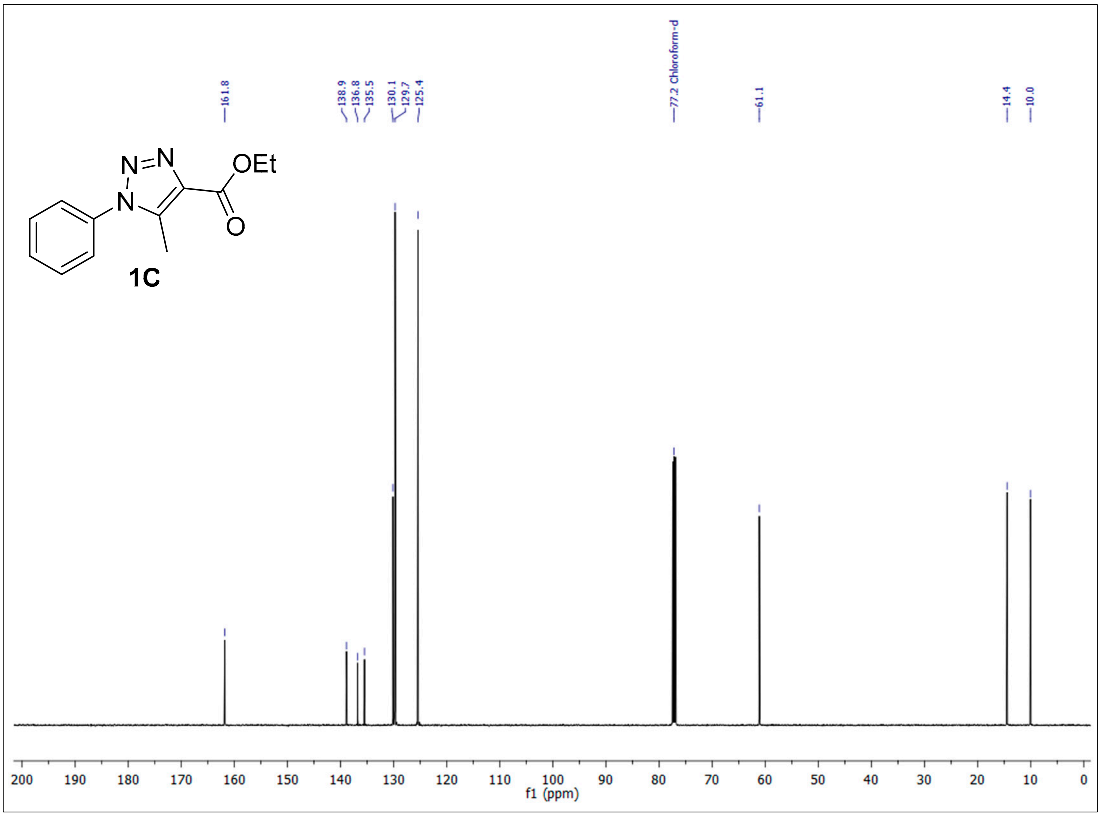

Figure S8. ^{13}C NMR (126 MHz, $CDCl_3$) spectrum of ethyl 5-methyl-1-phenyl-1*H*-1,2,3-triazole-4-carboxylate (**1C**).

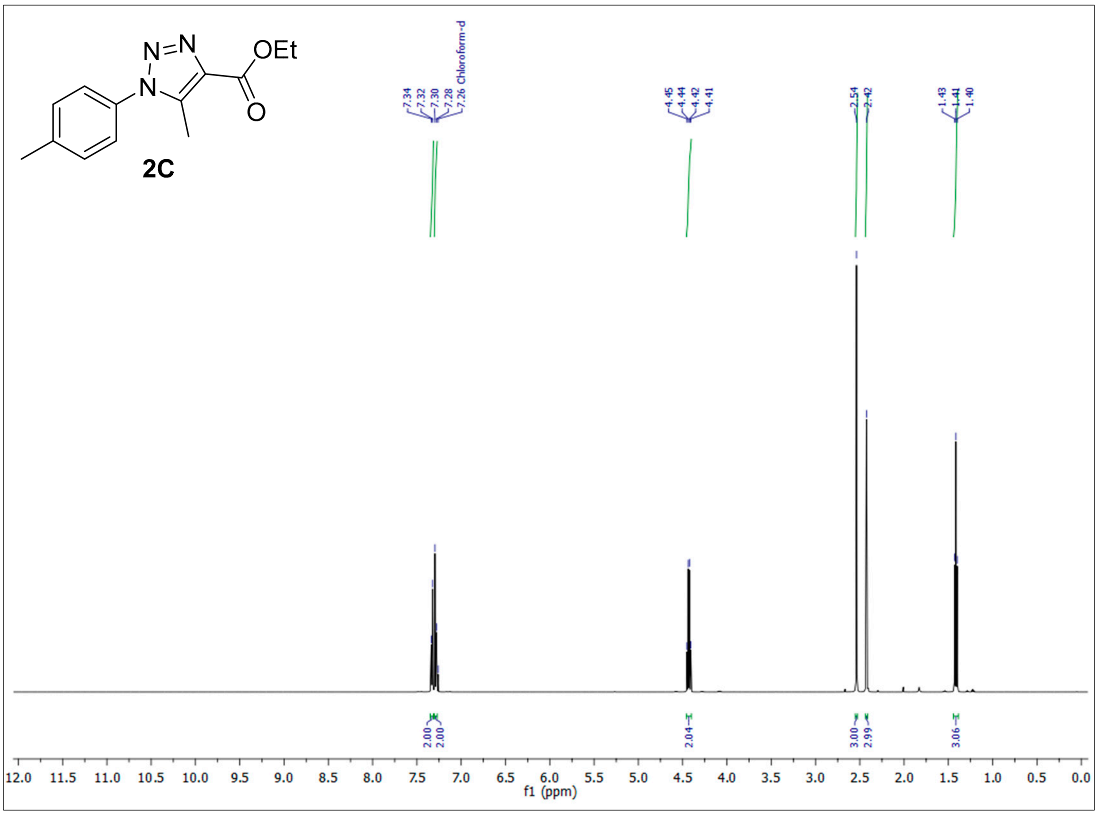

Figure S9. ^{1}H NMR (500 MHz, $CDCl_3$) spectrum of ethyl 5-methyl-1-(*p*-tolyl)-1*H*-1,2,3-triazole-4-carboxylate (**2C**).

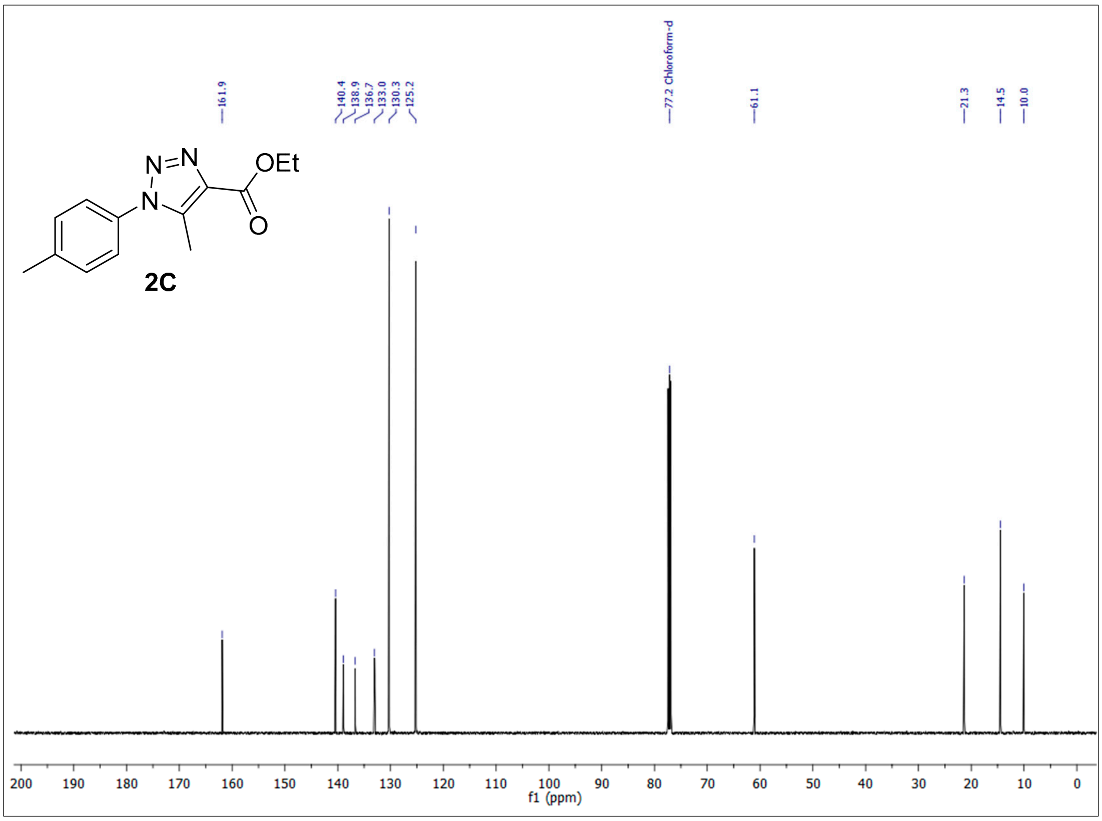

Figure S10. ^{13}C NMR (126 MHz, $CDCl_3$) spectrum of ethyl 5-methyl-1-(*p*-tolyl)-1*H*-1,2,3-triazole-4-carboxylate (**2C**).

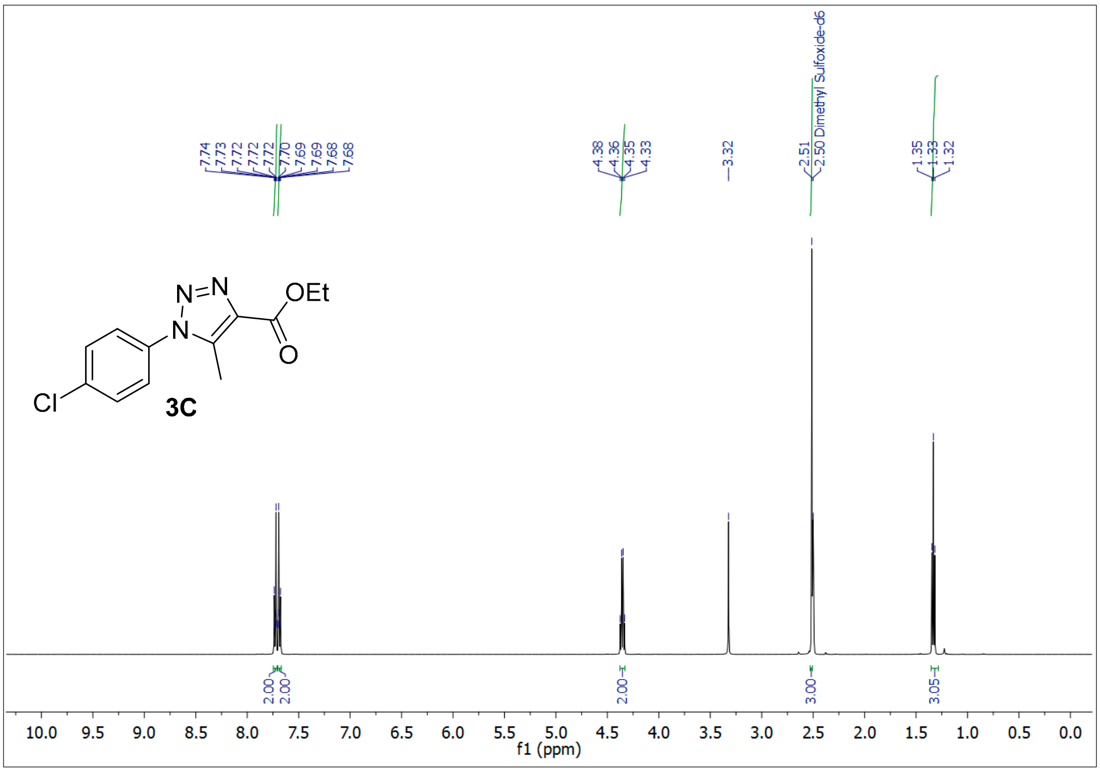

Figure S11. ^{1}H NMR (500 MHz, DMSO-d_6) spectrum of ethyl 1-(4-chlorophenyl)-5-methyl-1*H*-1,2,3-triazole-4-carboxylate (**3C**).

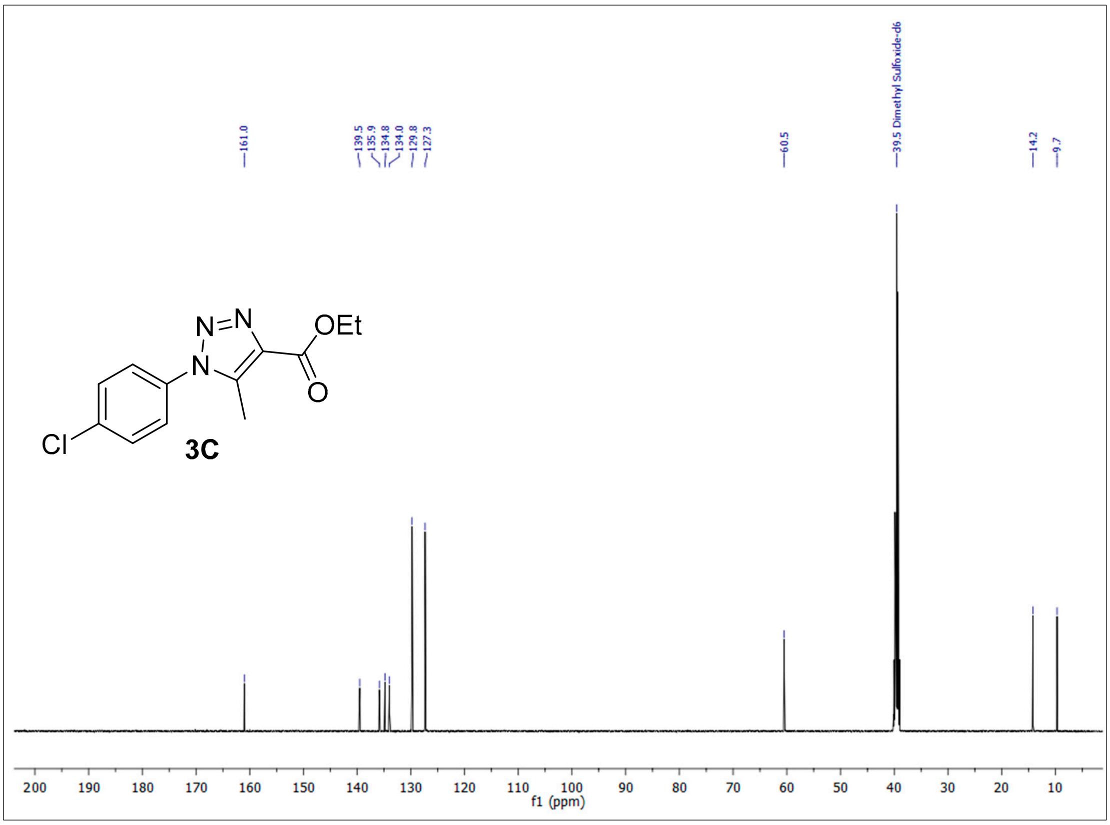

Figure S12. ^{13}C NMR (126 MHz, DMSO-d_6) spectrum of ethyl 1-(4-chlorophenyl)-5-methyl-1*H*-1,2,3-triazole-4-carboxylate (**3C**).

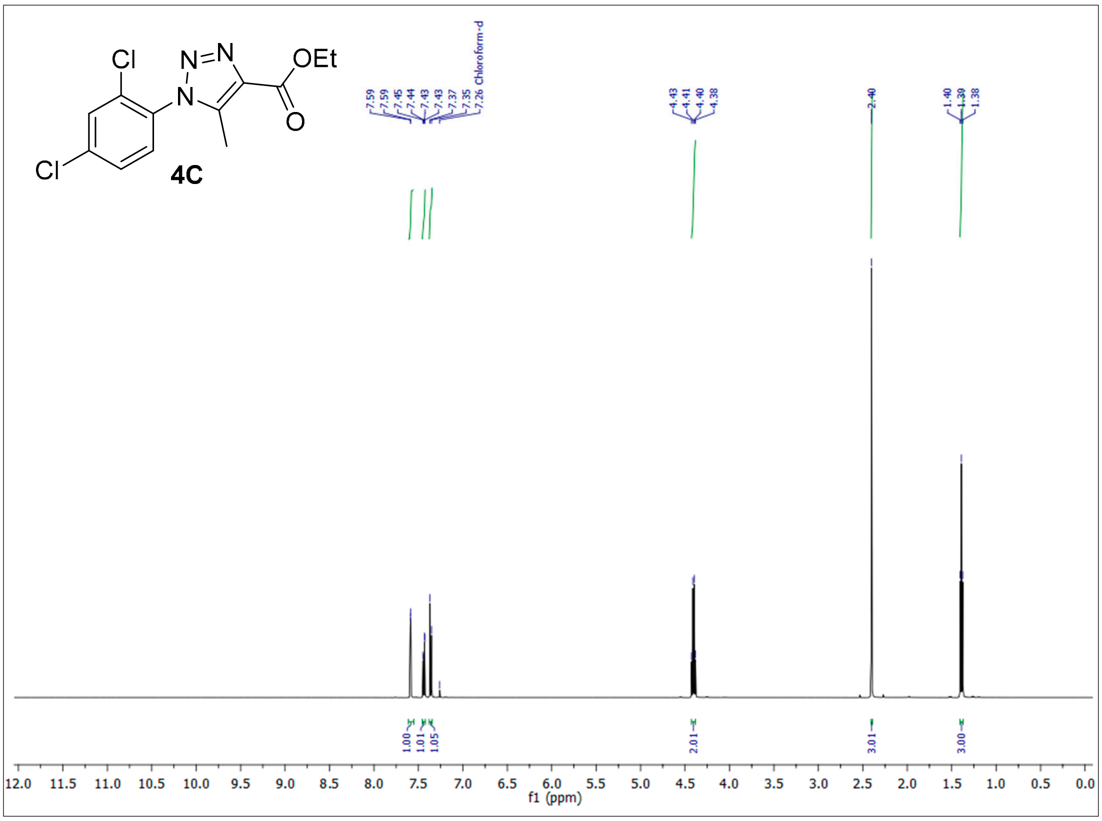

Figure S13. ^1H NMR (500 MHz, $CDCl_3$) spectrum of ethyl 1-(2,4-dichlorophenyl)-5-methyl-1*H*-1,2,3-triazole-4-carboxylate (**4C**).

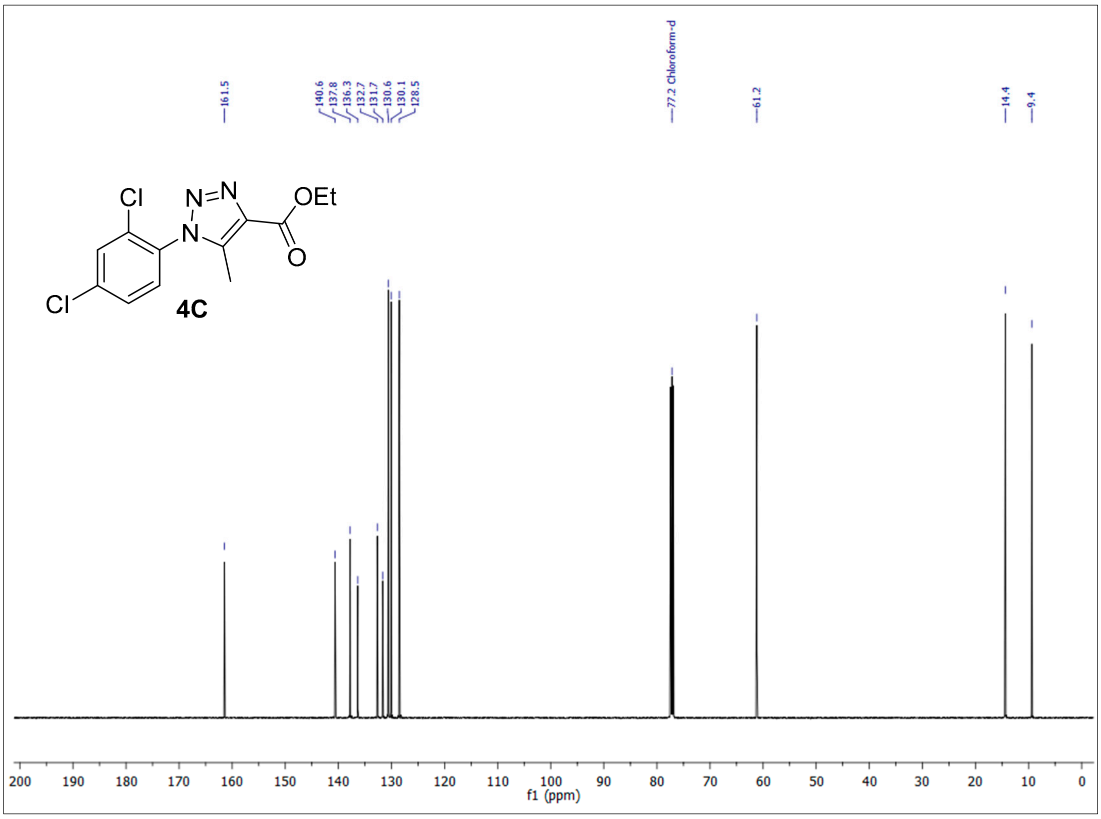

Figure S14. ^{13}C NMR (126 MHz, $CDCl_3$) spectrum of ethyl 1-(2,4-dichlorophenyl)-5-methyl-1*H*-1,2,3-triazole-4-carboxylate (**4C**).

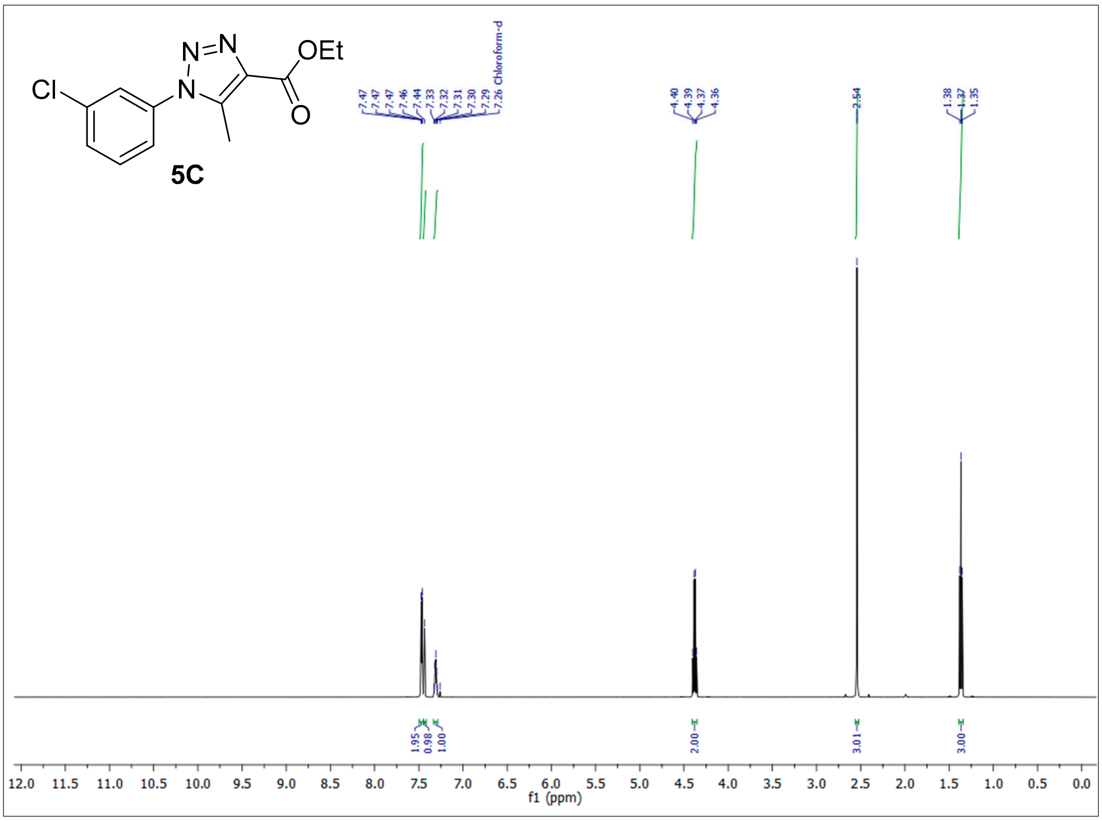

Figure S15. ^{1}H NMR (500 MHz, $CDCl_3$) spectrum of ethyl 1-(3-chlorophenyl)-5-methyl-1*H*-1,2,3-triazole-4-carboxylate (**5C**).

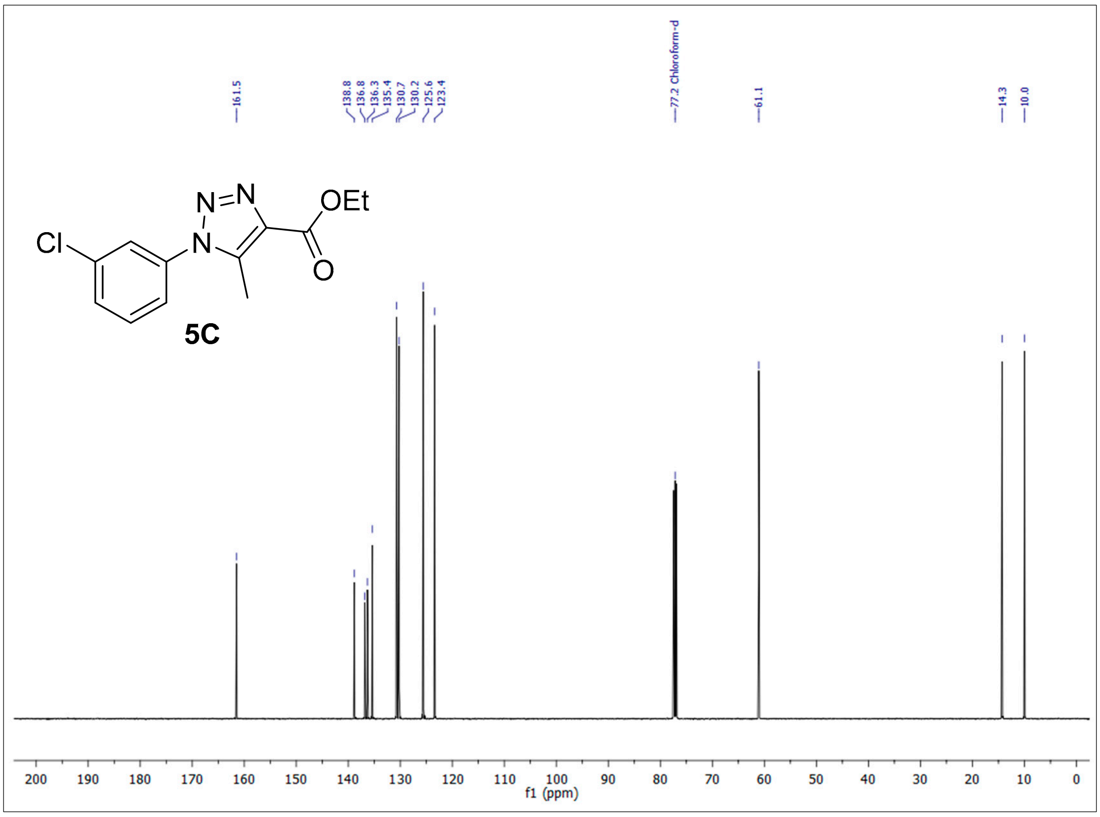

Figure S16. ^{13}C NMR (126 MHz, $CDCl_3$) spectrum of ethyl 1-(3-chlorophenyl)-5-methyl-1*H*-1,2,3-triazole-4-carboxylate (**5C**).

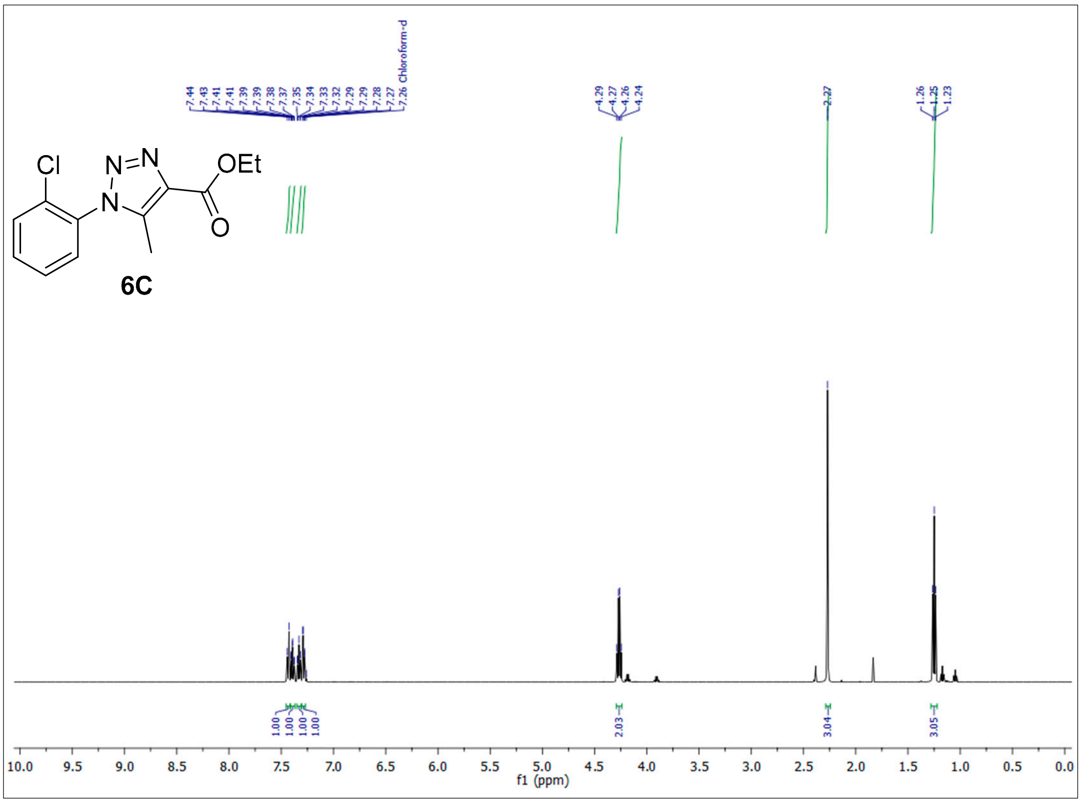

Figure S17. ^{1}H NMR (500 MHz, $CDCl_3$) spectrum of ethyl 1-(2-chlorophenyl)-5-methyl-1*H*-1,2,3-triazole-4-carboxylate (**6C**).

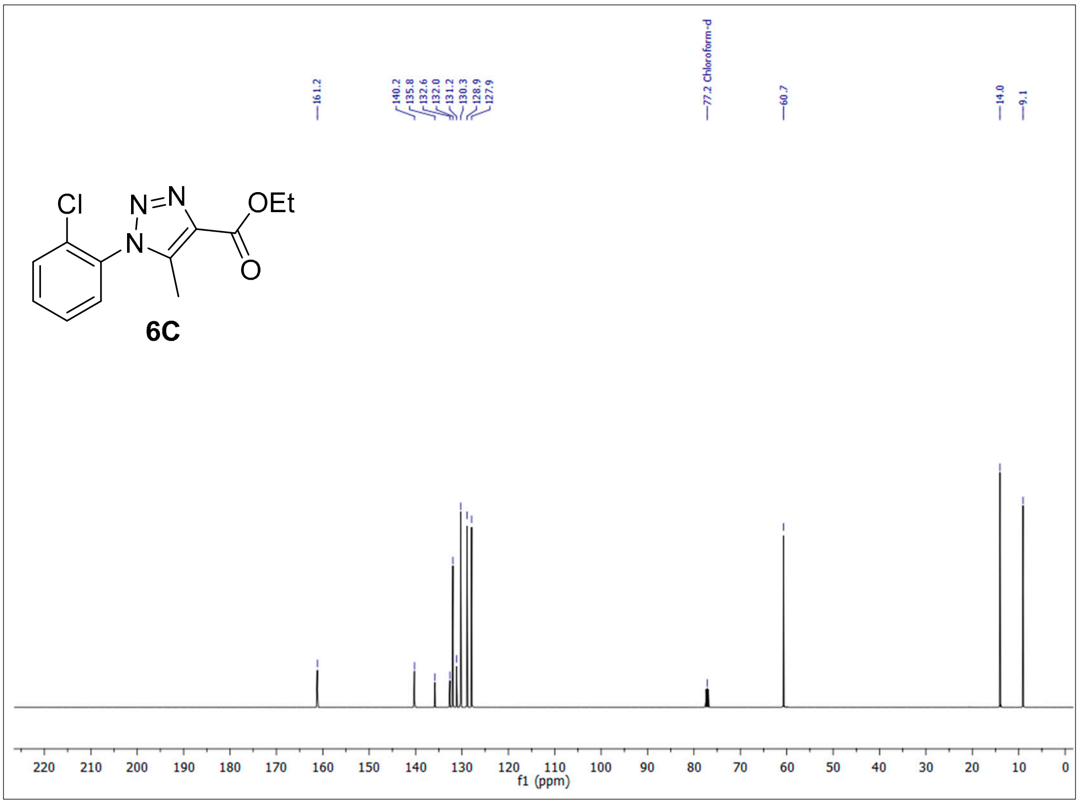

Figure S18. ^{13}C NMR (126 MHz, $CDCl_3$) spectrum of ethyl 1-(2-chlorophenyl)-5-methyl-1*H*-1,2,3-triazole-4-carboxylate (**6C**).

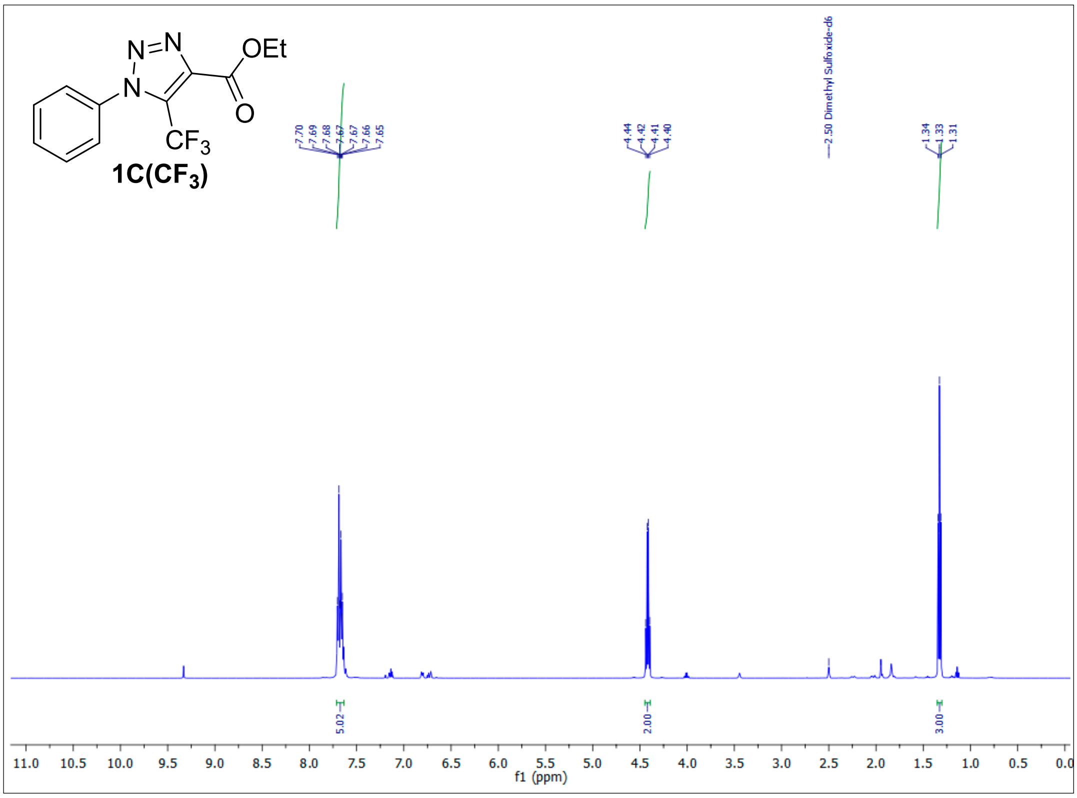

Figure S19. ^{1}H NMR (500 MHz, DMSO-d_6) spectrum of ethyl 1-phenyl-5-(trifluoromethyl)-1*H*-1,2,3-triazole-4-carboxylate [**1C(CF$_3$)**].

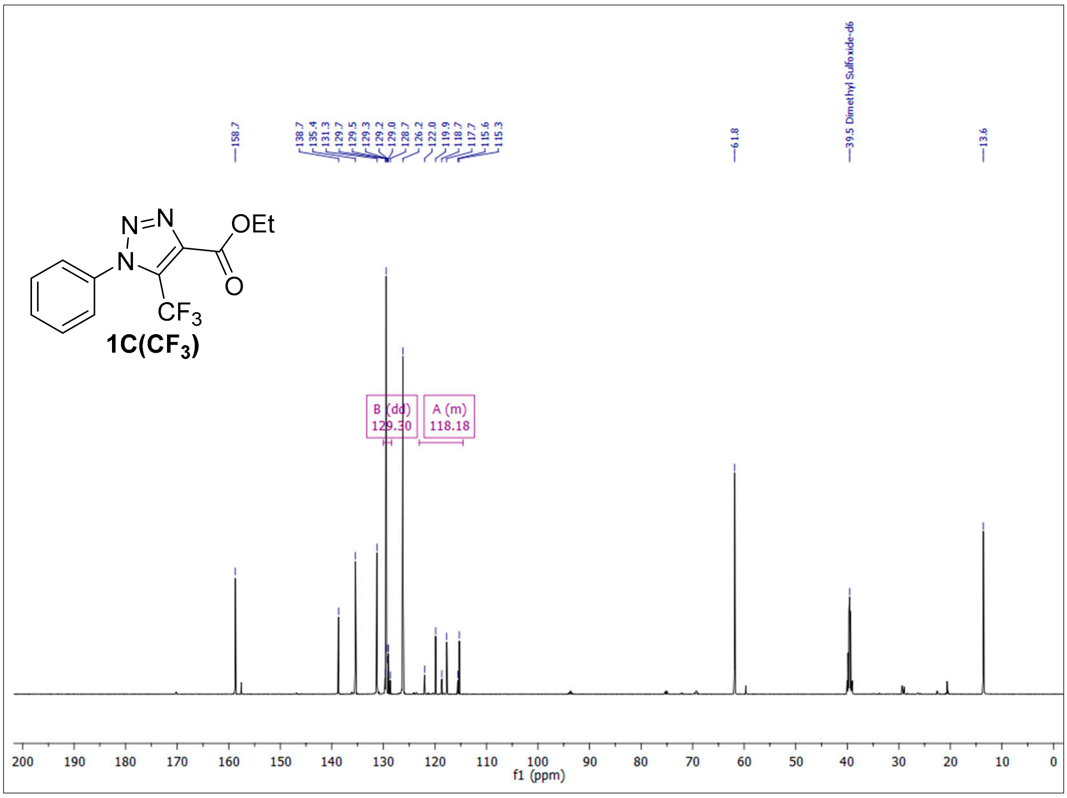

Figure S20. ^{13}C NMR (126 MHz, DMSO-d_6) spectrum of ethyl 1-phenyl-5-(trifluoromethyl)-1*H*-1,2,3-triazole-4-carboxylate [**1C(CF$_3$)**].

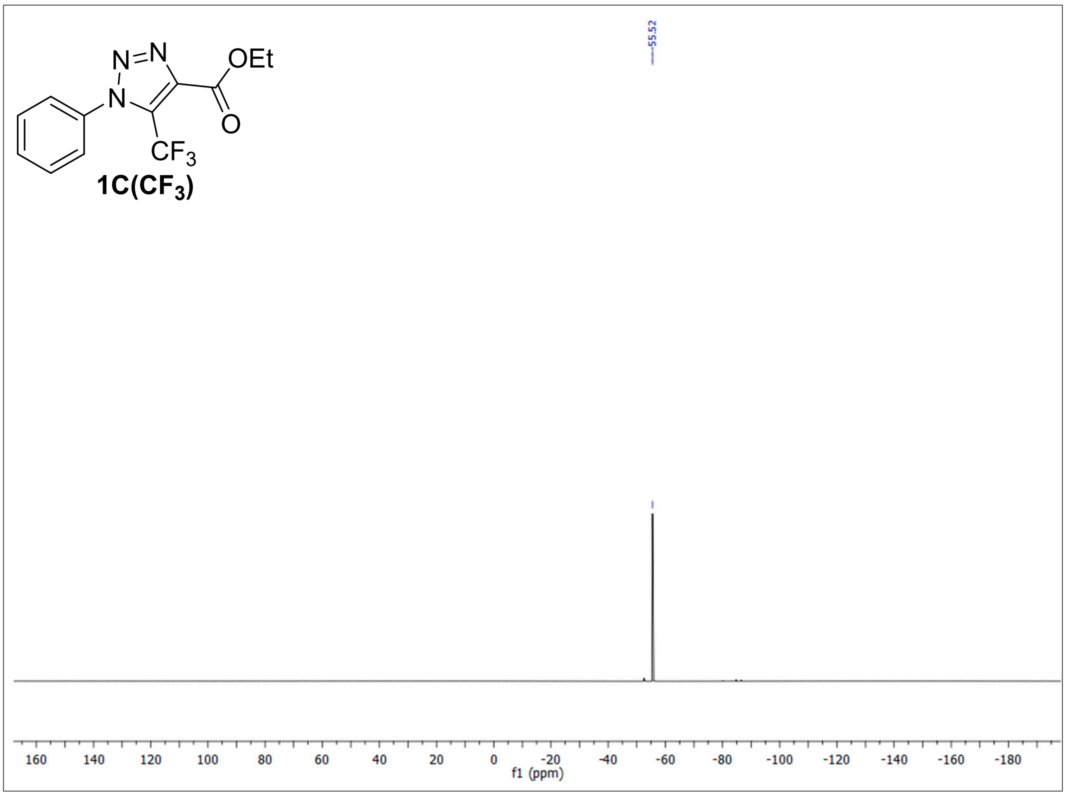

Figure S21. ^{19}F NMR (471 MHz, DMSO-d_6) spectrum of ethyl 1-phenyl-5-(trifluoromethyl)-1*H*-1,2,3-triazole-4-carboxylate [**1C(CF₃)**].

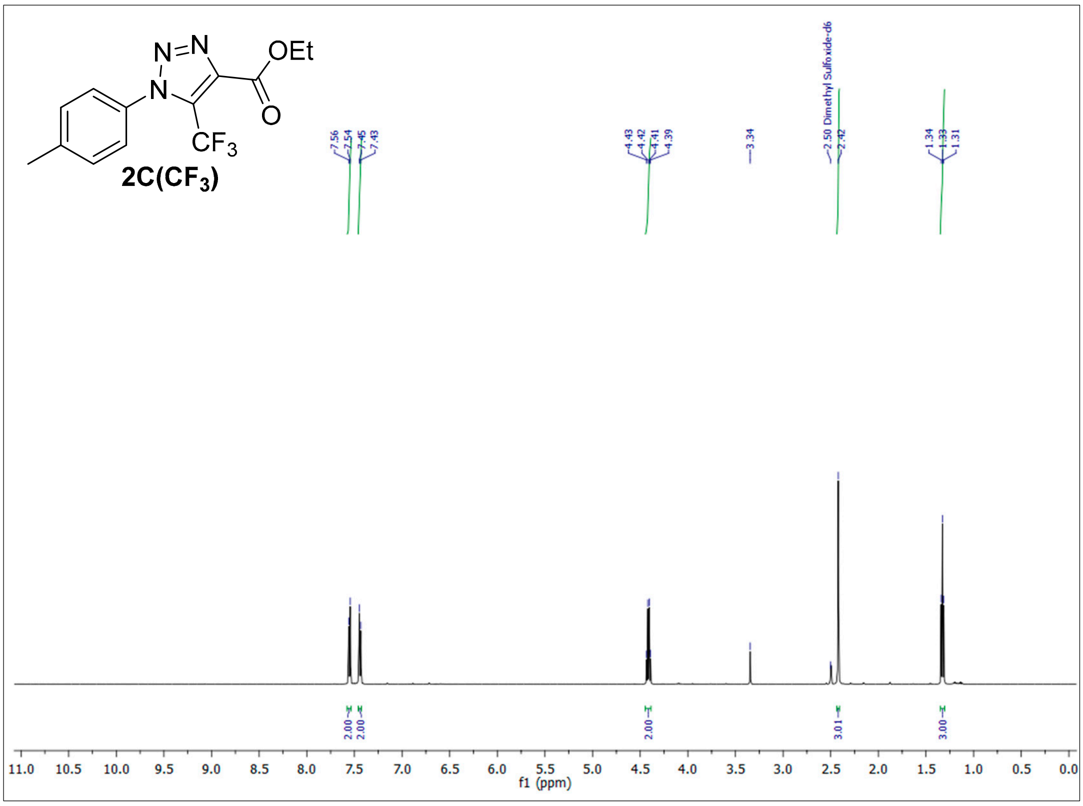

Figure S22. ^{1}H NMR (500 MHz, DMSO-d_6) spectrum of ethyl 1-(p-tolyl)-5-(trifluoromethyl)-1*H*-1,2,3-triazole-4-carboxylate [**2C(CF₃)**].

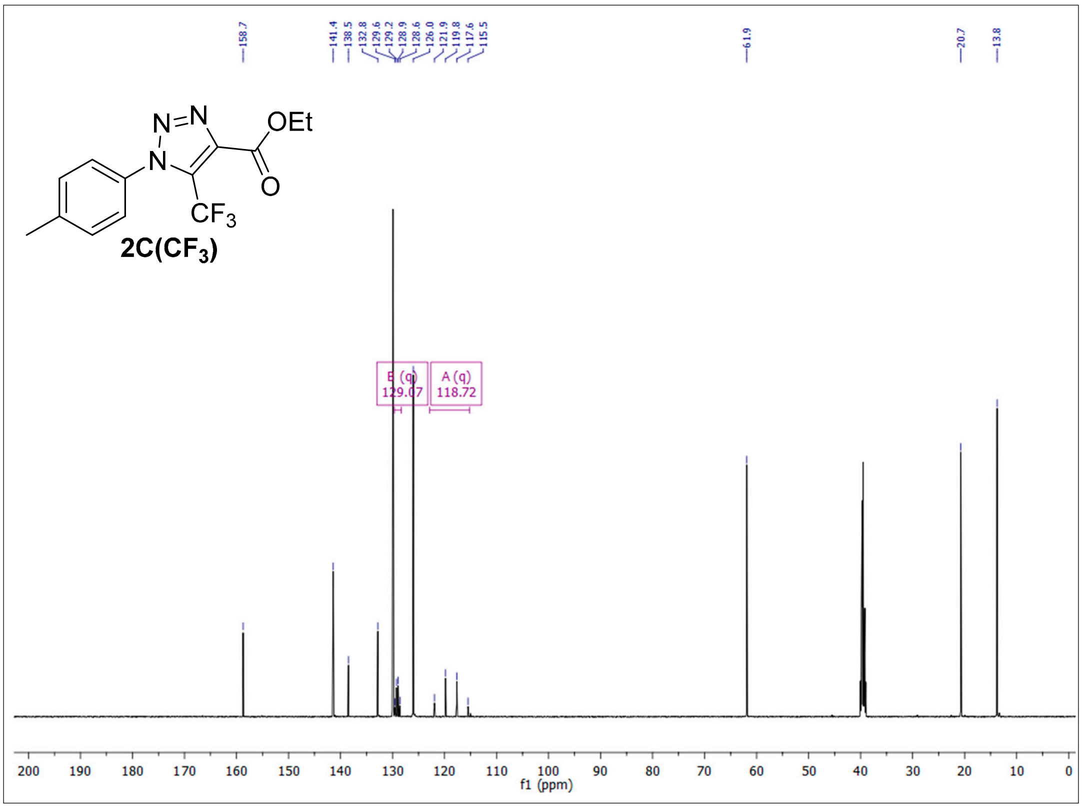

Figure S23. ^{13}C NMR (126 MHz, DMSO-d$_6$) spectrum of ethyl 1-(p-tolyl)-5-(trifluoromethyl)-1*H*-1,2,3-triazole-4-carboxylate [**2C(CF$_3$)**].

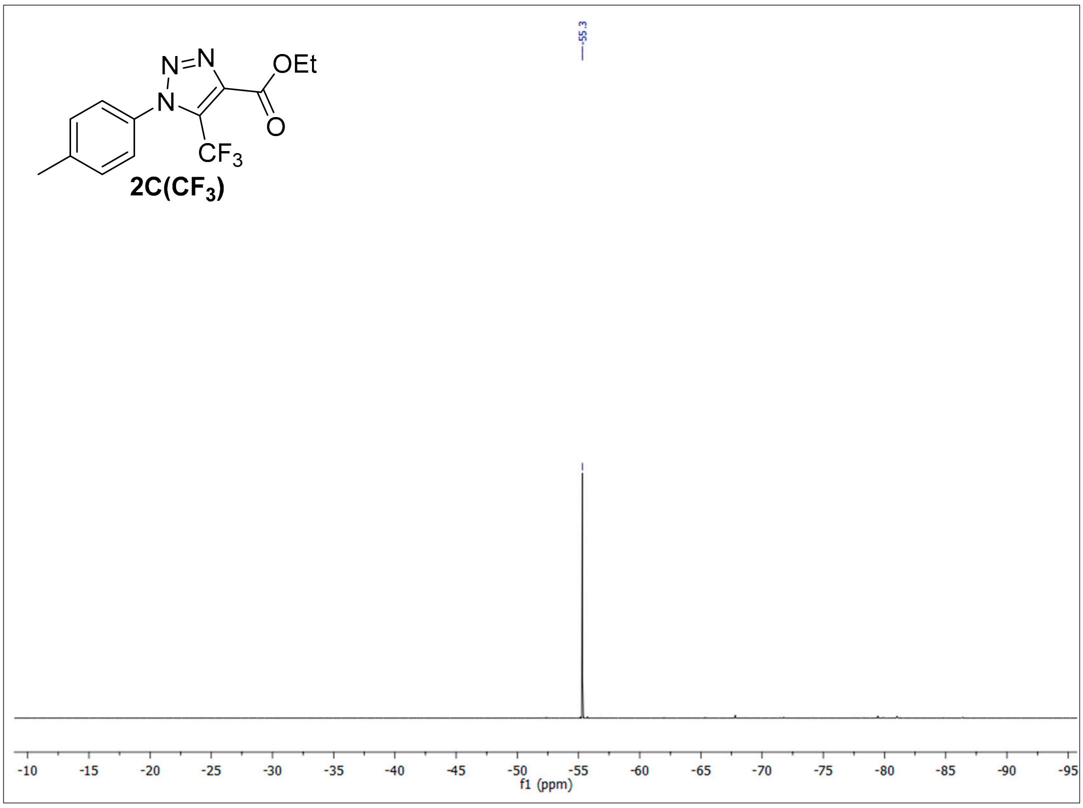

Figure S24. ^{19}F NMR (476 MHz, DMSO-d$_6$) spectrum of ethyl 1-(p-tolyl)-5-(trifluoromethyl)-1*H*-1,2,3-triazole-4-carboxylate [**2C(CF$_3$)**].

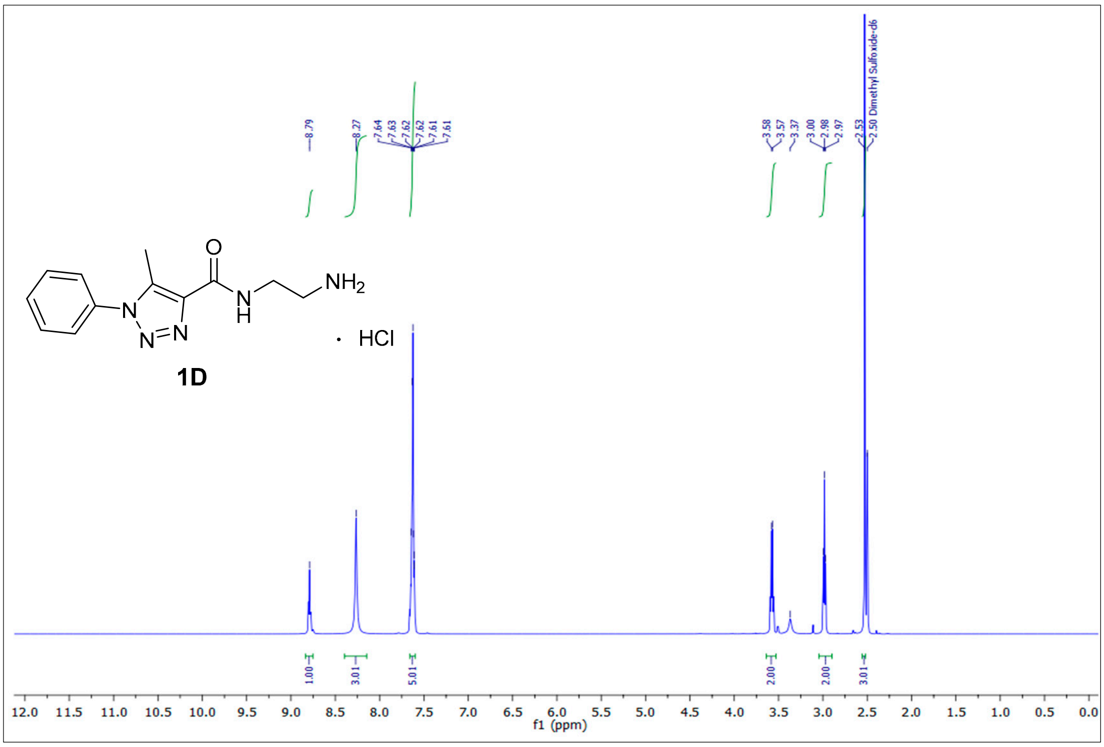

Figure S25. ^{1}H NMR (500 MHz, DMSO-d_6) spectrum of *N*-(2-aminoethyl)-5-methyl-1-phenyl-1*H*-1,2,3-triazole-4-carboxamide hydrochloride (**1D**).

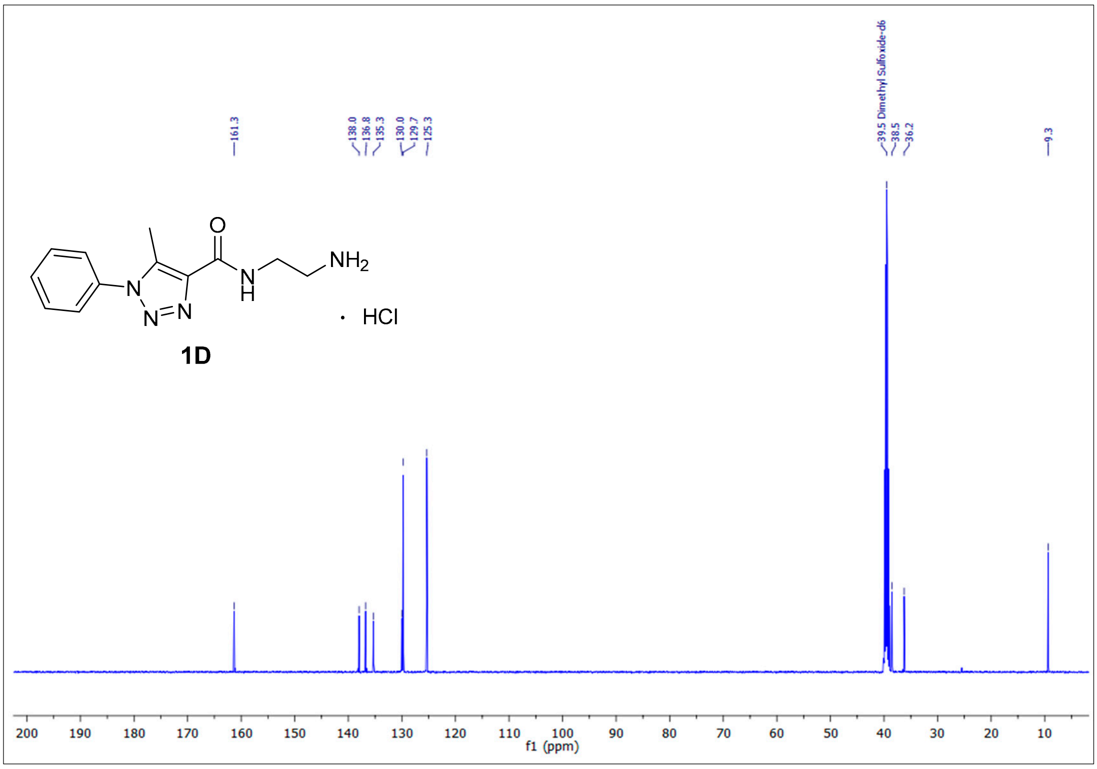

Figure S26. ^{13}C NMR (126 MHz, DMSO-d_6) spectrum of *N*-(2-aminoethyl)-5-methyl-1-phenyl-1*H*-1,2,3-triazole-4-carboxamide hydrochloride (**1D**).

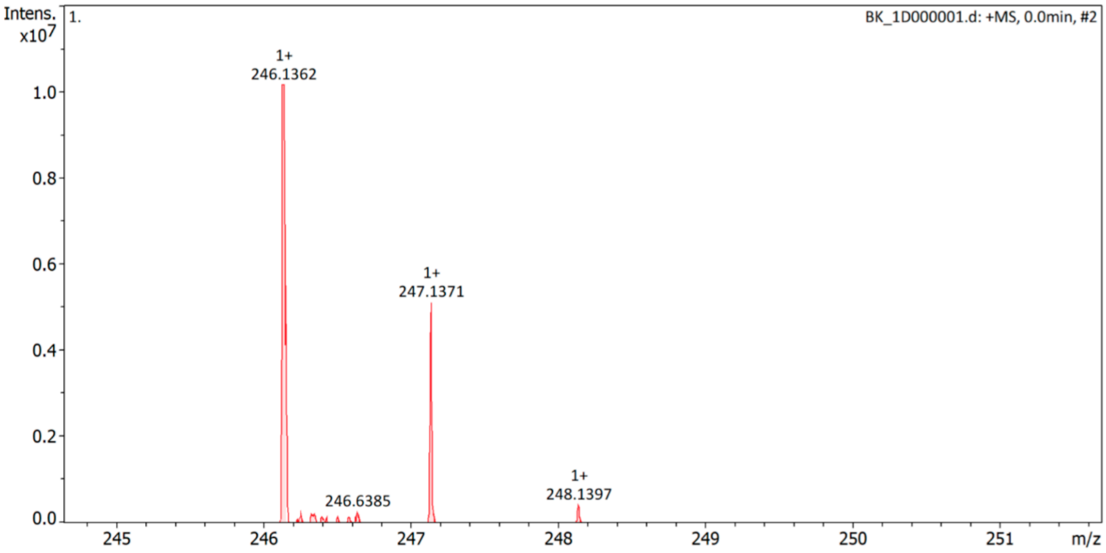

Figure S27. HRMS (ESI) spectrum of *N*-(2-aminoethyl)-5-methyl-1-phenyl-1*H*-1,2,3-triazole-4-carboxamide hydrochloride (**1D**).

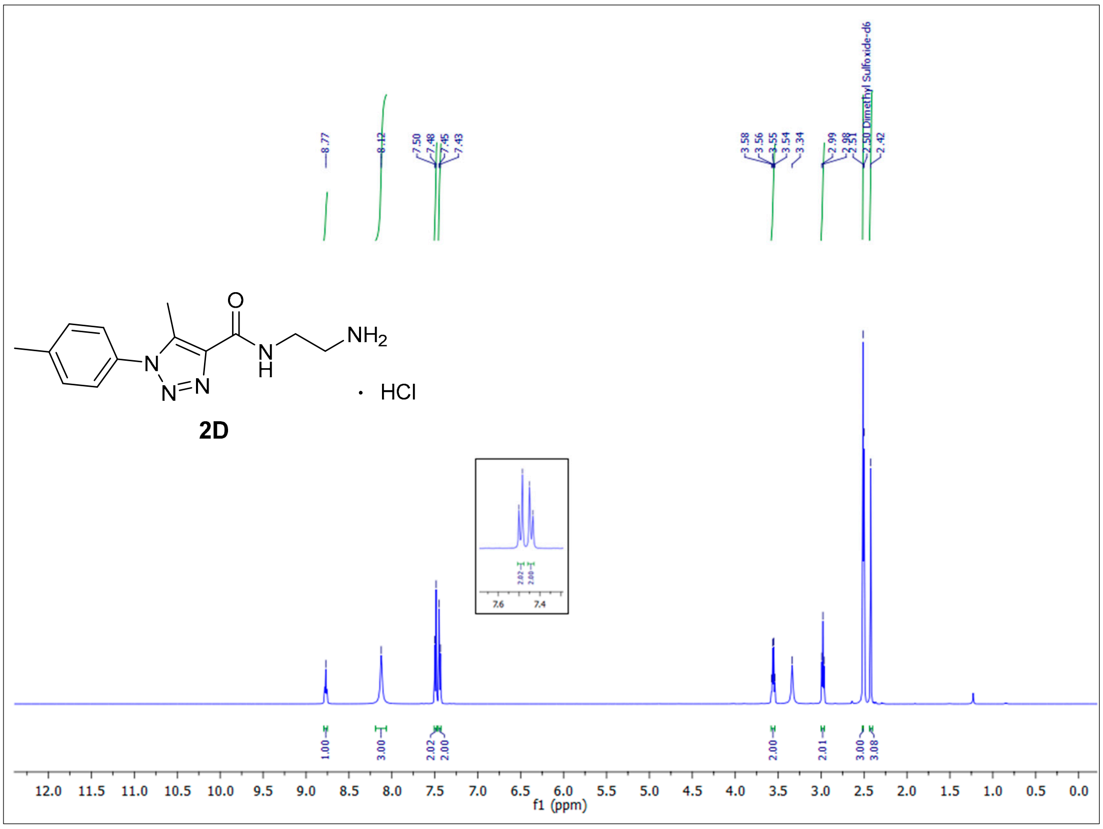

Figure S28. ^{1}H NMR (500 MHz, DMSO-d6) spectrum of *N*-(2-aminoethyl)-5-methyl-1-(*p*-tolyl)-1*H*-1,2,3-triazole-4-carboxamide hydrochloride (**2D**).

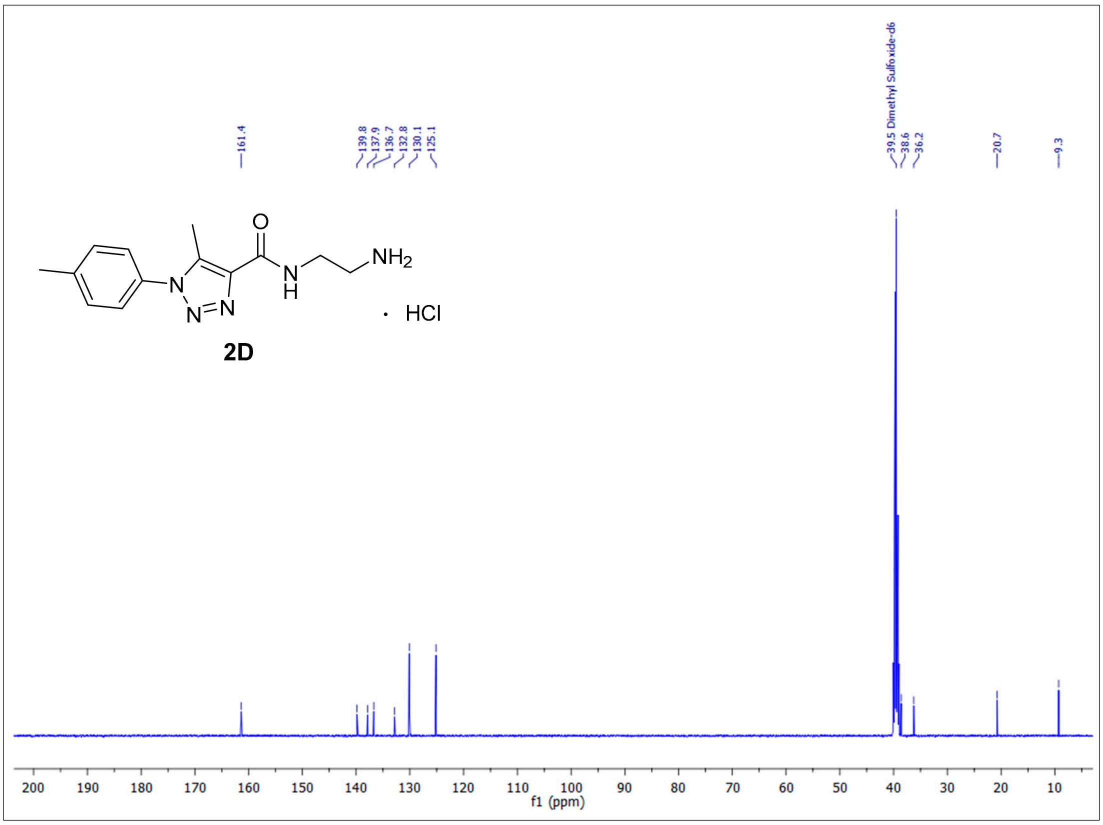

Figure S29. ^{13}C NMR (126 MHz, DMSO-d_6) spectrum of *N*-(2-aminoethyl)-5-methyl-1-(*p*-tolyl)-1*H*-1,2,3-triazole-4-carboxamide hydrochloride (**2D**).

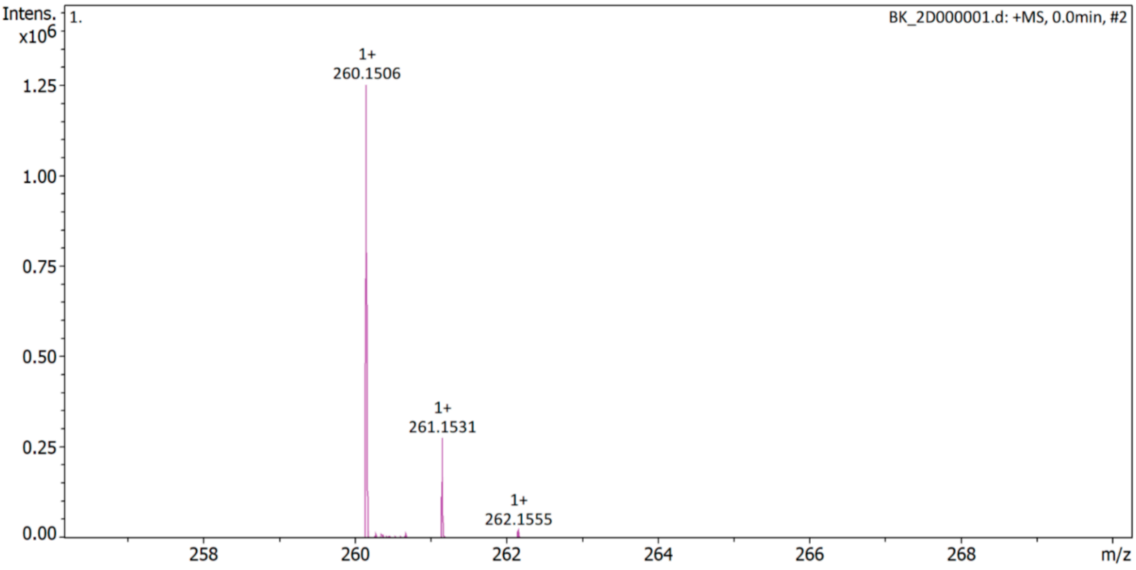

Figure S30. HRMS (ESI) spectrum of *N*-(2-aminoethyl)-5-methyl-1-(*p*-tolyl)-1*H*-1,2,3-triazole-4-carboxamide hydrochloride (**2D**).

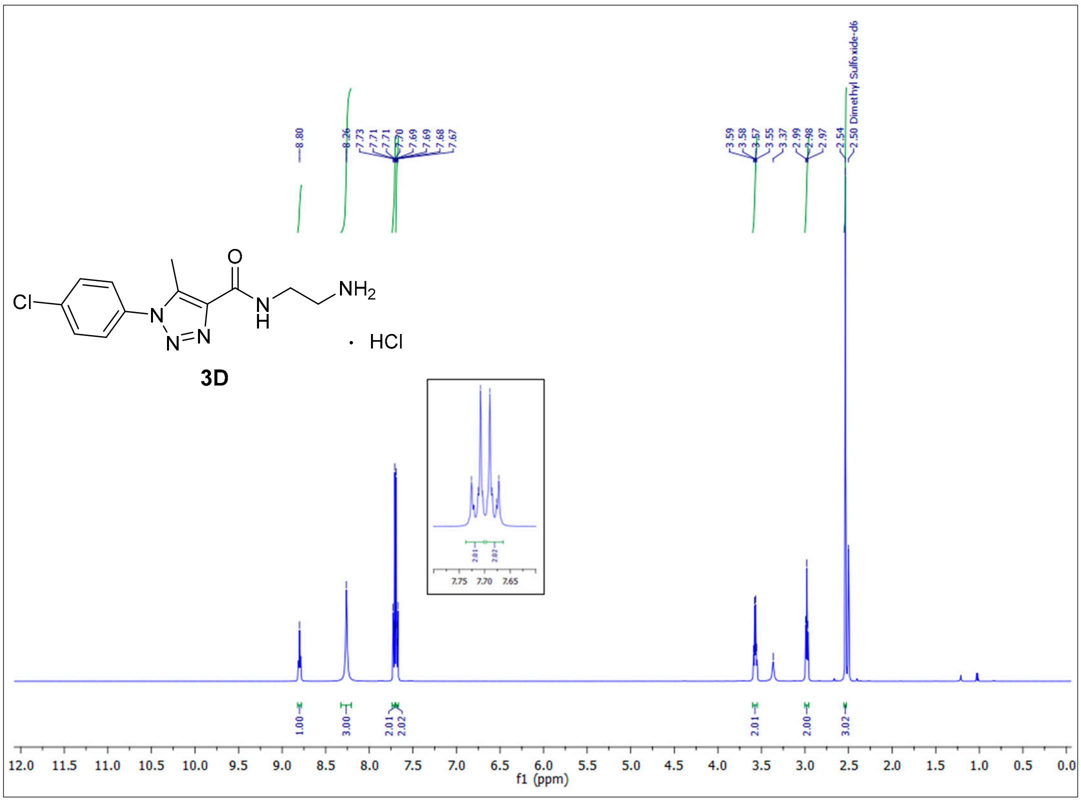

Figure S31. ^{1}H NMR (500 MHz, DMSO-d_6) spectrum of *N*-(2-aminoethyl)-1-(4-chlorophenyl)-5-methyl-1*H*-1,2,3-triazole-4-carboxamide hydrochloride (**3D**).

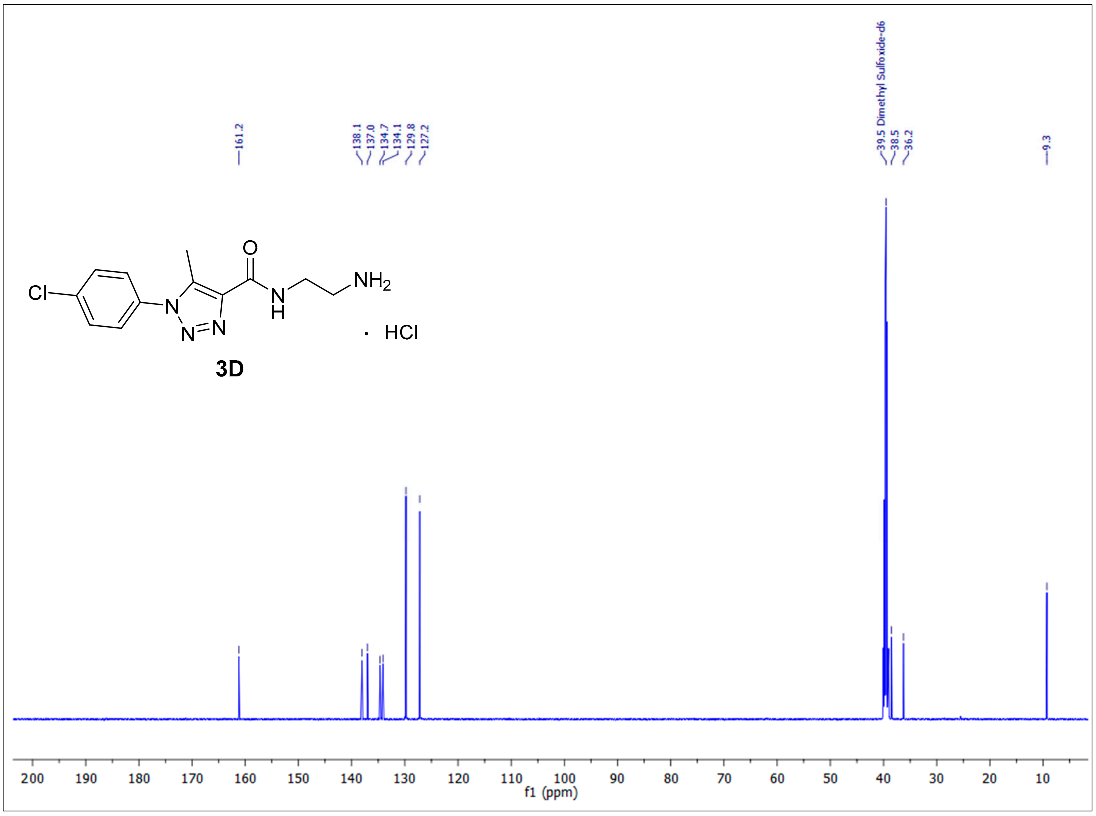

Figure S32. ^{13}C NMR (126 MHz, DMSO-d_6) spectrum of *N*-(2-aminoethyl)-1-(4-chlorophenyl)-5-methyl-1*H*-1,2,3-triazole-4-carboxamide hydrochloride (**3D**).

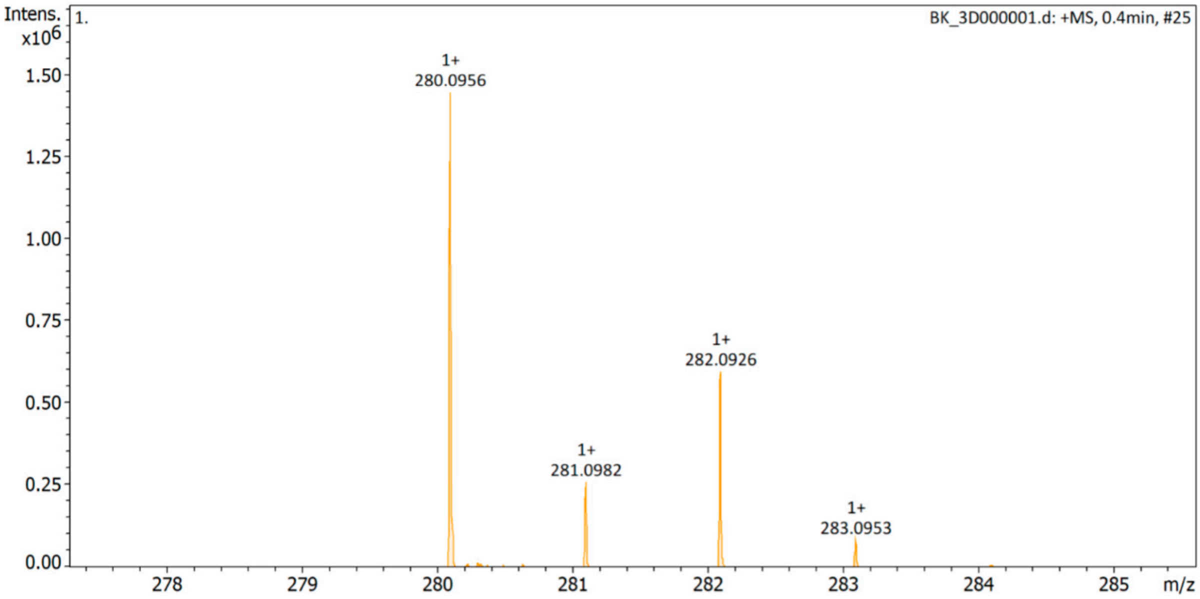

Figure S33. HRMS (ESI) spectrum of *N*-(2-aminoethyl)-1-(4-chlorophenyl)-5-methyl-1*H*-1,2,3-triazole-4-carboxamide hydrochloride (**3D**).

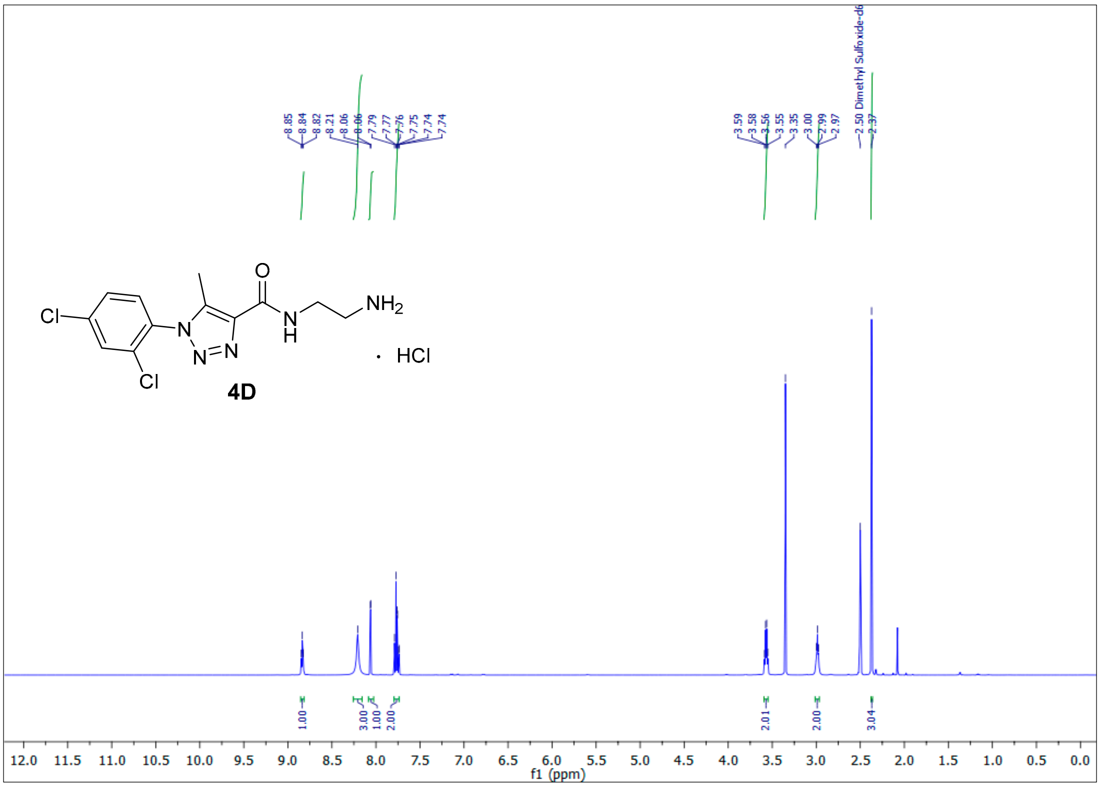

Figure S34. ^{1}H NMR (500 MHz, DMSO-d6) spectrum of *N*-(2-aminoethyl)-1-(2,4-dichlorophenyl)-5-methyl-1*H*-1,2,3-triazole-4-carboxamide hydrochloride (**4D**).

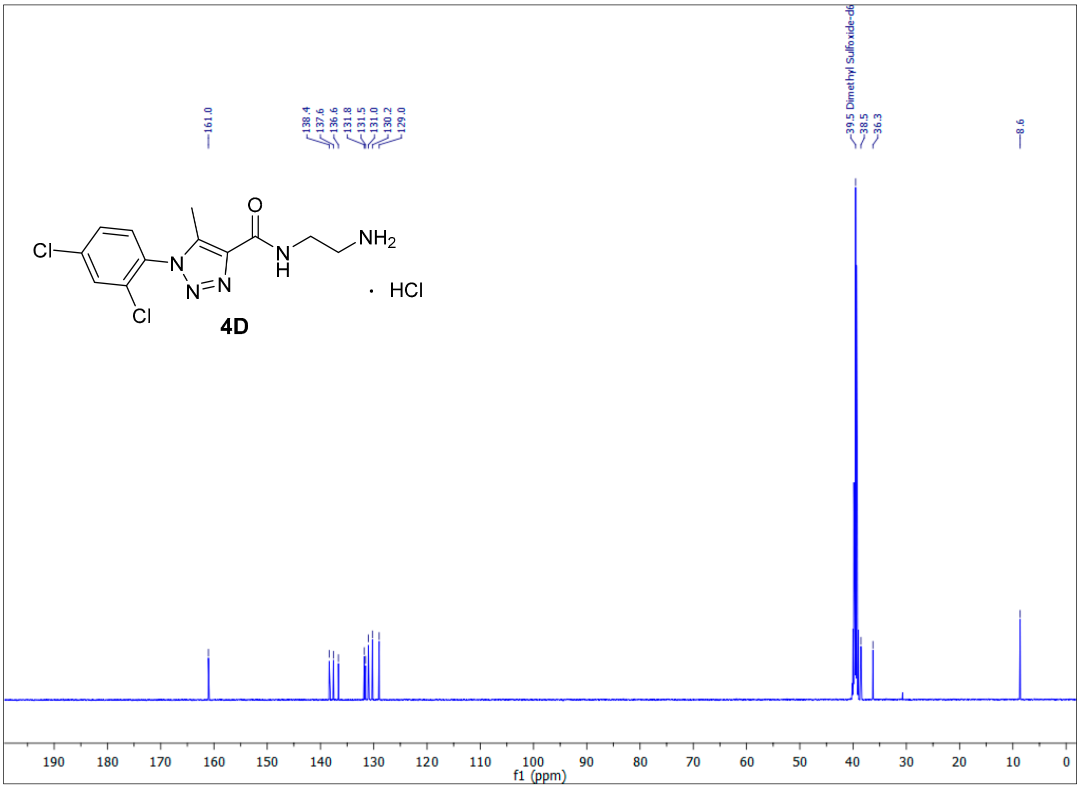

Figure S35. ^{13}C NMR (126 MHz, DMSO-d_6) spectrum of *N*-(2-aminoethyl)-1-(2,4-dichlorophenyl)-5-methyl-1*H*-1,2,3-triazole-4-carboxamide hydrochloride (**4D**).

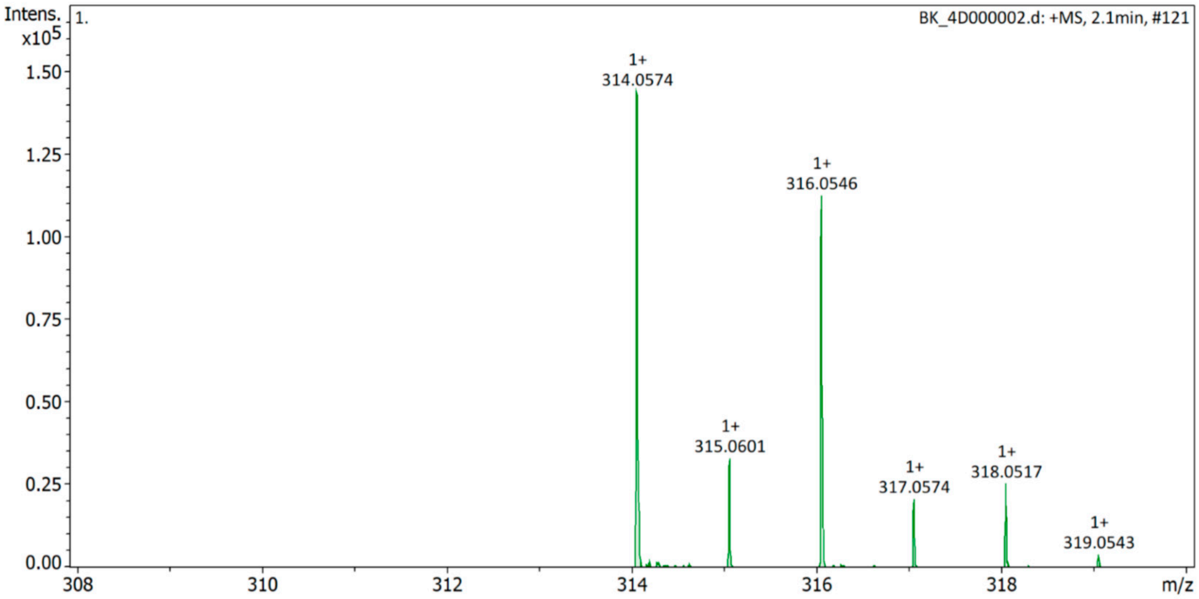

Figure S36. HRMS (ESI) spectrum of *N*-(2-aminoethyl)-1-(2,4-dichlorophenyl)-5-methyl-1*H*-1,2,3-triazole-4-carboxamide hydrochloride (**4D**).

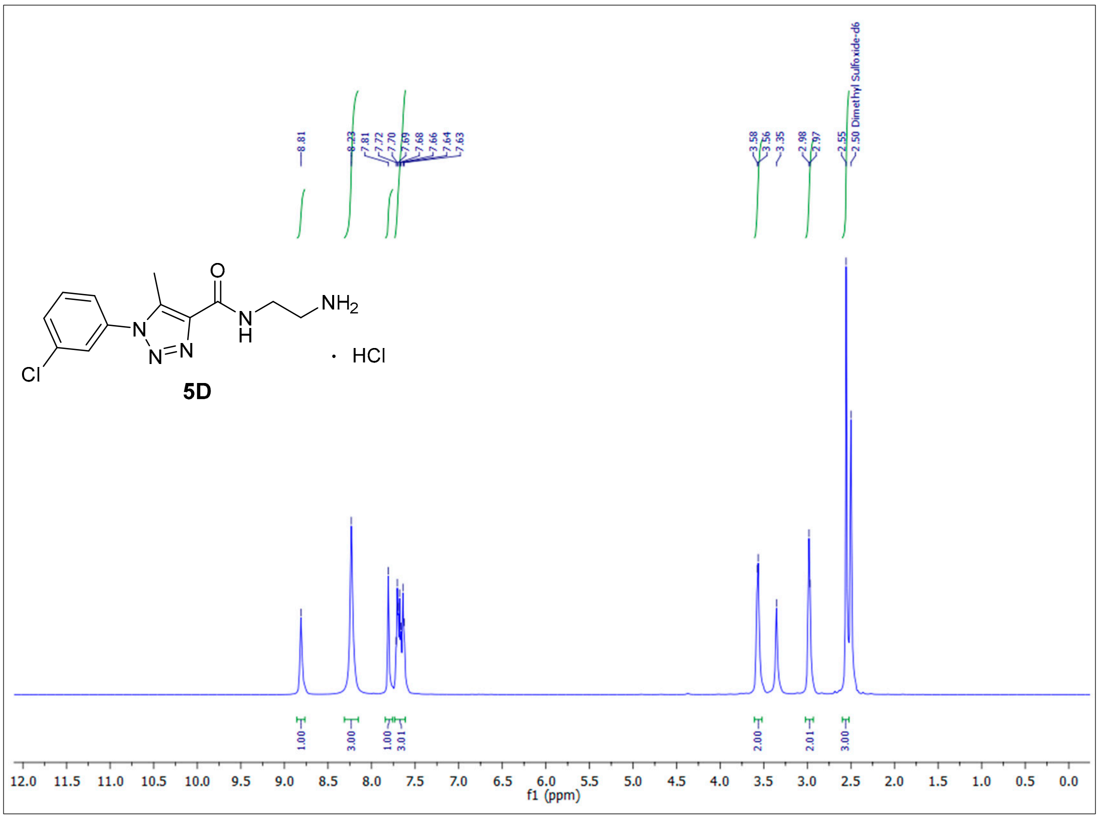

Figure S37. ^{1}H NMR (500 MHz, DMSO-d$_6$) spectrum of *N*-(2-aminoethyl)-1-(3-chlorophenyl)-5-methyl-1*H*-1,2,3-triazole-4-carboxamide hydrochloride (**5D**).

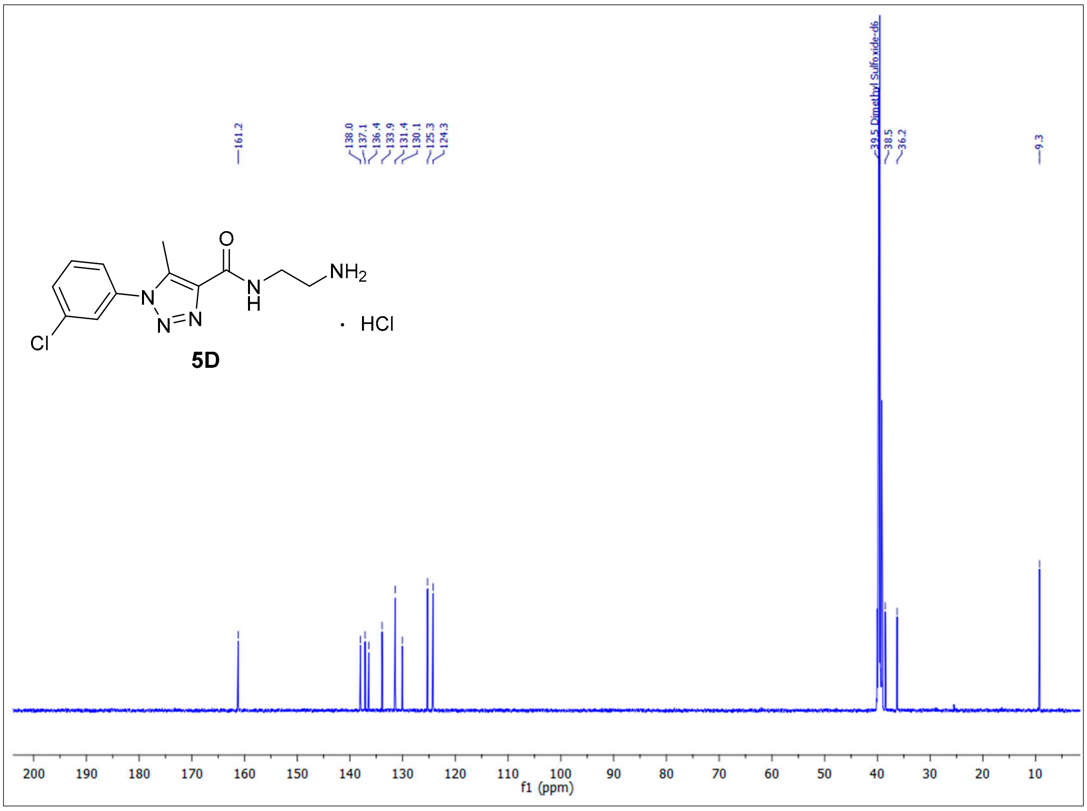

Figure S38. ^{13}C NMR (126 MHz, DMSO-d$_6$) spectrum of *N*-(2-aminoethyl)-1-(3-chlorophenyl)-5-methyl-1*H*-1,2,3-triazole-4-carboxamide hydrochloride (**5D**).

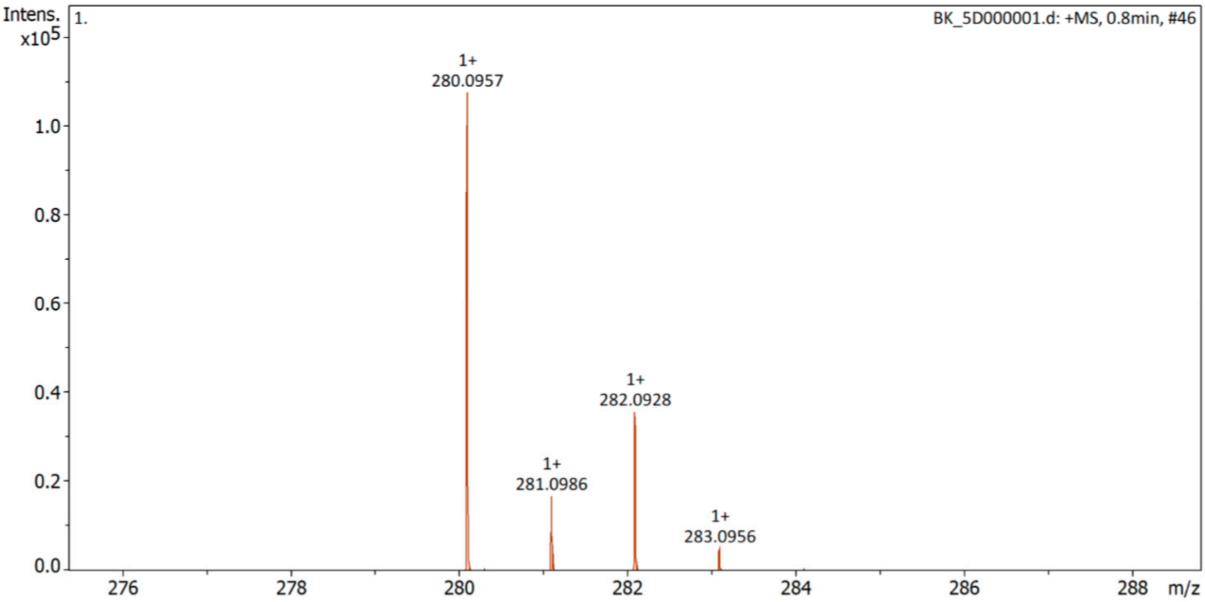

Figure S39. HRMS (ESI) spectrum of *N*-(2-aminoethyl)-1-(3-chlorophenyl)-5-methyl-1*H*-1,2,3-triazole-4-carboxamide hydrochloride (**5D**).

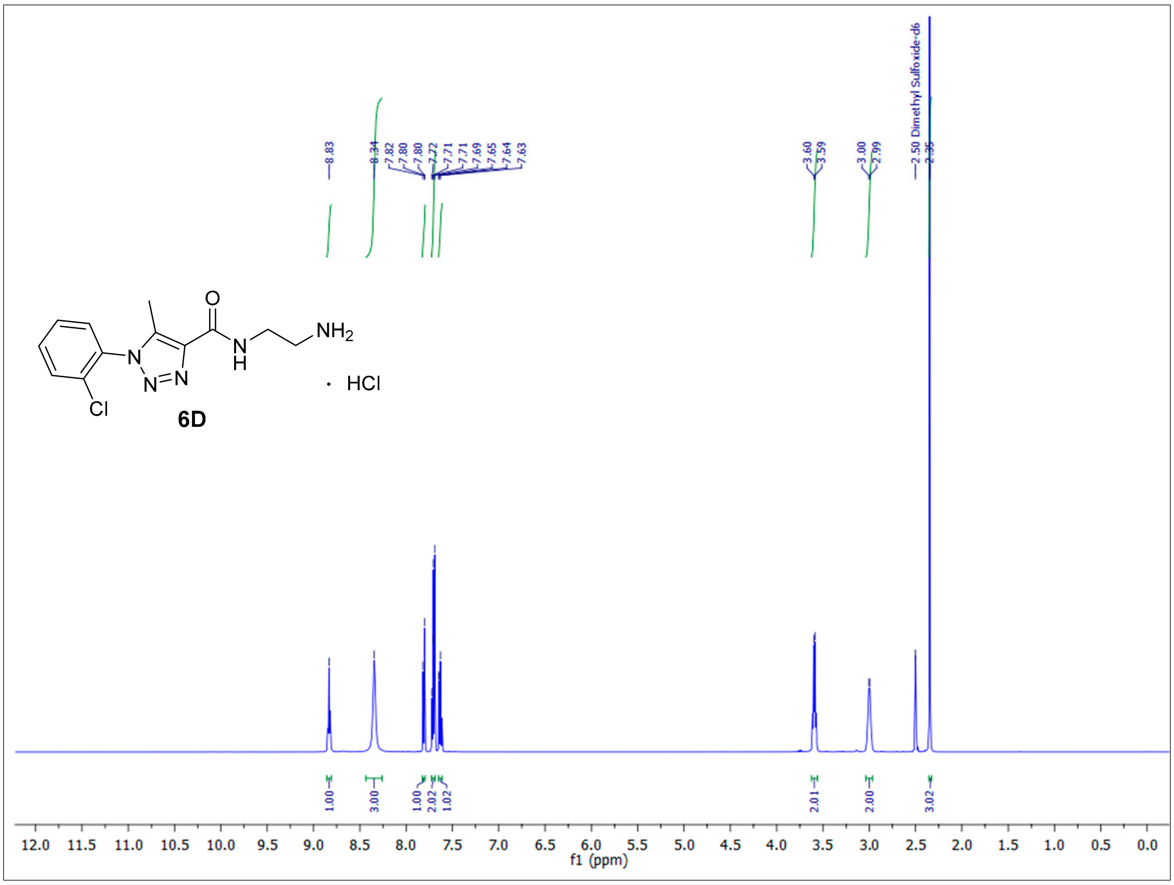

Figure S40. ^{1}H NMR (500 MHz, DMSO-d$_6$) spectrum of *N*-(2-aminoethyl)-1-(2-chlorophenyl)-5-methyl-1*H*-1,2,3-triazole-4-carboxamide hydrochloride (**6D**).

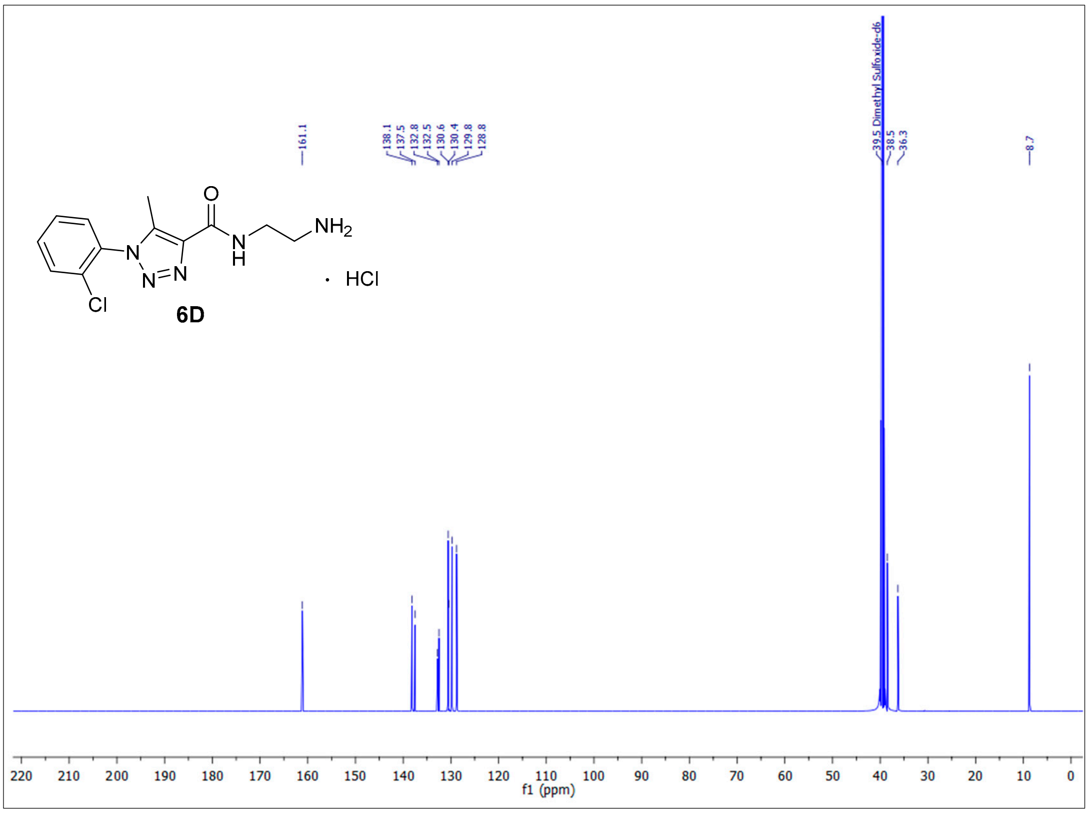

Figure S41. ^{13}C NMR (126 MHz, DMSO-d_6) spectrum of *N*-(2-aminoethyl)-1-(2-chlorophenyl)-5-methyl-1*H*-1,2,3-triazole-4-carboxamide hydrochloride (**6D**).

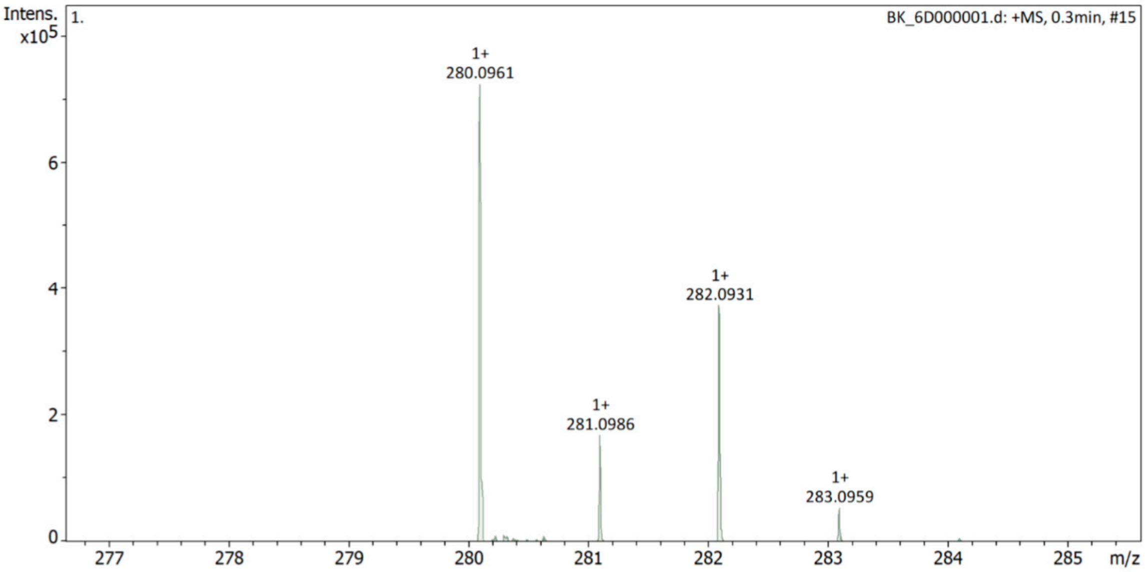

Figure S42. HRMS (ESI) spectrum of *N*-(2-aminoethyl)-1-(2-chlorophenyl)-5-methyl-1*H*-1,2,3-triazole-4-carboxamide hydrochloride (**6D**).

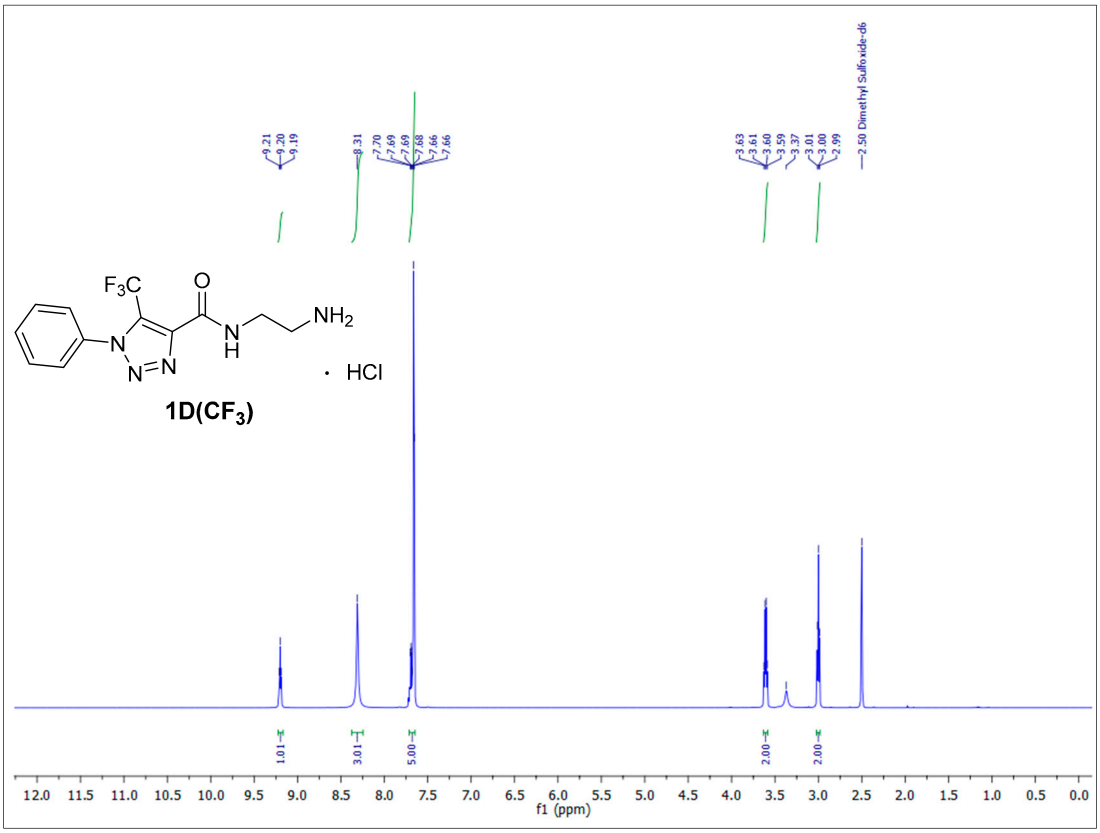

Figure S43. ^{1}H NMR (500 MHz, DMSO-d$_6$) spectrum of *N*-(2-aminoethyl)-1-phenyl-5-(trifluoromethyl)-1*H*-1,2,3-triazole-4-carboxamide hydrochloride [**1D(CF$_3$)**].

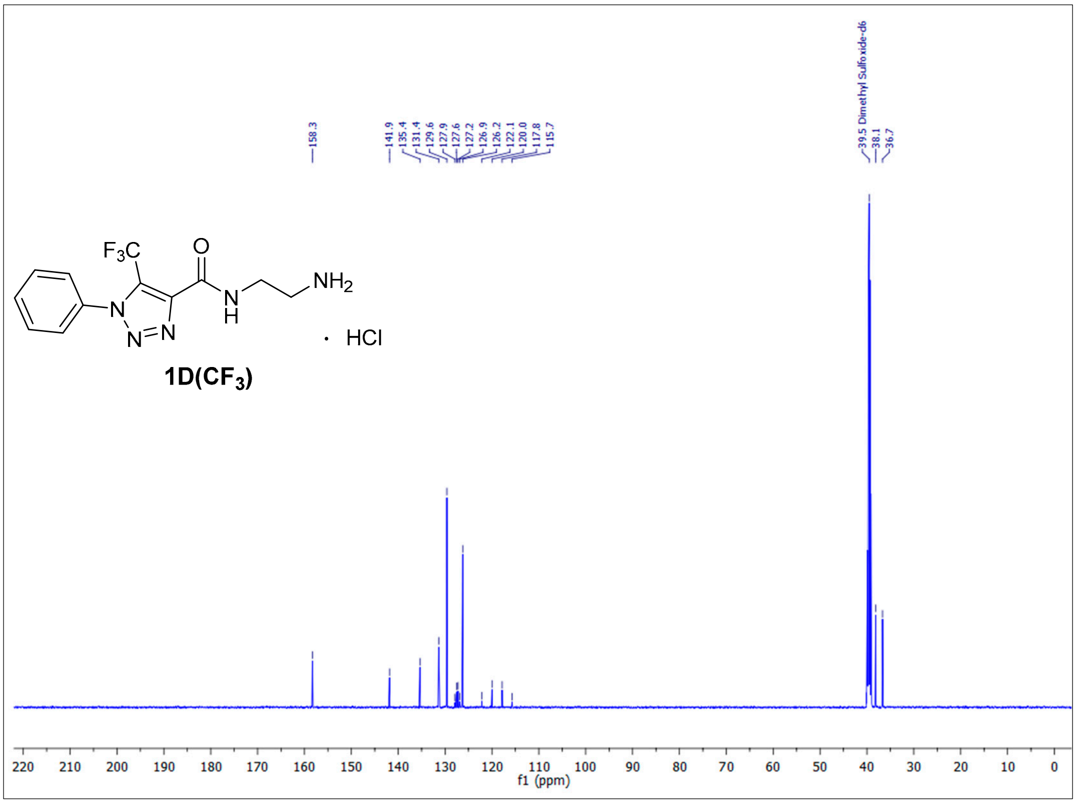

Figure S44. ^{13}C NMR (126 MHz, DMSO-d$_6$) spectrum of *N*-(2-aminoethyl)-1-phenyl-5-(trifluoromethyl)-1*H*-1,2,3-triazole-4-carboxamide hydrochloride [**1D(CF$_3$)**].

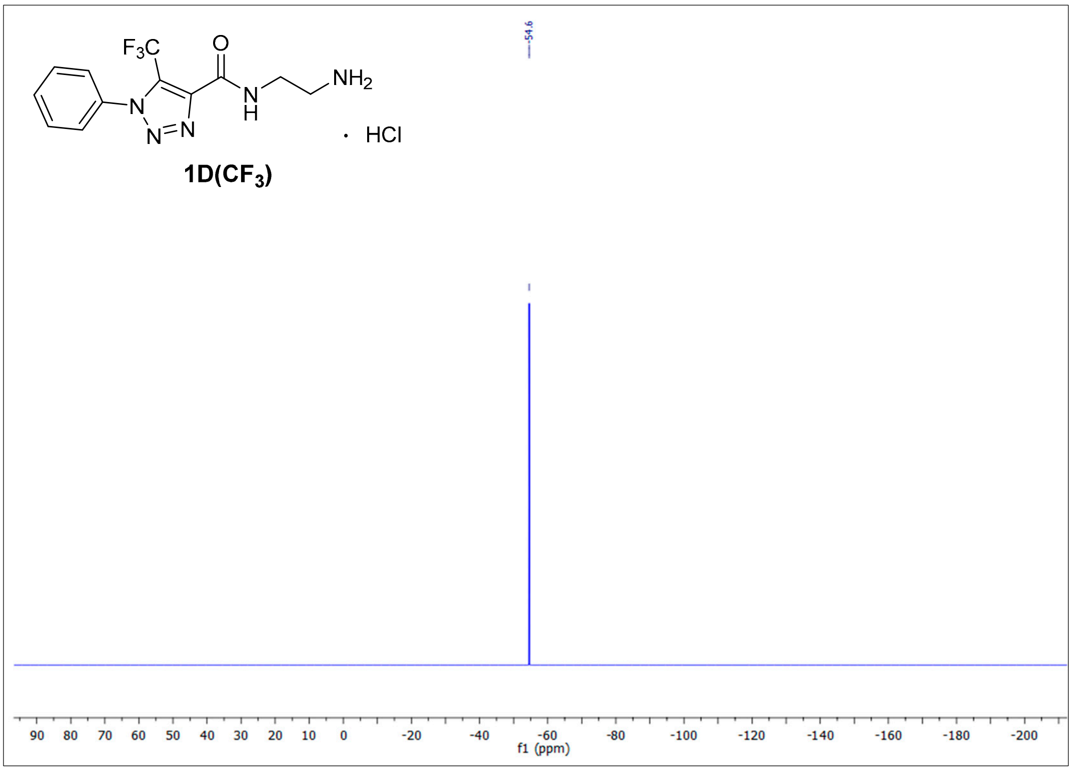

Figure S45. ^{19}F NMR (476 MHz, DMSO-d_6) spectrum of *N*-(2-aminoethyl)-1-phenyl-5-(trifluoromethyl)-1*H*-1,2,3-triazole-4-carboxamide hydrochloride [**1D(CF$_3$)**].

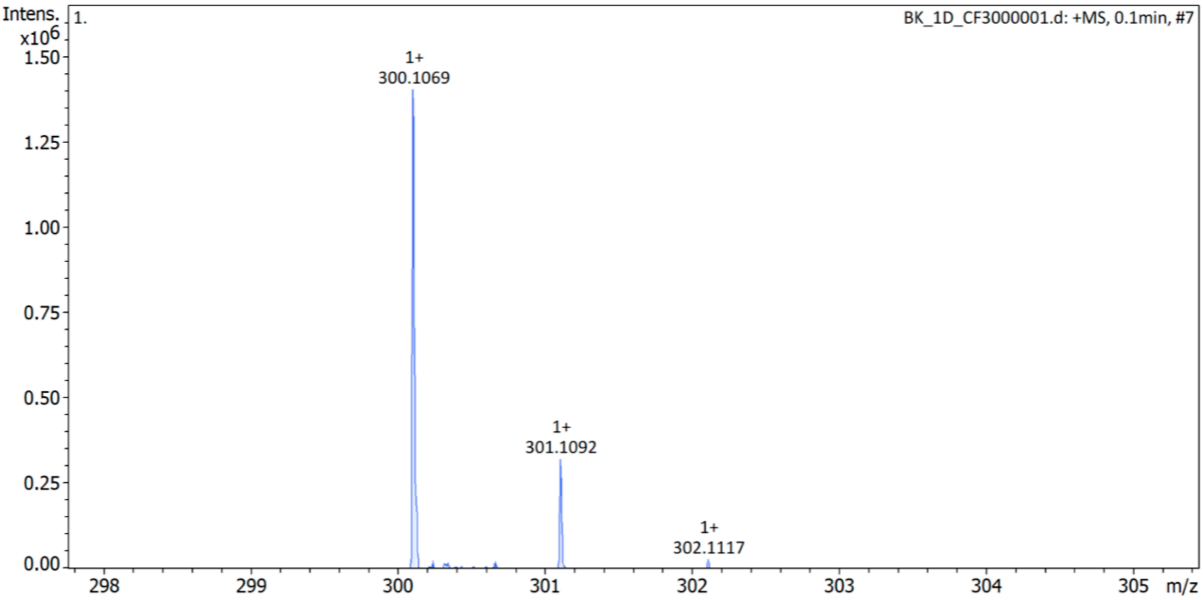

Figure S46. HRMS (ESI) spectrum of *N*-(2-aminoethyl)-1-phenyl-5-(trifluoromethyl)-1*H*-1,2,3-triazole-4-carboxamide hydrochloride [**1D(CF$_3$)**].

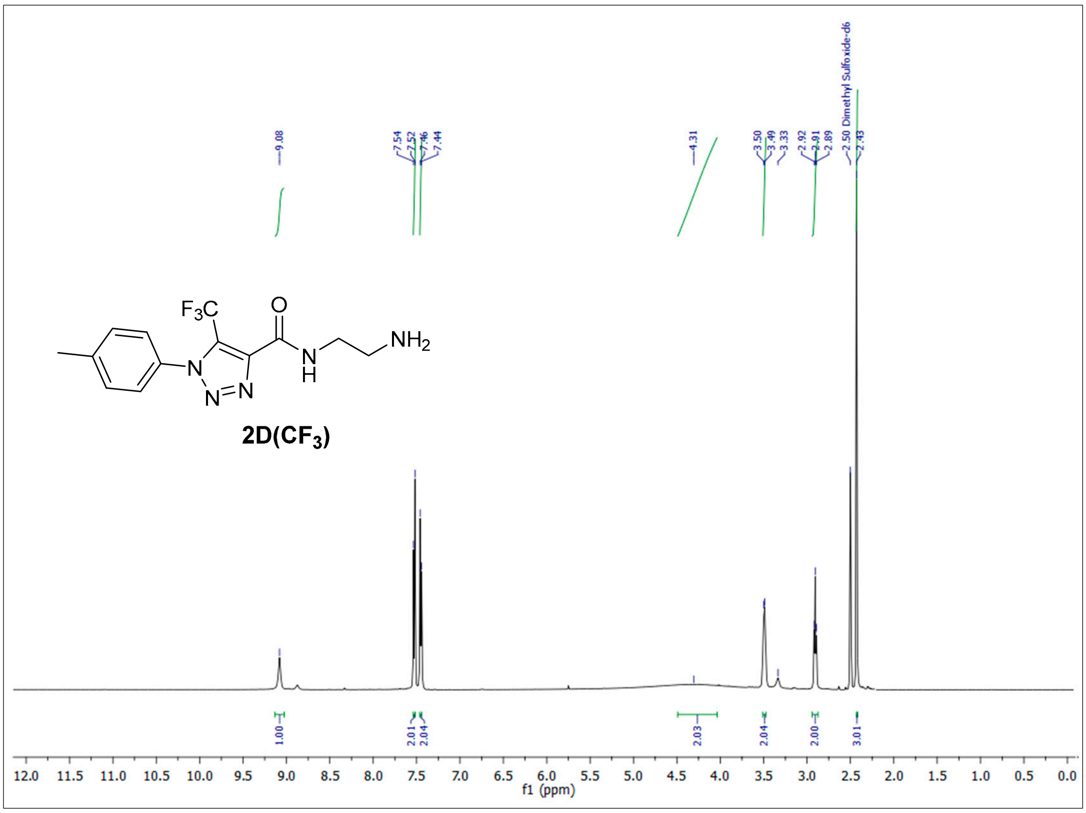

Figure S47. ^{1}H NMR (500 MHz, DMSO-d_6) spectrum of *N*-(2-aminoethyl)-1-(*p*-tolyl)-5-(trifluoromethyl)-1*H*-1,2,3-triazole-4-carboxamide [**2D(CF₃)**].

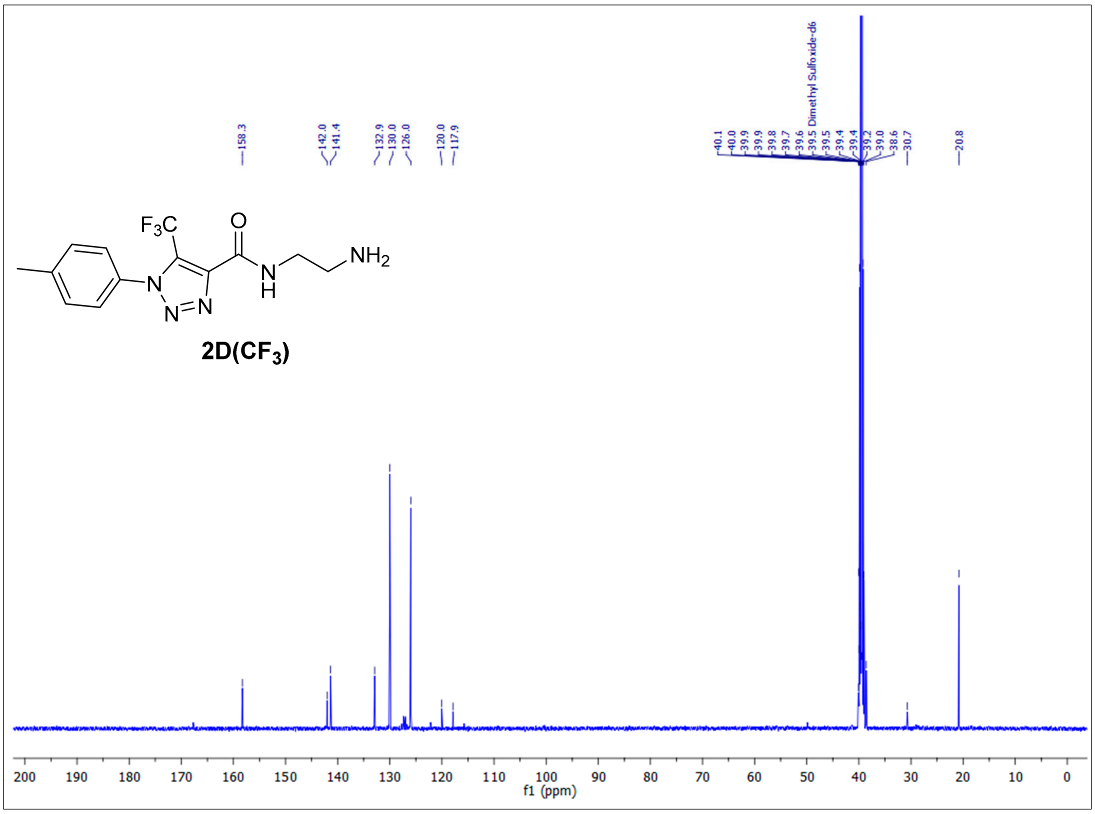

Figure S48. ^{13}C NMR (126 MHz, DMSO-d_6) spectrum of *N*-(2-aminoethyl)-1-(*p*-tolyl)-5-(trifluoromethyl)-1*H*-1,2,3-triazole-4-carboxamide [**2D(CF₃)**].

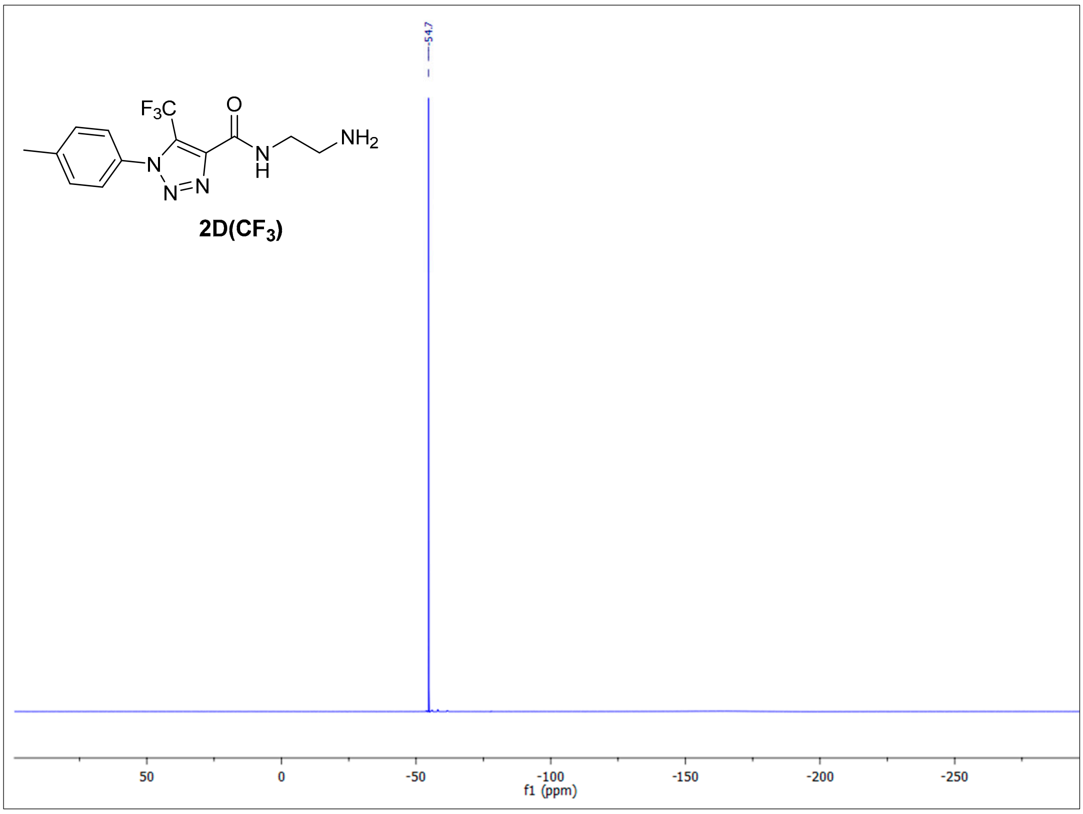

Figure S49. ^{19}F NMR (476 MHz, DMSO-d6) spectrum of *N*-(2-aminoethyl)-1-(*p*-tolyl)-5-(trifluoromethyl)-1*H*-1,2,3-triazole-4-carboxamide [**2D(CF3)**].

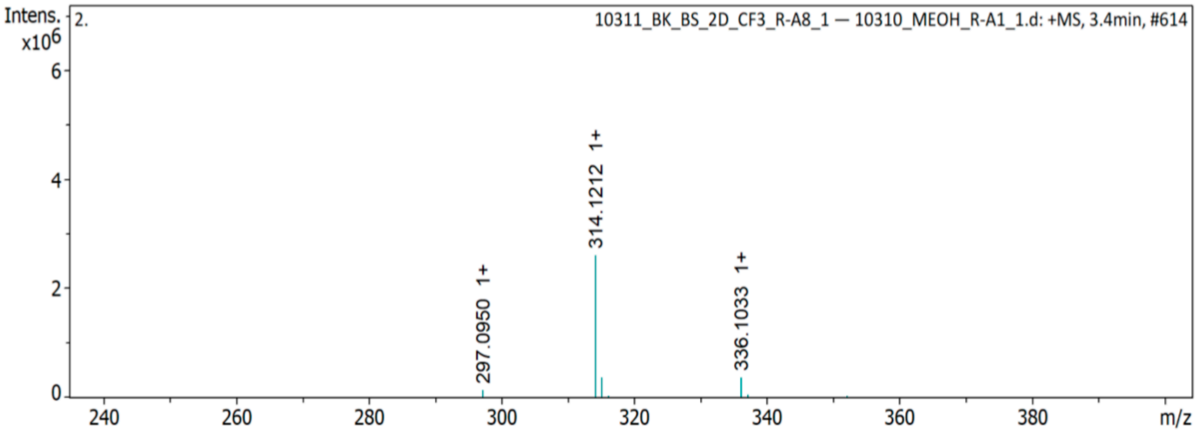

Figure S50. HRMS (ESI) spectrum of *N*-(2-aminoethyl)-1-(*p*-tolyl)-5-(trifluoromethyl)-1*H*-1,2,3-triazole-4-carboxamide [**2D(CF3)**].

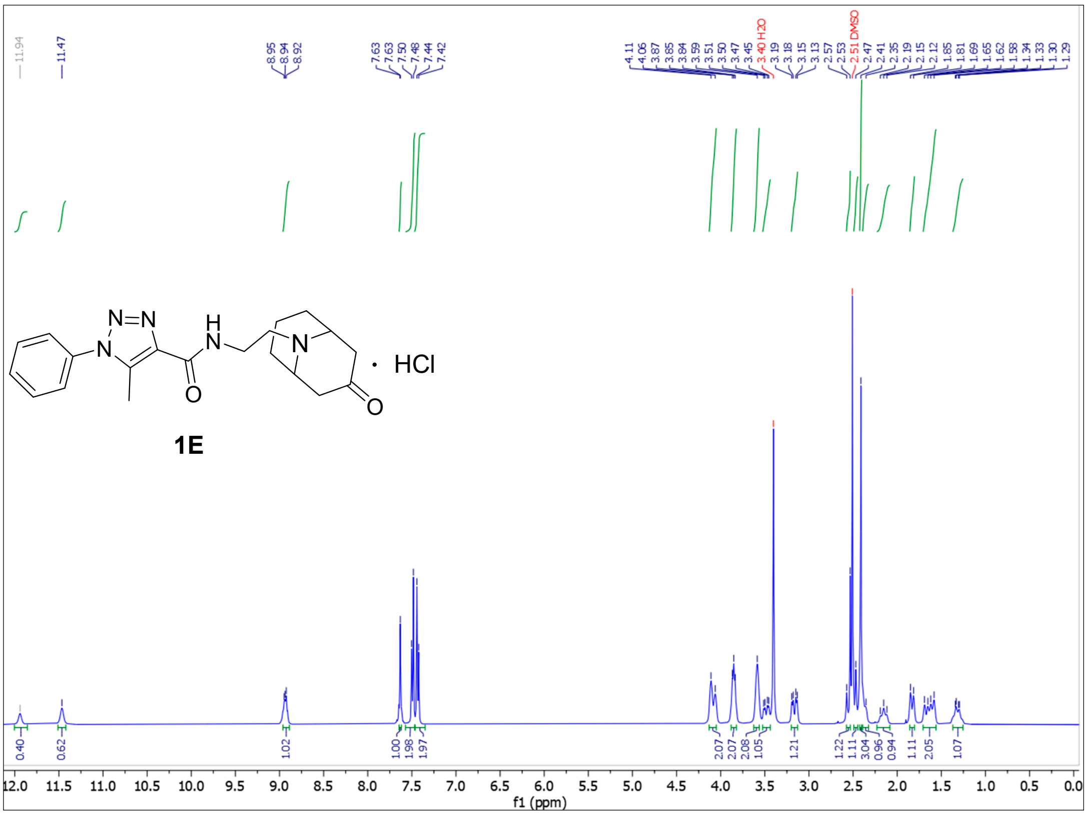

Figure S51. ^{1}H NMR (400 MHz, DMSO-d_6) spectrum of 5-methyl-*N*-(2-(3-oxo-9-azabicyclo[3.3.1]nonan-9-yl)ethyl)-1-phenyl-1*H*-1,2,3-triazole-4-carboxamide hydrochloride (**1E**).

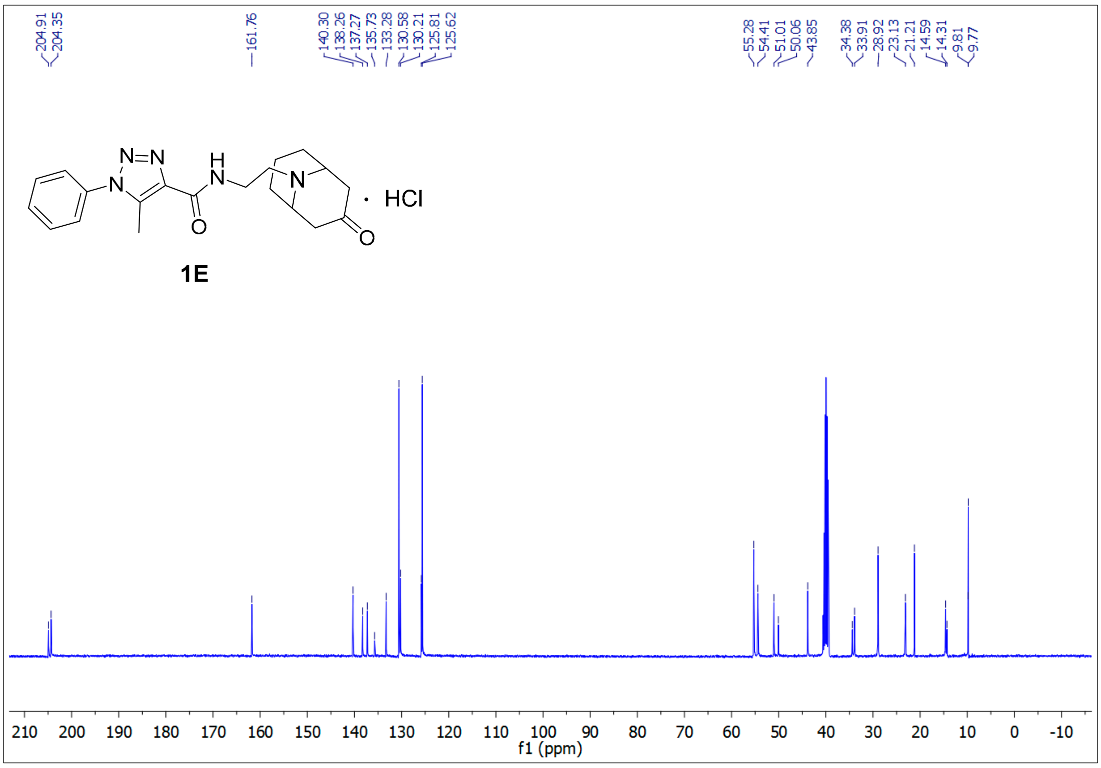

Figure S52. ^{13}C NMR (101 MHz, DMSO-d_6) spectrum of 5-methyl-*N*-(2-(3-oxo-9-azabicyclo[3.3.1]nonan-9-yl)ethyl)-1-phenyl-1*H*-1,2,3-triazole-4-carboxamide hydrochloride (**1E**).

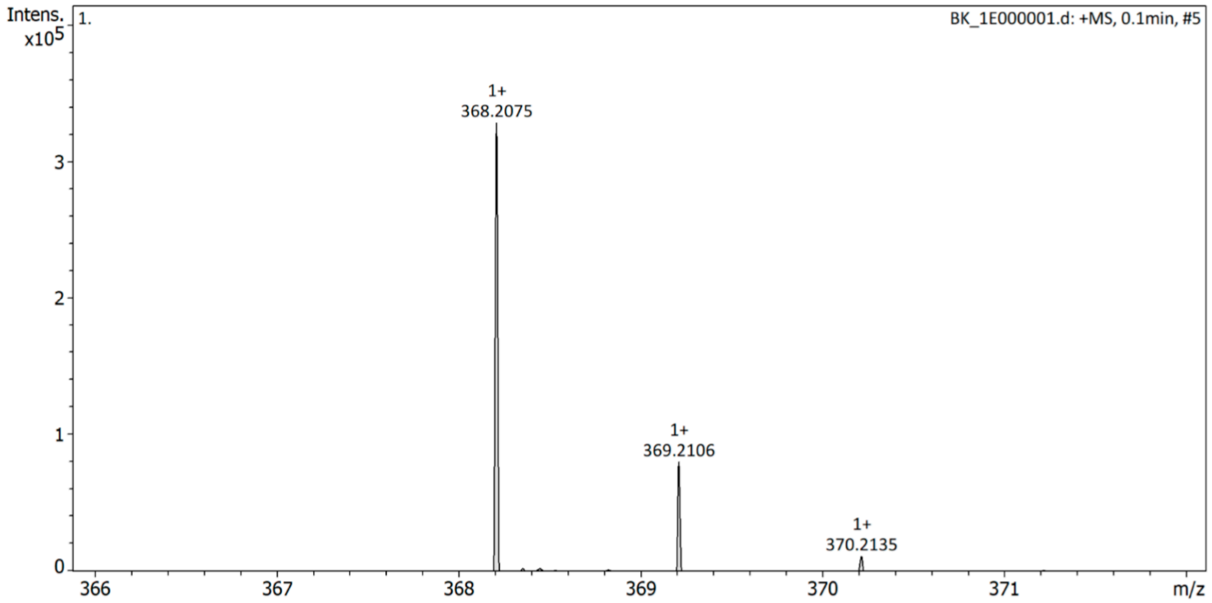

Figure S53. HRMS (ESI) spectrum of 5-methyl-*N*-(2-(3-oxo-9-azabicyclo[3.3.1]nonan-9-yl)ethyl)-1-phenyl-1*H*-1,2,3-triazole-4-carboxamide hydrochloride (**1E**).

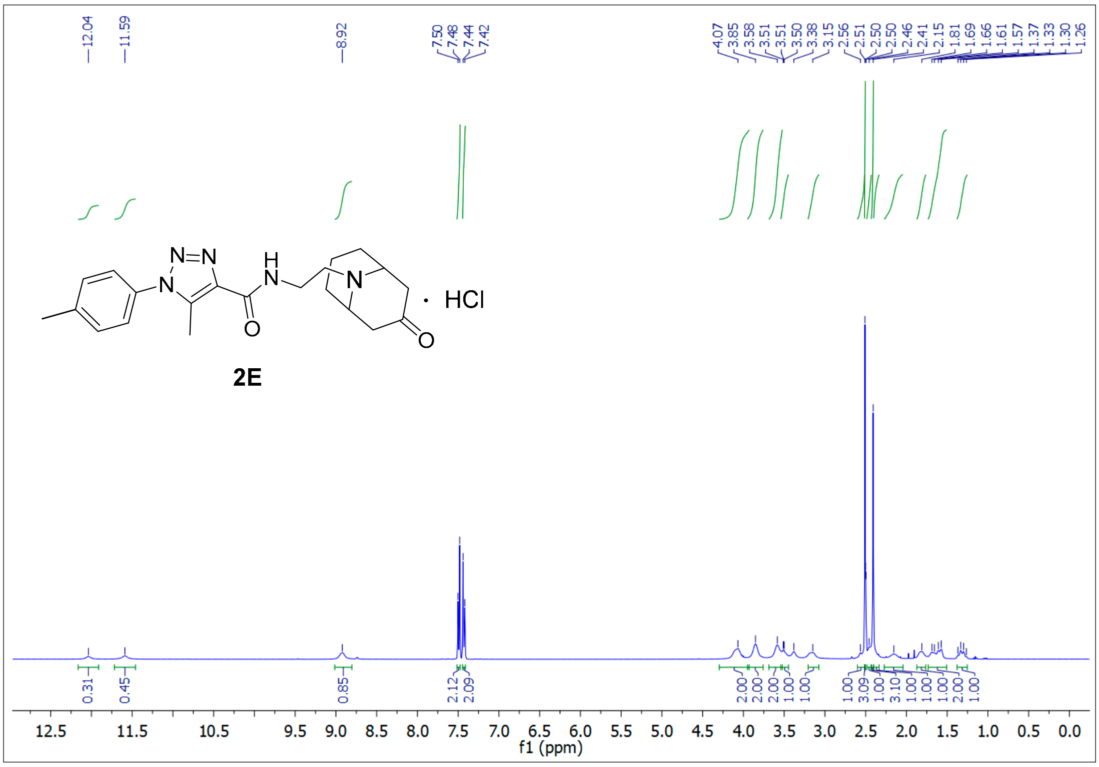

Figure S54. ^{1}H NMR (400 MHz, DMSO-d$_6$) spectrum of 5-methyl-*N*-(2-(3-oxo-9-azabicyclo[3.3.1]nonan-9-yl)ethyl)-1-(*p*-tolyl)-1*H*-1,2,3-triazole-4-carboxamide hydrochloride (**2E**).

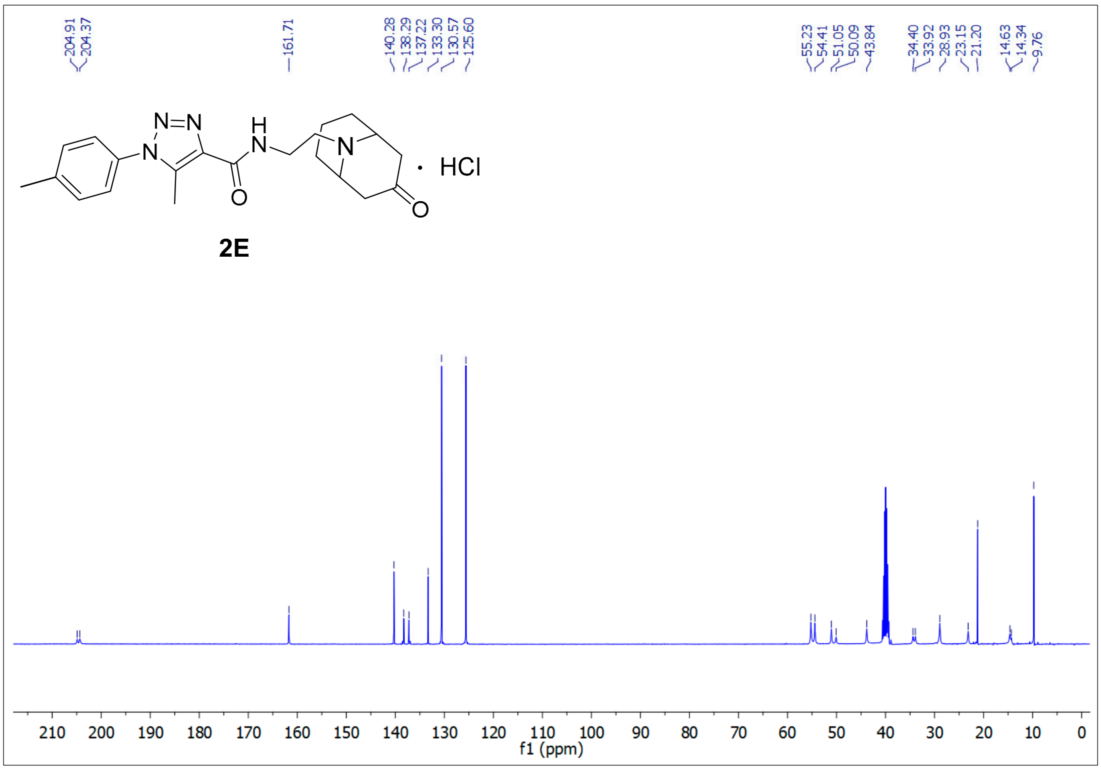

Figure S55. ^{13}C NMR (101 MHz, DMSO-d_6) spectrum of 5-methyl-*N*-(2-(3-oxo-9-azabicyclo[3.3.1]nonan-9-yl)ethyl)-1-(*p*-tolyl)-1*H*-1,2,3-triazole-4-carboxamide hydrochloride (**2E**).

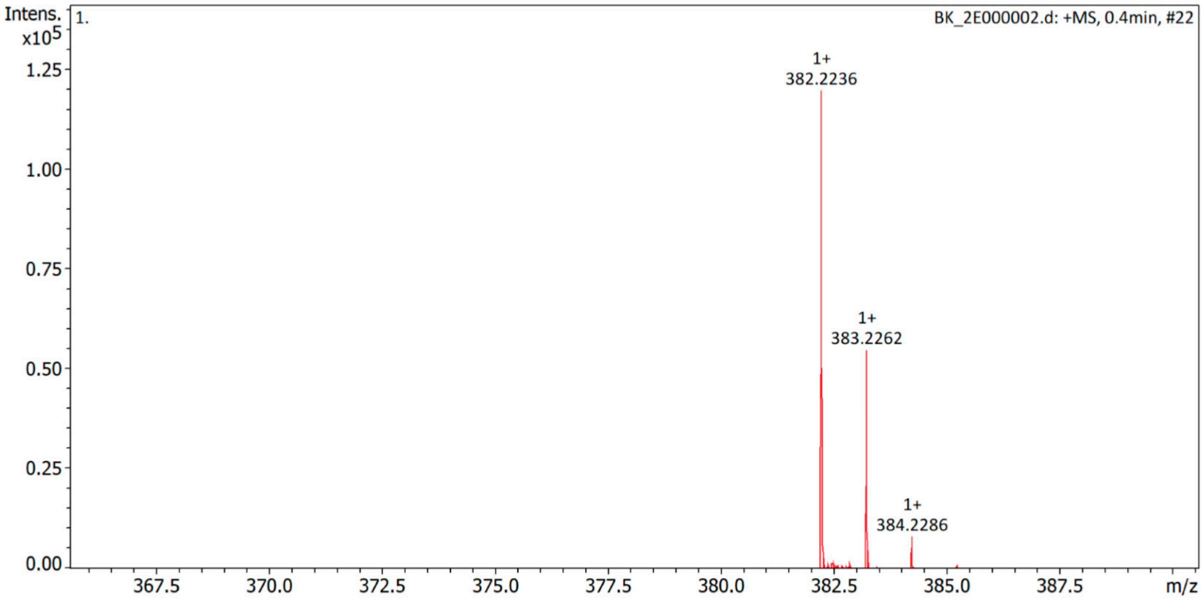

Figure S56. HRMS (ESI) spectrum of 5-methyl-*N*-(2-(3-oxo-9-azabicyclo[3.3.1]nonan-9-yl)ethyl)-1-(*p*-tolyl)-1*H*-1,2,3-triazole-4-carboxamide hydrochloride (**2E**).

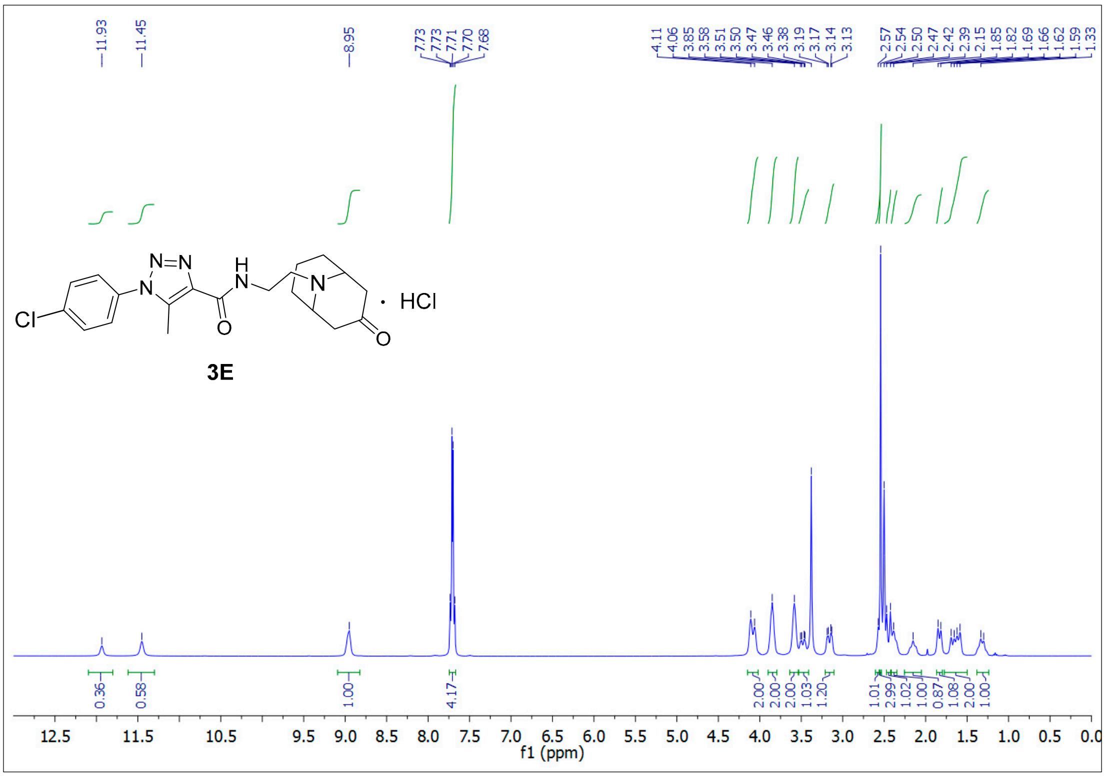

Figure S57. ^{1}H NMR (400 MHz, DMSO-d_6) spectrum of 1-(4-Chlorophenyl)-5-methyl-*N*-(2-(3-oxo-9-azabicyclo[3.3.1]nonan-9-yl)ethyl)-1*H*-1,2,3-triazole-4-carboxamide hydrochloride (**3E**).

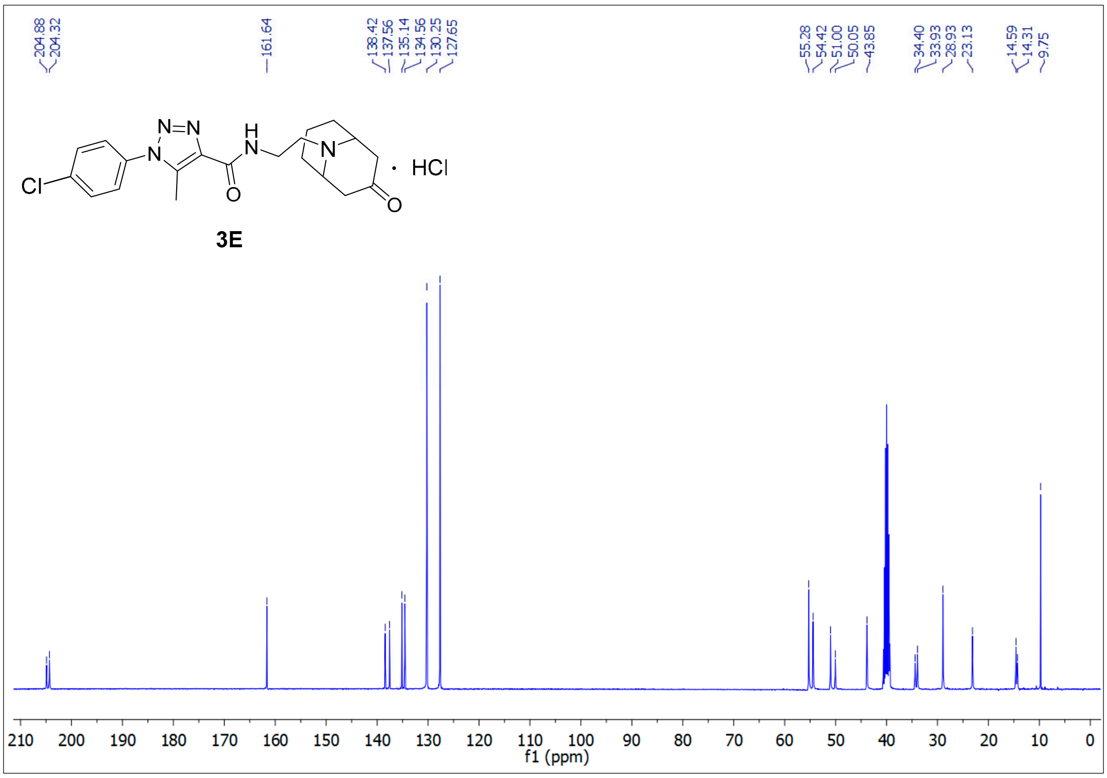

Figure S58. ^{13}C NMR (101 MHz, DMSO-d_6) spectrum of 1-(4-Chlorophenyl)-5-methyl-*N*-(2-(3-oxo-9-azabicyclo[3.3.1]nonan-9-yl)ethyl)-1*H*-1,2,3-triazole-4-carboxamide hydrochloride (**3E**).

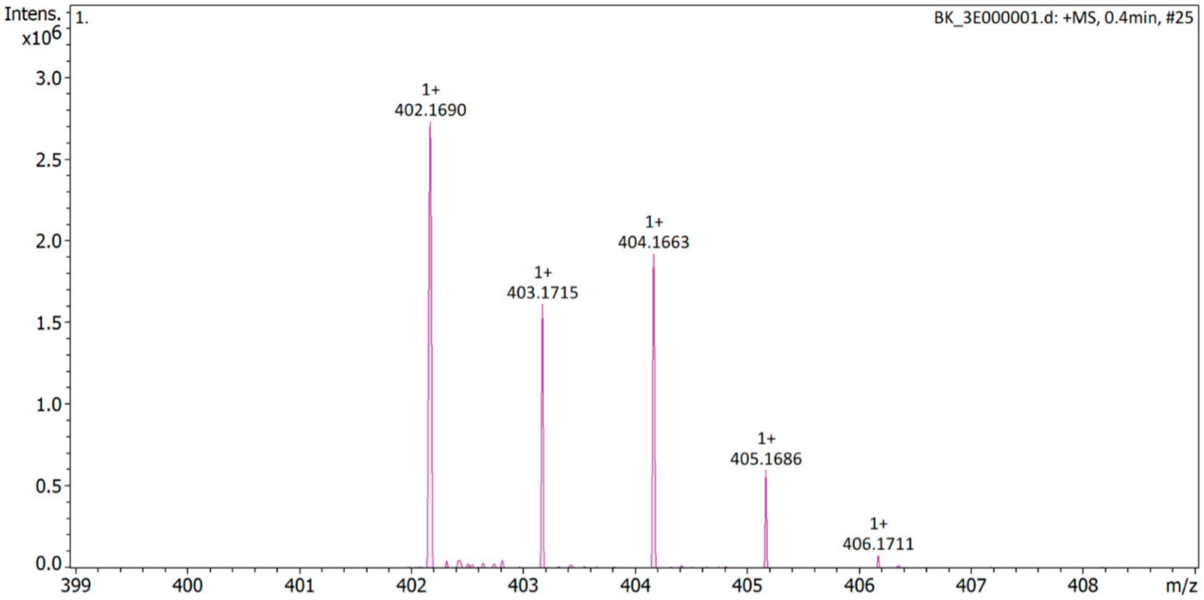

Figure S59. HRMS (ESI) spectrum of 1-(4-Chlorophenyl)-5-methyl-*N*-(2-(3-oxo-9-azabicyclo[3.3.1]nonan-9-yl)ethyl)-1*H*-1,2,3-triazole-4-carboxamide hydrochloride (**3E**).

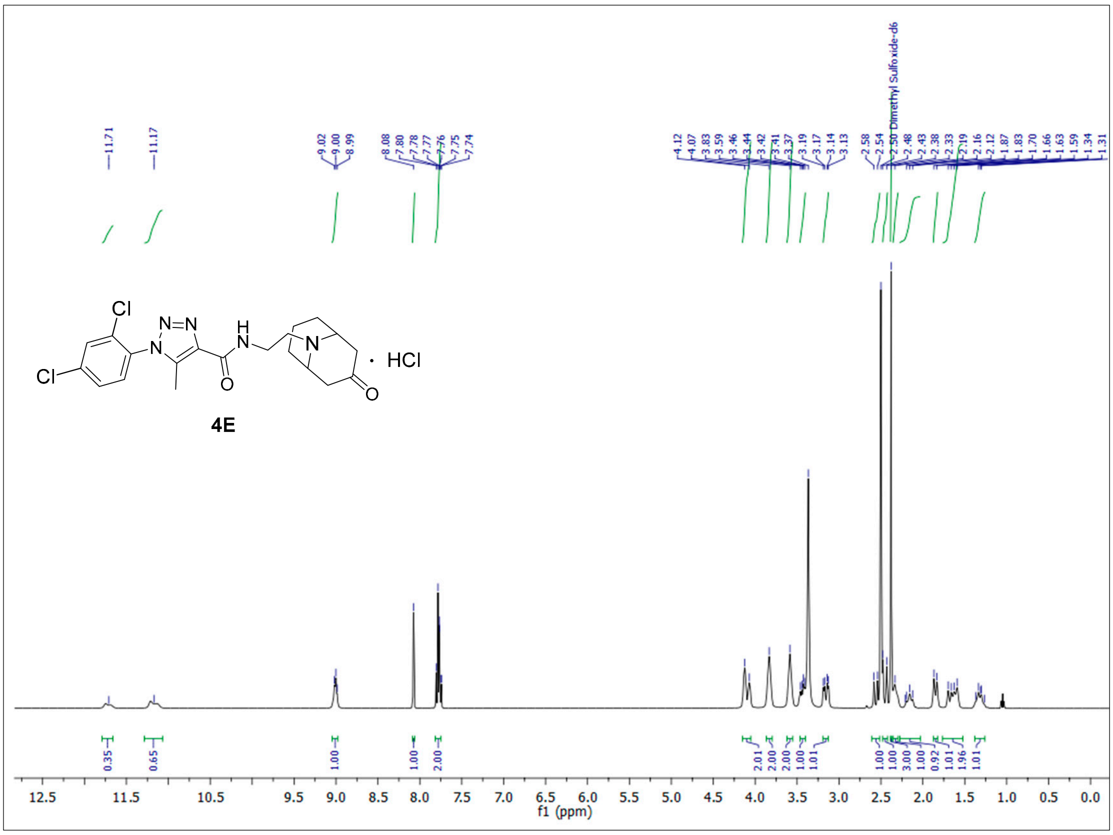

Figure S60. ^{1}H NMR (500 MHz, DMSO-d6) spectrum of 1-(2,4-dichlorophenyl)-5-methyl-*N*-(2-(3-oxo-9-azabicyclo[3.3.1]nonan-9-yl)ethyl)-1*H*-1,2,3-triazole-4-carboxamide hydrochloride (**4E**).

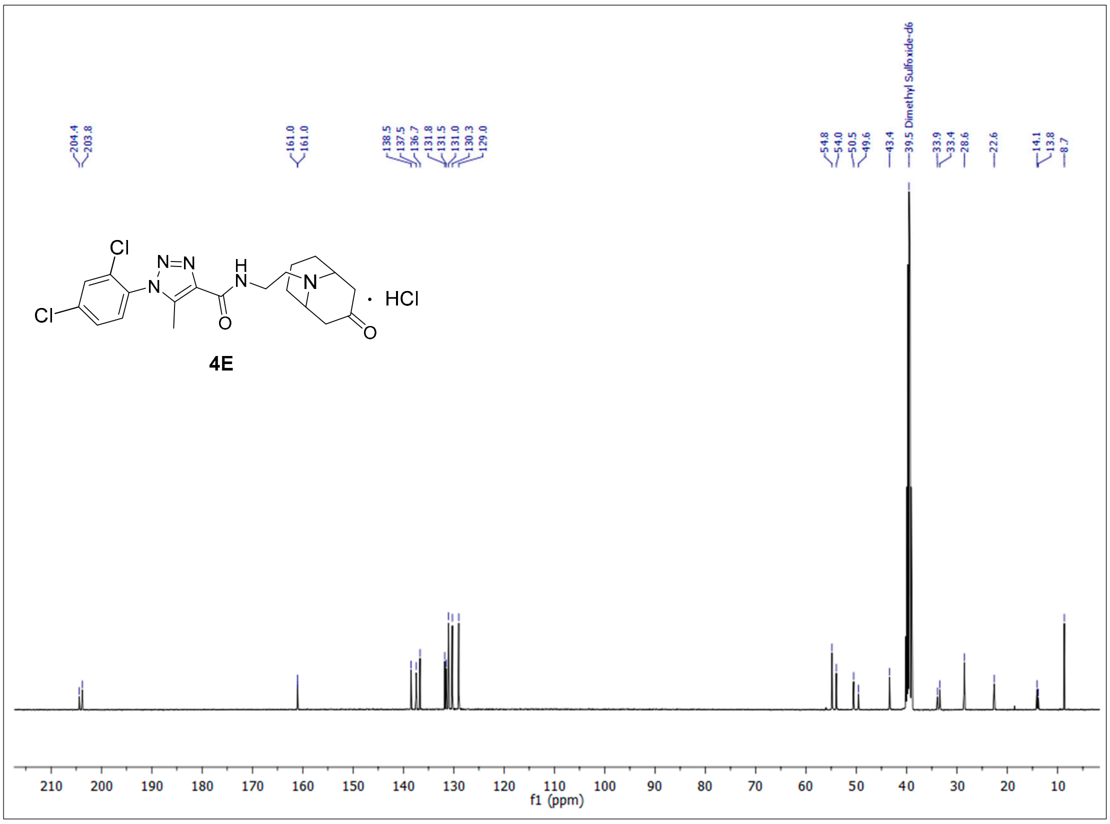

Figure S61. ^{13}C NMR (126 MHz, DMSO-d_6) spectrum of 1-(2,4-dichlorophenyl)-5-methyl-*N*-(2-(3-oxo-9-azabicyclo[3.3.1]nonan-9-yl)ethyl)-1*H*-1,2,3-triazole-4-carboxamide hydrochloride (**4E**).

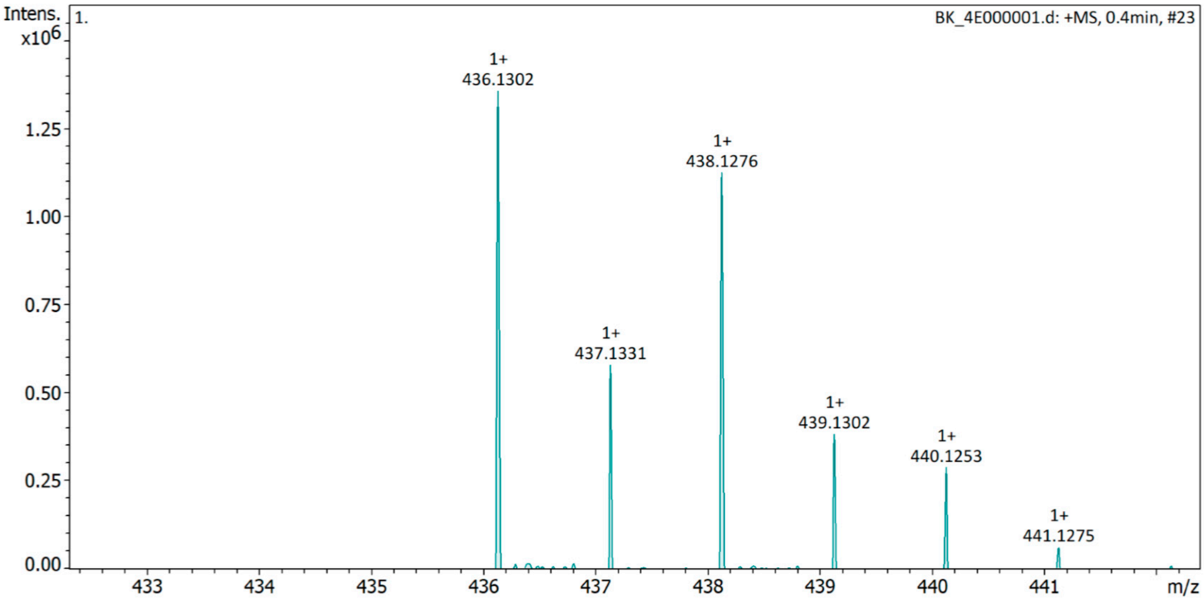

Figure S62. HRMS (ESI) spectrum of 1-(2,4-dichlorophenyl)-5-methyl-*N*-(2-(3-oxo-9-azabicyclo[3.3.1]nonan-9-yl)ethyl)-1*H*-1,2,3-triazole-4-carboxamide hydrochloride (**4E**).

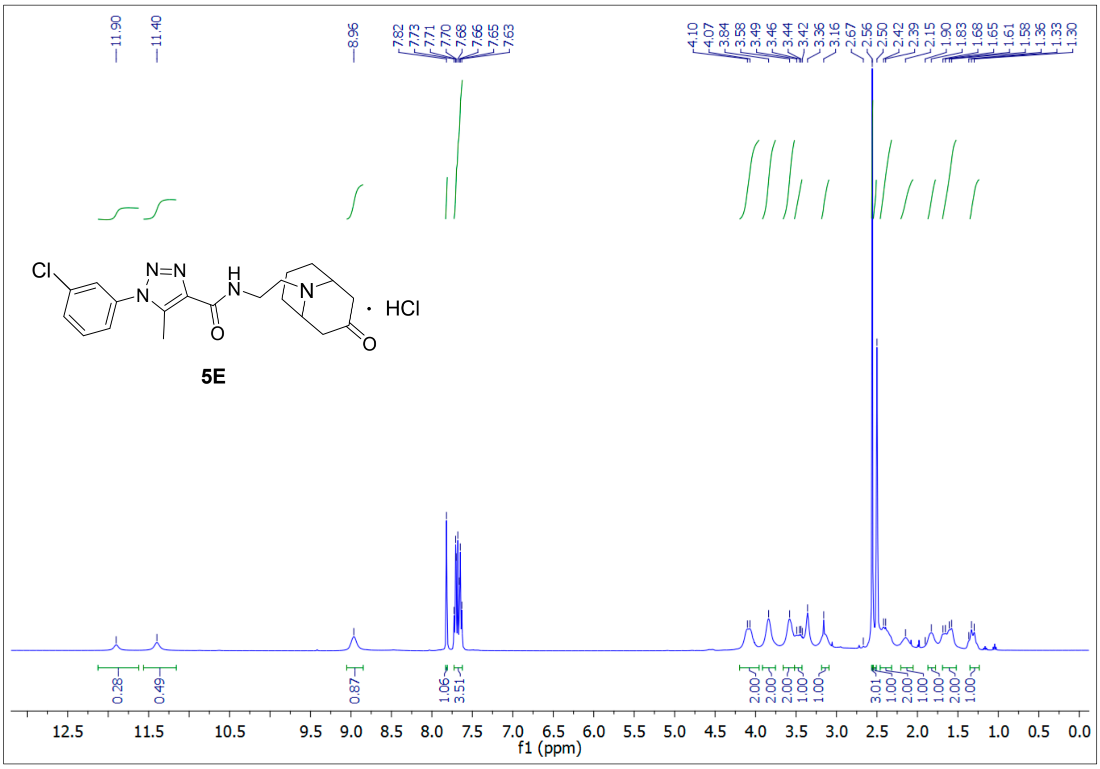

Figure S63. ^{1}H NMR (400 MHz, DMSO-d_6) spectrum of 1-(3-chlorophenyl)-5-methyl-*N*-(2-(3-oxo-9-azabicyclo[3.3.1]nonan-9-yl)ethyl)-1*H*-1,2,3-triazole-4-carboxamide hydrochloride (**5E**).

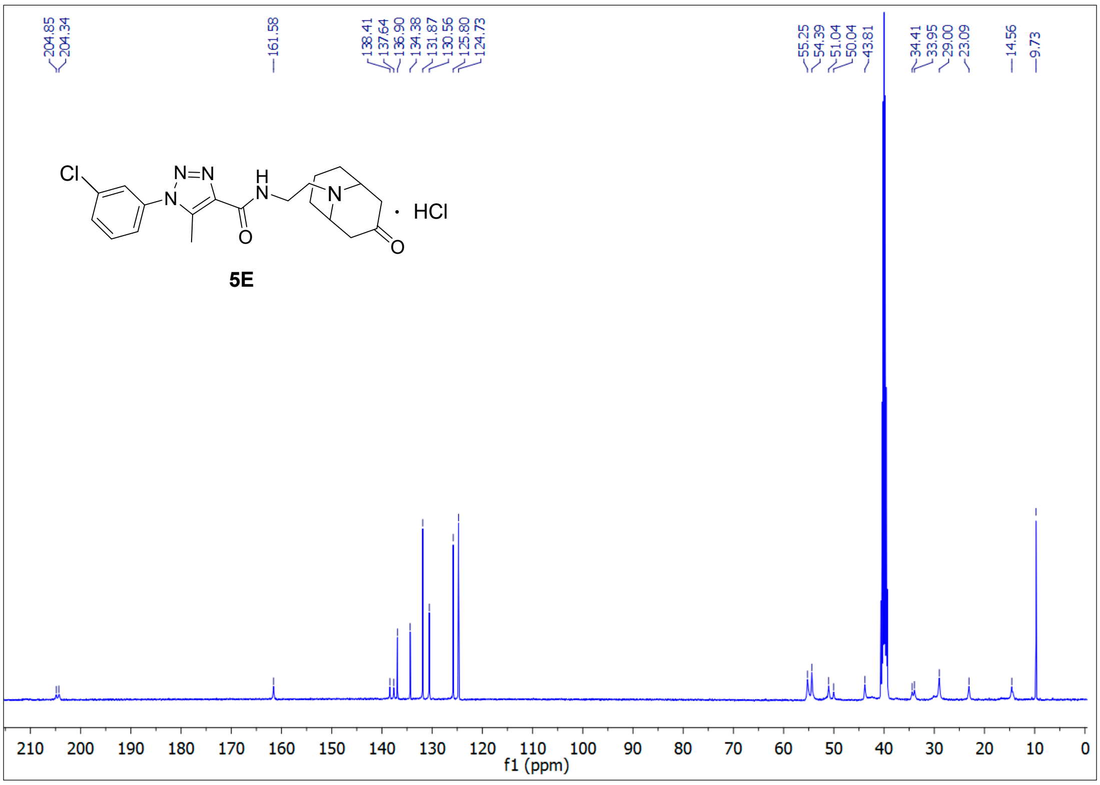

Figure S64. ^{13}C NMR (101 MHz, DMSO-d_6) spectrum of 1-(3-chlorophenyl)-5-methyl-*N*-(2-(3-oxo-9-azabicyclo[3.3.1]nonan-9-yl)ethyl)-1*H*-1,2,3-triazole-4-carboxamide hydrochloride (**5E**).

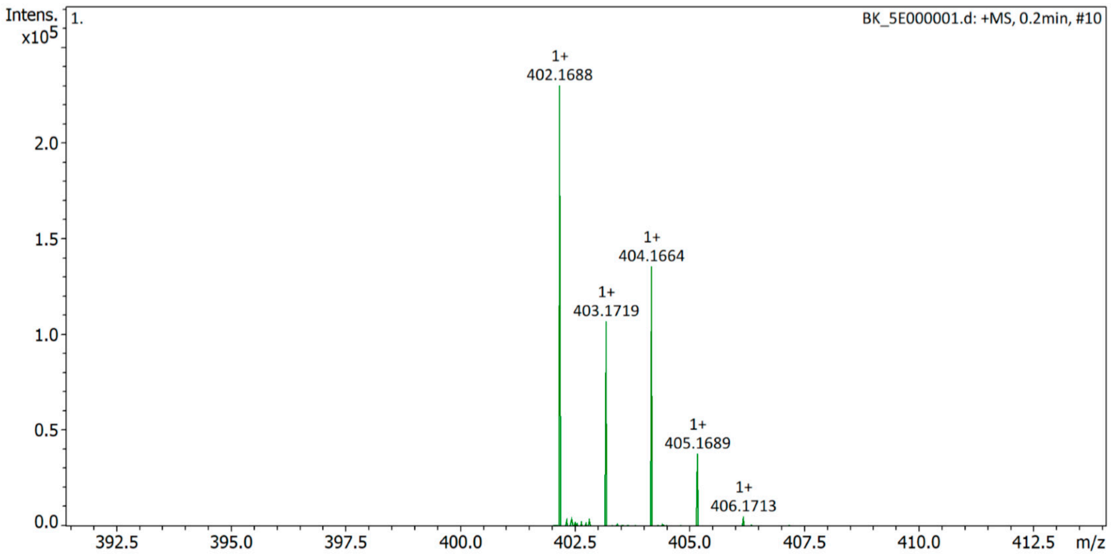

Figure S65. HRMS (ESI) spectrum of 1-(3-chlorophenyl)-5-methyl-*N*-(2-(3-oxo-9-azabicyclo[3.3.1]nonan-9-yl)ethyl)-1*H*-1,2,3-triazole-4-carboxamide hydrochloride (**5E**).

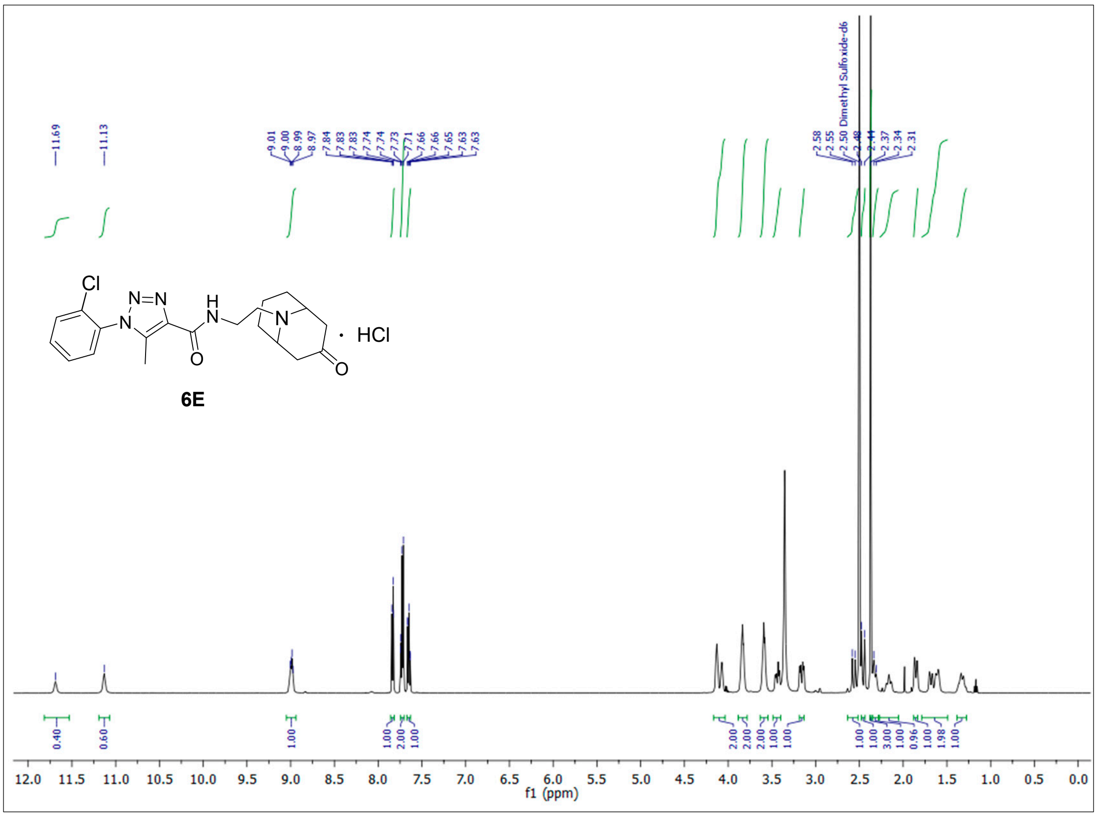

Figure S66. ^{1}H NMR (500 MHz, DMSO-d$_6$) spectrum of 1-(2-chlorophenyl)-5-methyl-*N*-(2-(3-oxo-9-azabicyclo[3.3.1]nonan-9-yl)ethyl)-1*H*-1,2,3-triazole-4-carboxamide hydrochloride (**6E**).

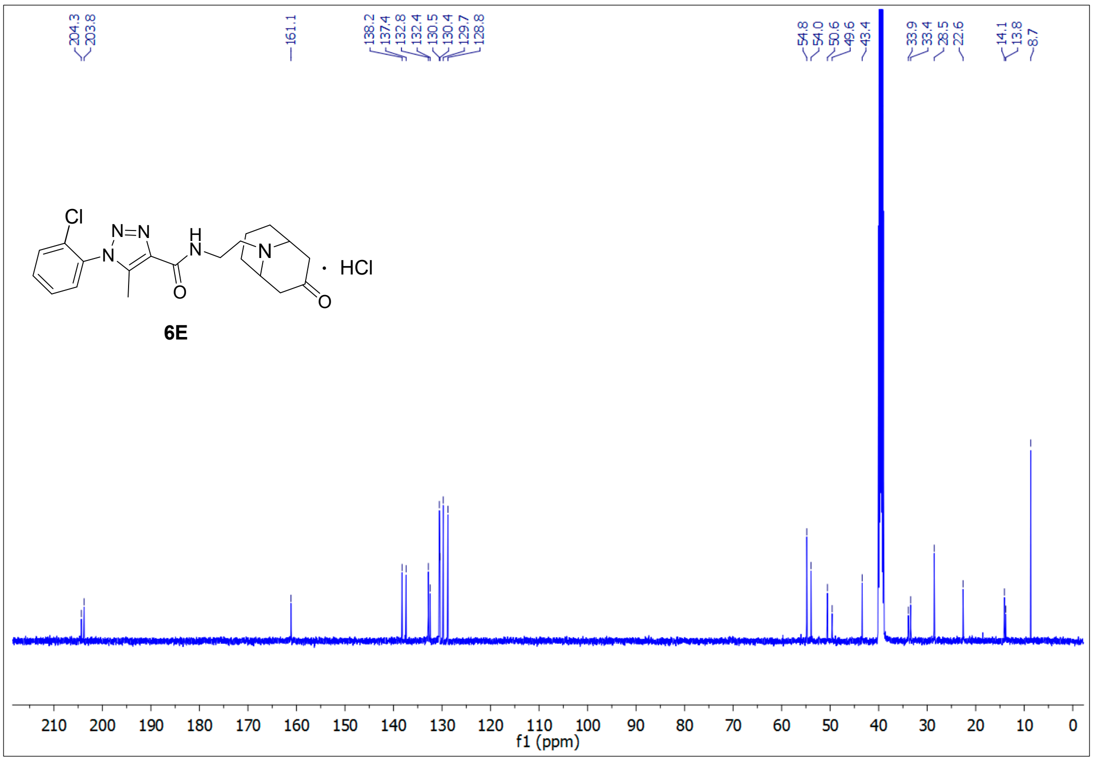

Figure S67. ^{13}C NMR (126 MHz, DMSO-d_6) spectrum of 1-(2-chlorophenyl)-5-methyl-*N*-(2-(3-oxo-9-azabicyclo[3.3.1]nonan-9-yl)ethyl)-1*H*-1,2,3-triazole-4-carboxamide hydrochloride (**6E**).

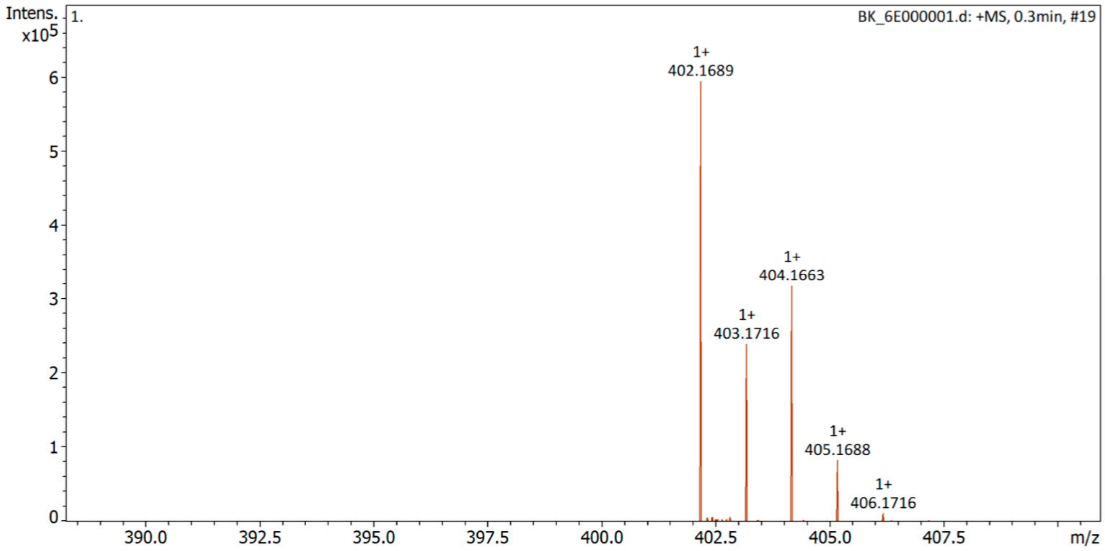

Figure S68. HRMS (ESI) spectrum of 1-(2-chlorophenyl)-5-methyl-*N*-(2-(3-oxo-9-azabicyclo[3.3.1]nonan-9-yl)ethyl)-1*H*-1,2,3-triazole-4-carboxamide hydrochloride (**6E**).

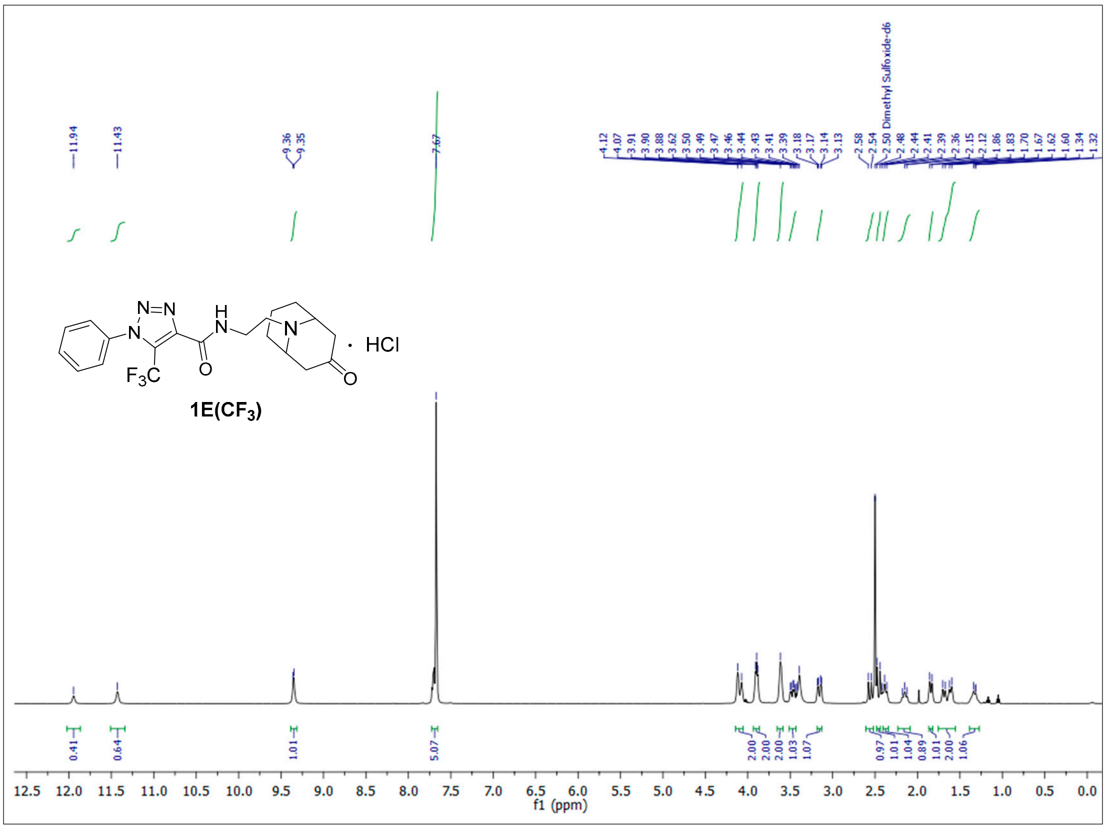

Figure S69. ^{1}H NMR (500 MHz, DMSO-d_6) spectrum of *N*-(2-(3-Oxo-9-azabicyclo[3.3.1]nonan-9-yl)ethyl)-1-phenyl-5-(trifluoromethyl)-1*H*-1,2,3-triazole-4-carboxamide hydrochloride [**1E(CF$_3$)**].

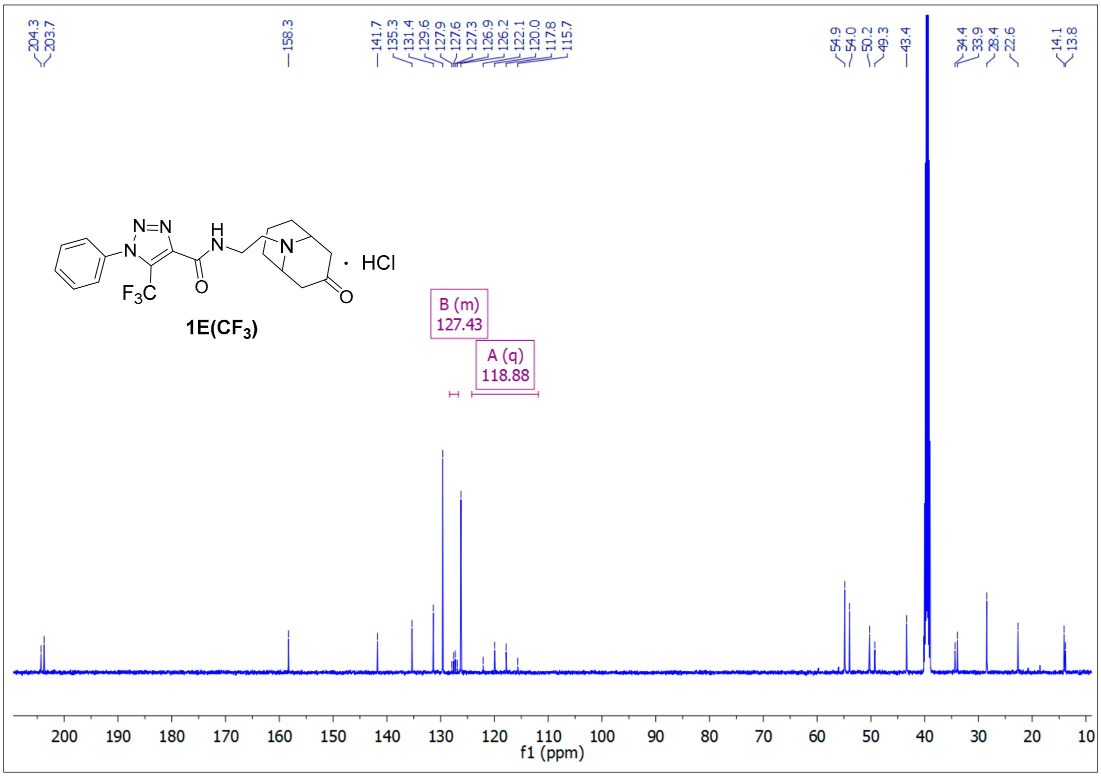

Figure S70. ^{13}C NMR (126 MHz, DMSO-d_6) spectrum of *N*-(2-(3-Oxo-9-azabicyclo[3.3.1]nonan-9-yl)ethyl)-1-phenyl-5-(trifluoromethyl)-1*H*-1,2,3-triazole-4-carboxamide hydrochloride [**1E(CF$_3$)**].

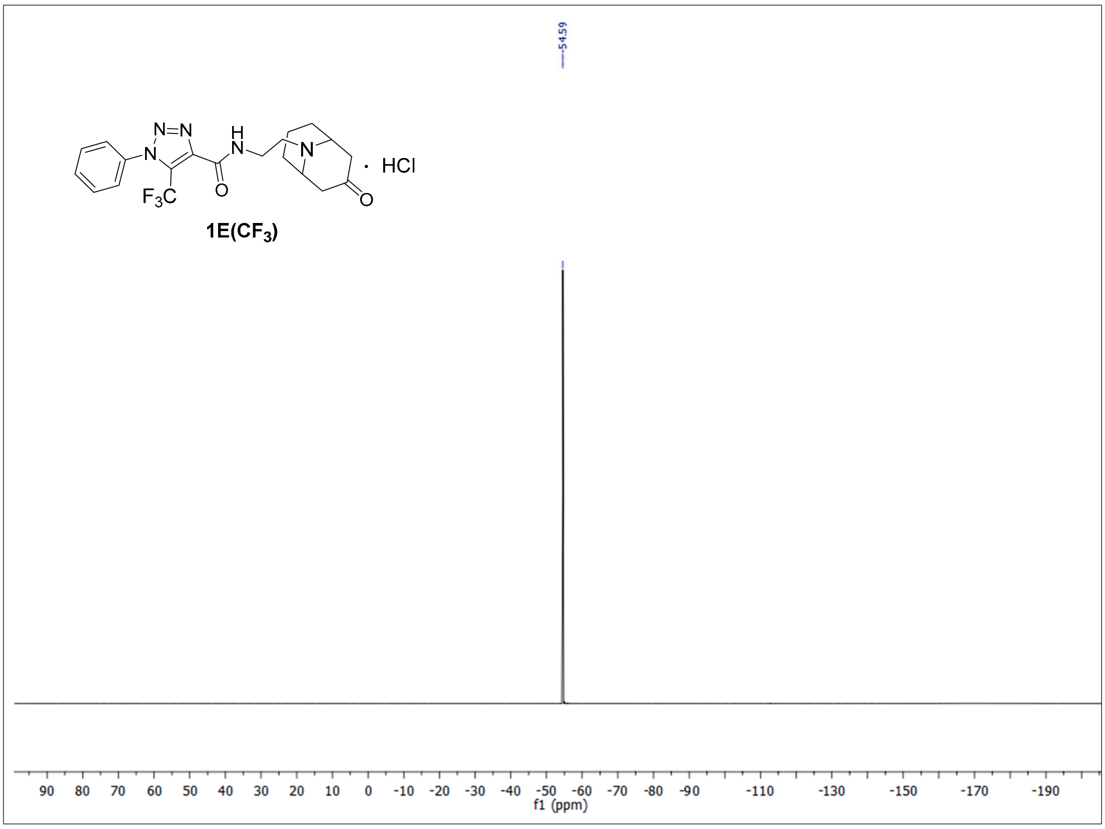

Figure S71. ^{19}F NMR (476 MHz, DMSO-d_6) spectrum of *N*-(2-(3-Oxo-9-azabicyclo[3.3.1]nonan-9-yl)ethyl)-1-phenyl-5-(trifluoromethyl)-1*H*-1,2,3-triazole-4-carboxamide hydrochloride [**1E(CF$_3$)**].

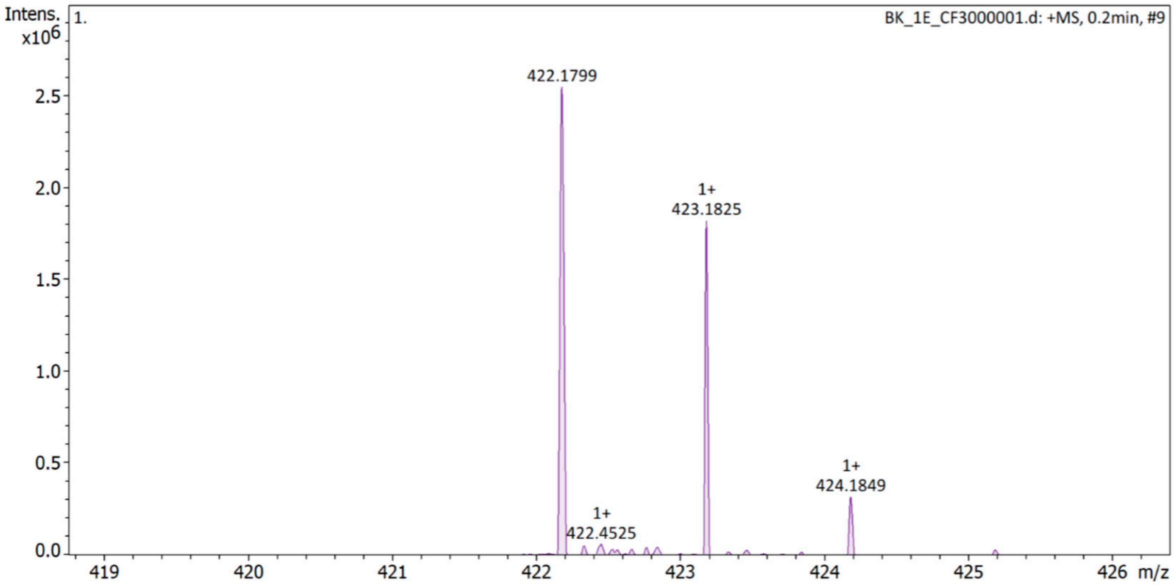

Figure S72. HRMS (ESI) spectrum of *N*-(2-(3-Oxo-9-azabicyclo[3.3.1]nonan-9-yl)ethyl)-1-phenyl-5-(trifluoromethyl)-1*H*-1,2,3-triazole-4-carboxamide hydrochloride [**1E(CF$_3$)**].

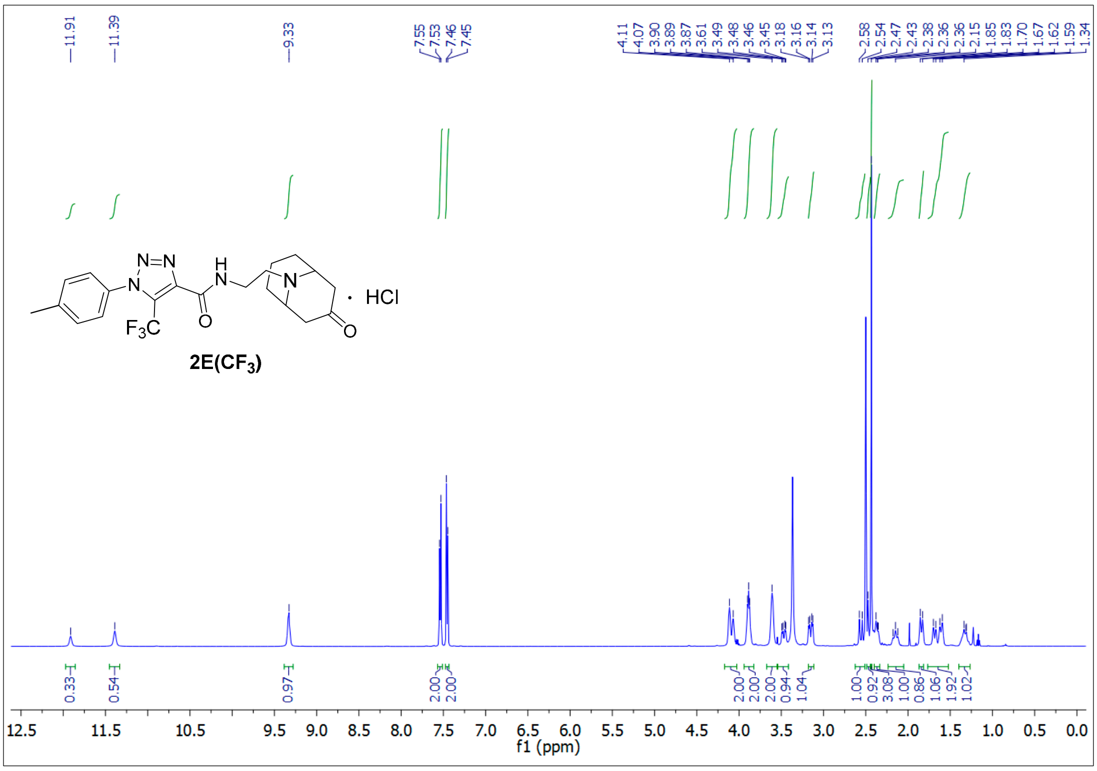

Figure S73. ^{1}H NMR (500 MHz, DMSO-d$_6$) spectrum of *N*-(2-(3-Oxo-9-azabicyclo[3.3.1]nonan-9-yl)ethyl)-1-(*p*-tolyl)-5-(trifluoromethyl)-1*H*-1,2,3-triazole-4-carboxamide hydrochloride [**2E(CF$_3$)**].

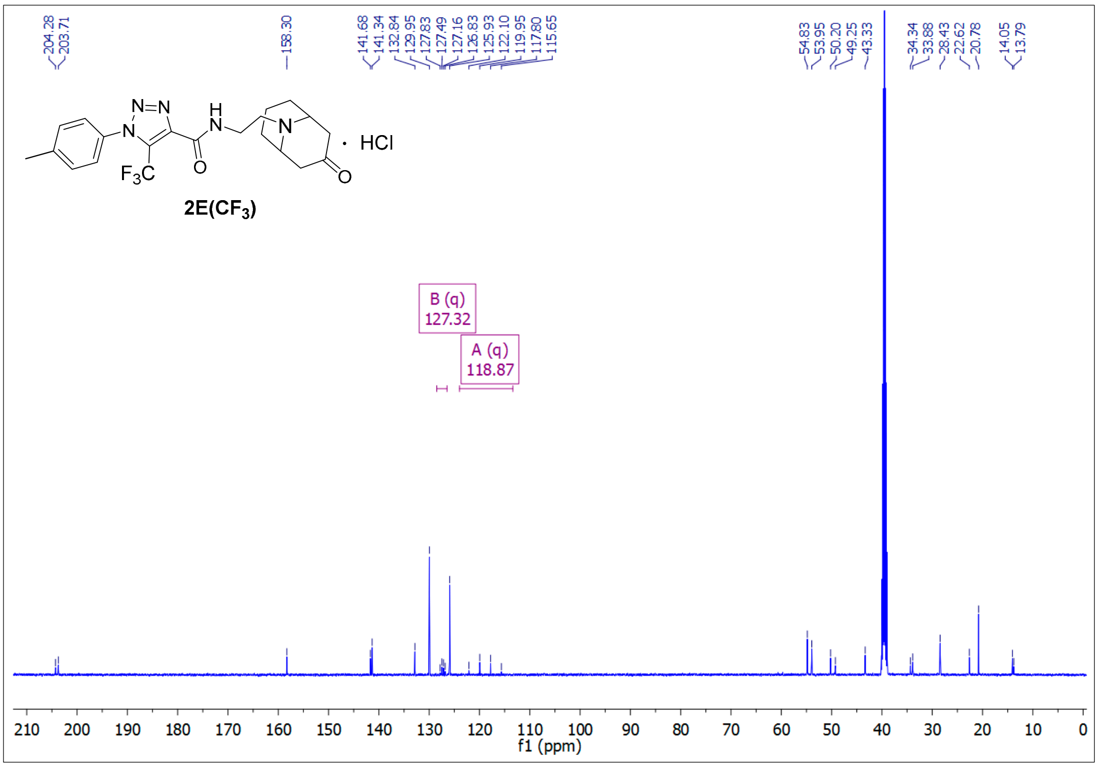

Figure S74. ^{13}C NMR (126 MHz, DMSO-d$_6$) spectrum of *N*-(2-(3-Oxo-9-azabicyclo[3.3.1]nonan-9-yl)ethyl)-1-(*p*-tolyl)-5-(trifluoromethyl)-1*H*-1,2,3-triazole-4-carboxamide hydrochloride [**2E(CF$_3$)**].

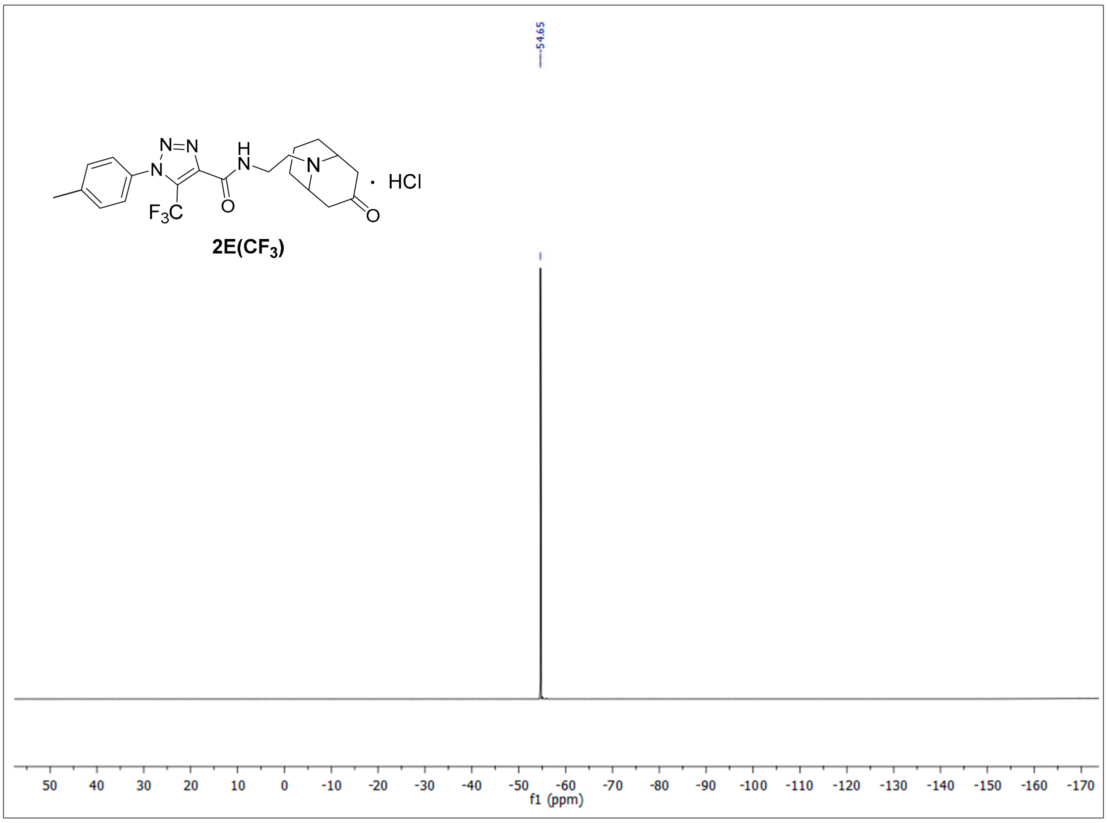

Figure S75. ^{19}F NMR (476 MHz, DMSO-d_6) spectrum of *N*-(2-(3-Oxo-9-azabicyclo[3.3.1]nonan-9-yl)ethyl)-1-(*p*-tolyl)-5-(trifluoromethyl)-1*H*-1,2,3-triazole-4-carboxamide hydrochloride [**2E(CF$_3$)**].

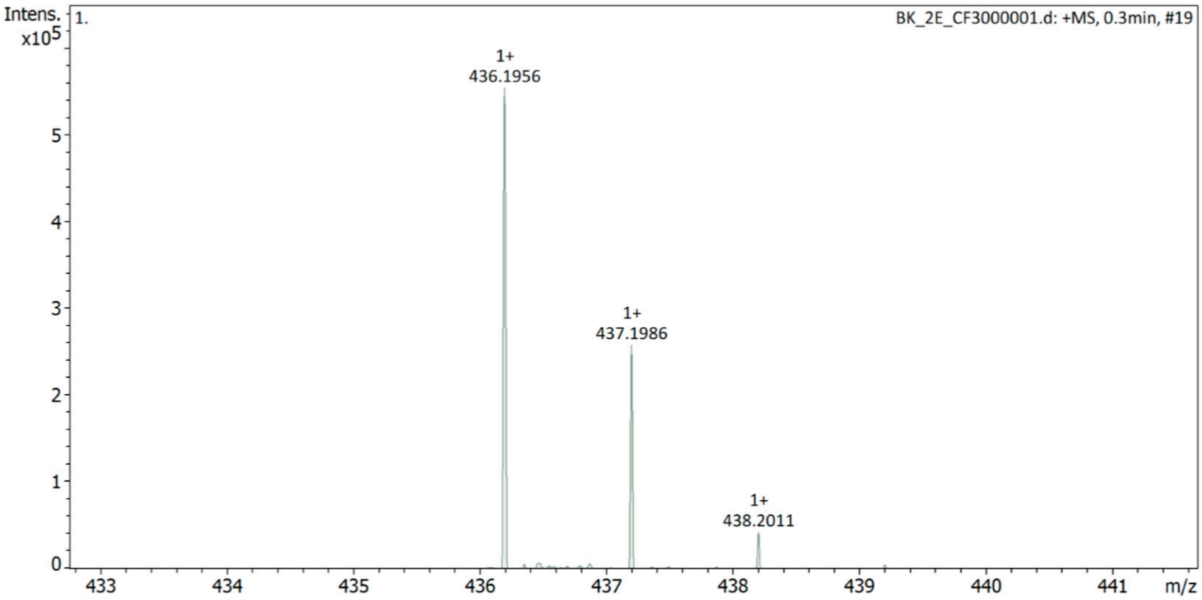

Figure S76. HRMS (ESI) spectrum of *N*-(2-(3-Oxo-9-azabicyclo[3.3.1]nonan-9-yl)ethyl)-1-(*p*-tolyl)-5-(trifluoromethyl)-1*H*-1,2,3-triazole-4-carboxamide hydrochloride [**2E(CF$_3$)**].
